# Supplementary material for: Effects of health and social care spending constraints on mortality in England: a time trend analysis
Source: BMJ Open. 2017 Nov 15;7(11):e017722. doi: 10.1136/bmjopen-2017-017722 (PMC5719267; doi:10.1136/bmjopen-2017-017722)
Supplement: Supplementary file 1 [file bmjopen-2017-017722supp001.pdf]

SUPPLEMENTARY APPENDIX

Data collection and generation

Age-standardized rates

All-cause age-standardized death rates (ASDR) were calculated using the ONS' standardized mortality rate calculation template with the standard European population.[1] The ten age groups used were: 0-34, 35-49, 50-54, 55-59, 60-64, 65-69, 70-74, 75-79, 80-84, and ≥85.

Age-standardized potential years of life lost rates were calculated using the same age groups and by weighting the number of 'amenable' deaths in a given year by the standard European population and the number of additional years that the deceased might have been expected to live in the presence of timely and effective healthcare.[2]

Spending data

To calculate the 2014/15 data point, we used the percentage change in the bridging data between 2013/14 and 2014/15. From this, we calculated real PEH per capita in units of £10. Planned spending from 2015/16 to 2020/21 for healthcare was sourced from the 2015 Spending Review and Autumn Statement.[5] For adult social care spending, no consolidated planned expenditure limit was available. Therefore, we defined 2015/16 to 2020/21 planned real PES based on the continuation of –2.25% change in core PES in 2014/15 supplemented by the potential revenue from the adult social care precept, which is intended to raise funds through 2% per annum increases in local government tax revenues.[6]

Deprivation data

Indices of Multiple Deprivation (IMD), a composite measure using data on topics such as employment, education, and crime, were provided for each local government area by the UK Department for Communities and Local Government for 2010[7]. Population-weighted deprivation status was provided as the average rank of the ranks of the five IMD variables, ranging from 1 to 325, in order of decreasing deprivation.

Resource data

Health and social care staff and bed numbers (resource data) available for 2001 to 2014 were obtained for the following: the number of NHS hospital doctors; general practitioners (GPs); qualified NHS health and social care nurses; qualified NHS scientific, therapeutic and technical staff; NHS ambulance staff; NHS health and social care clinical support staff; NHS infrastructure support staff[8,9]; the number of overnight beds in NHS hospitals[10]; and the numbers of staff providing social care with accommodation and without (see Supplementary Appendix)[11,12].

Employment data in social work activities with accommodation (e.g. care homes) and without accommodation provision (e.g. home care) in England between 2001 and 2014 were obtained from the UK labour market statistics provided by the ONS[11,12]. The 2003 version of Standard Industrial Classification (SIC) codes (SIC 2003) were used to identify businesses conducting social work activities with and without accommodation (SIC 2003 code: 8531 and 8532, respectively). Prior to 2009, data was available from the Annual Business Enquiry, whereas the Business Register Employment Survey provided data from 2009 onwards under the SIC 2007 codes. Proportional mapping using ONS-published proportions between SIC 2003 and SIC 2007 was conducted to estimate the number of employment which corresponds to the aforementioned SIC 2003 codes between 2009 and 2014.[13]

Time-trend prediction analysis

Mortality and potential years of life lost

Given that consistent mortality data were available from 2001 onwards, and that the reductions in real PEH and PES occurred in 2010/11, we used 2001–2010 as an observation base on which to build our time-trend model for predicting mortality rates in 2011–2014.

Depending on whether overdispersion was observed (where the variance exceeds the mean), we resolved to use either a Poisson or quasi-Poisson regression model, the latter of which incorporates a scaling parameter in order to address overdispersion. In every dataset for which we conducted time-series modelling, the variance of the mortality rates exceeded the mean rate.

Therefore, we employed a quasi-Poisson model in all analyses, with the logarithmic link function preventing the prediction of negative rates. The model takes the following form:

$$E(M_t) = \exp(\alpha + \beta t), \qquad (1)$$

where $E(M_t)$ is the estimated mortality rate for year t, α is the baseline mortality rate, and β is the estimated drift parameter. For increasing observation-base trends, the following non-linear prediction model proposed by Dyba and colleagues,[14] also assuming a Poisson or quasi-Poisson distribution, was used:

$$E(M_t) = \alpha(1 + \beta t). \qquad (2)$$

The trend of a mortality rate was determined to be decreasing or increasing according to whether the geometric mean of the ratio of the annual percentage change was negative or positive, respectively.

Rate ratios for the observed and expected mortality rates were calculated. We also estimated the number of "excess" (higher-than-expected) or "prevented" (lower-than-expected) deaths in 2011, 2012, 2013 and 2014 by finding the difference between the observed number of deaths and the number of deaths predicted by the 2001–2010-based time-series models above. 95% confidence intervals were computed using the standard errors of the fitted mortality rate estimates.

We ran separate analyses using the above model for males, females, and both sexes. The age-stratified time-trend analyses examined 10 age groups. The place-specific mortality rates used the same approach but were split into just two age groups, those 60 and over, and those less than 60 in addition to an all-age group analysis.

For the analyses of potential years of life lost, the same process was repeated but using the directly standardized rates of potential years of life lost as the outcome instead of mortality rates.

Life expectancy

A univariate autoregressive integrated moving average (ARIMA) model was fitted to life expectancy data available from 1998 to 2010, and used to predict data points for 2011 and 2012 as per a method by Torri and Vaupel[15]. In order to optimize the p, d, and q parameters, we conducted

a model search and selected the parameterized model that minimized the Akaike Information Criterion (AIC) value, which is defined as:

$$AIC = 2k - 2\ln(L) \quad (3)$$

where k is 3, i.e. the number of parameters in the ARIMA model, and L is the maximized value of the likelihood function for the model. A drift term was included to account for the clear upward trend in life expectancy over the 1998 to 2010 period.

Aggregating results across years

Aggregate central estimates of deaths across time-trend analyses of each projected year were calculated by addition. For 95% confidence intervals, the root-sum-of-squares method was used:

$$d_{agg_{CI}} = d_{agg} \pm \sqrt{\sum_t^n (d_t - d_{t_{CI}})^2} \quad (4)$$

where $d_{agg_{CI}}$ is the aggregate confidence intervals (lower and upper), d_{agg} is the aggregate central estimate, n is the final year in the projected time series, d_t is the central estimate for year t, and $d_{t_{CI}}$ is the confidence interval (lower or upper) for the year t.

Panel-based regression analysis

Model selection

To assess the suitability of different regression approaches, we separately fitted pooled ordinary least squares (OLS), fixed-effects, and random-effects models to the real PEH per capita and population mortality data with sex as the time-invariant entity. The assumption of the random-effects model that the error terms for unobserved or observed time-invariant variables such as sex are uncorrelated with the independent variables means that sex is therefore also able to serve as an independent variable. In fixed-effects models, this is not the case.

We therefore tested whether the consistent but inefficient fixed-effects model was more suitable than the potentially inconsistent but efficient pooled OLS and random-effects approaches by conducting Hausman tests, both of which were highly significant (P<0.001), leading us to reject

the random-effects and pooled OLS approaches and their assumptions in favour of a fixed-effects model.

Regression model

We therefore used the following fixed-effects regression model:

$$H_{it} = \beta_0 + \beta_1 X_{1it} + \ldots + \beta_k X_{kit} + U_{it} \qquad (5)$$

where H_{it} is the response variable for which i is sex and t is time in years; X_{kit} represents the independent variables; β_k is the coefficient for the independent variables; and U_{it} is the error term. For population-based mortality, H was all-cause ASDR. For the regression analyses in which economic and health resource indicators were not controlled, X was just real PEH or real PES per capita in units of ten pounds sterling. To test whether our results were robust to variations in the economy, we added average annual consumer price index (CPI)[16] and unemployment rate[17] as independent variables to the model. For mediation analyses, each health and social care resource was added as an independent variable.[8–12]

Lag analyses

We conducted 1- and 2-year lag analyses. For an *i*th year lag analysis, regression was performed using the mortality rate in year x and the value of the explanatory variables (real PEH or real PES per capita, etc.) in year $x - i$.

Mortality projections to 2020

For the 2020 projection analysis, two projections were performed each with a different observation base: 2001–10 for one and 2009–14 for the other. Data points for 2009 and 2010 were included for the second observation base to give at least six observations from which to make predictions.Instead of comparing actual rates with trend-determined expected rates, the projected rates from[14] using these two different observation bases were compared against each other to calculate rate ratios, and the number of excess deaths annually between 2015 and 2020. Mortality rates for 2015 to 2020 were standardized against the National Population 2012-based projections.[18]

Scenario modelling

For the 2020 projection analysis, we assumed that the mortality projections were underpinned by flat real PEH and PES per capita and no change in efficiency since 2014/15. We performed scenario modeling to test how much of the mortality gap could be closed by planned spending under varying assumptions of annual efficiency changes.

To do this, we first performed fixed-effects regression as before but instead using all-cause population ASDR as the outcome variable, and combined real PEH or PES per capita as the explanatory variable. From these analyses, an increase of £10 per capita real spending on health and social care is associated with 2.56 (2.10 to 3.01) lives saved per 100,000.

To calculate the number of lives saved, *l*, for year *t*, which could be any year between 2015 to 2020, inclusive, we used the following formula:

$$l_t = (1 + p)^{t-2014} \times \frac{r}{100{,}000} \times \frac{(S_t - S_{2014})}{10} \qquad (6)$$

where p is the assumed annual efficiency change; r is the coefficient (or upper or lower bound of the 95% confidence interval); 100,000 is that the coefficient is for all-cause ASDR per 100,000; S_t represents planned health and social care spending combined for year t and S_{2014} denotes the combined health and social care spending outturn for 2014/15. The difference in spending is divided by 10 to acknowledge that the regression coefficient was obtained with units of £10 changes per capita.

The percentage of lives saved was calculated using the number of excess deaths from the mortality projections for year *t*.

The additional spending needed to completely close the gap, i.e. make sure that 100% of lives were saved for year *t*, was computed as follows:

$$S_{additional} = \frac{d_t \times 1{,}000{,}000}{(1+p)^{t-2014} \times r} - S_t + S_{2014} \qquad (7)$$

where d_t is the excess deaths calculated for *t*.

For each year between 2015 and 2020, we modeled three different scenarios, each assuming different values for *p*:

1) a conservative 0% annual efficiency change;
2) a moderate 1% annual efficiency gain (1% was the average efficiency to the nearest integer for the period 2001 to 2013 [2014 data were unavailable]); and
3) an aggressive 3% annual efficiency gain (3% was the highest efficiency to the nearest integer reached in the 2001 to 2013 period.

These productivities were defined by using historical and targeted efficiency changes in healthcare as a guide.[19]

In addition, we asked what annual efficiency gain up to 2020 would be needed if there was no spending on top of the planned PEH and PES budgets. This was calculated as follows:

$$p = \left(\frac{d_{2020} \times 1{,}000{,}000}{r \times (S_{2020} - S_{2014})}\right)^{1/(2020-2014)} - 1 \qquad (8)$$

REFERENCES

1. Office for National Statistics. Mortality Statistics: Metadata. Newport: ONS; 2015.
2. UK Health and Social Care Information Centre. NHS Outcomes Framework Indicators. May 2015. 2015;Available from: http://content.digital.nhs.uk/nhsof
3. HM Treasury. Public Expenditure Statistical Analyses 2015. London: Williams Lea Group; 2015.
4. NHS Digital. Personal Social Services: Expenditure and Unit Costs, England. 2016;Available from: https://www.gov.uk/government/statistics/personal-social-services-expenditure-and-unit-costs-england-2015-to-2016
5. UK Parliament. Impact of the Spending Review on health and social care. 2016;Available from: https://www.publications.parliament.uk/pa/cm201617/cmselect/cmhealth/139/13902.htm?utm_source=139&utm_medium=fullbullet&utm_campaign=Modulereports
6. UK Parliament. The impact of the Spending Review on health and social care finances. 2016;Available from: https://www.publications.parliament.uk/pa/cm201617/cmselect/cmhealth/139/13906.htm
7. UK Department for Communities and Local Government. English Indices of Deprivation. December 2012. 2012;Available from: https://www.gov.uk/government/collections/english-indices-of-deprivation
8. The NHS Information Centre. NHS workforce: summary of staff in the NHS: results from September 2011 Census. 2012;1–31. Available from:

https://catalogue.ic.nhs.uk/publications/workforce/numbers/nhs-staf-2001-2011-medi-dent/nhs-staf-2001-2011-medi-dent-work-rep.pdf

9. Health and Social Care Information Centre. NHS workforce statistics in England, summary of staff in the NHS - 2004-2014. 2015.

10. NHS England. Overnight bed availability and occupancy data. London: 2014.

11. Office for National Statistics. Business Register Employment Survey, 2009-2014. 2014;Available from: https://www.nomisweb.co.uk/

12. Office for National Statistics. Annual Business Enquiry, 2001-2008. 2008;Available from: https://www.nomisweb.co.uk/

13. Office for National Statistics. Correlation between UK SIC 2003 and UK SIC 2007. 2015;Available from: http://webarchive.nationalarchives.gov.uk/20160105160709/http://www.ons.gov.uk/ons/guide-method/classifications/archived-standard-classifications/uk-standard-industrial-classification-1992--sic92-/index.html

14. Dyba T, Hakulinen T, Päivärinta L. A simple non-linear model in incidence prediction. Stat Med 1997;16(20):2297–309.

15. Torri T, Vaupel JW. Forecasting life expectancy in an international context. Int J Forecast 2012;28:519–31.

16. Office for National Statistics. Consumer Price Inflation, release. 2014;Available from: http://webarchive.nationalarchives.gov.uk/20160105160709/http://ons.gov.uk/ons/rel/cpi/consumer-price-indices/december-2014/index.html

17. Office for National Statistics. Unemployment in England, 1992-2015. 2015;https://www.nomisweb.co.uk/reports/lmp/gor/2092957. Available from: https://www.ons.gov.uk/employmentandlabourmarket/peoplenotinwork/unemployment

18. Office for National Statistics. Time series: England population mid-year estimate. 2015;

19. NHS England. NHS Five Year Forward View. Recap briefing for the Health Select Committee on technical modelling and scenarios. 2016.
