# Supplementary material for: Effects of health and social care spending constraints on mortality in England: a time trend analysis
Source: BMJ Open. 2017 Nov 15;7(11):e017722. doi: 10.1136/bmjopen-2017-017722 (PMC5719267; doi:10.1136/bmjopen-2017-017722)
Supplement: Supplementary file 2 [file bmjopen-2017-017722supp002.pdf]

SUPPLEMENTARY FIGURE LEGENDS

Figure S1: Public-sector expenditure on healthcare (PEH) and social care (PES). Data are shown from 2001/02–2014/15. Top plot shows data for PEH whereas bottom plot shows data for PES. Solid lines correspond to total PEH or PES in billions of pounds (left hand *y*-axis). Dotted lines correspond to PEH or PES in pounds per capita (right hand y-axis) calculated from PEH/PES and population size as estimated by the UK's Office for National Statistics (ONS). Blue lines indicate nominal expenditure. Red lines indicate real expenditure adjusted to 2014/15 prices.

Figure S2: Time-trend projections of mortality rates by place of death. Mortality rates (y-axis) per year from 2001 to 2014 are shown for five places of death with 'Other' representing any place of death that doesn't fall into the other four categories (data are unavailable for a breakdown of where these other places might be). The black and blue lines represent actual ASDR for the 2001–10 and 2011–14 periods, respectively. The red line represents predicted ASDR using 2001–10 as an observation base while the 95% CIs are denoted by the beige-colored area.

Figure S3: **Population mortality projections to 2020**. Age-standardised death rates (ASDR) (left hand y-axis) and difference in the number of deaths between actual and predicted rates (right hand y-axis) projected annually to 2020 are shown. Black lines indicate 2009–14 base-fitted (black solid line) and 2015–20 base-forecasted (black dotted line) values. Red lines indicate the same using an observation base for the years 2001–2010. The bisque-coloured area denotes the 95% CIs for the model for 2009–14 data, and the pink area, the 95% CIs for the model for 2001–10 data. The grey bars represent the difference between the number of deaths projected using a 2009–14 observation base and the number forecasted using a 2001–10 observation base, where positive values correspond to excess deaths. Error bars signify 95% CIs. $^*P < 0.05$, $^{**}P < 0.01$ and $^{***}P < 0.001$.

Figure S1

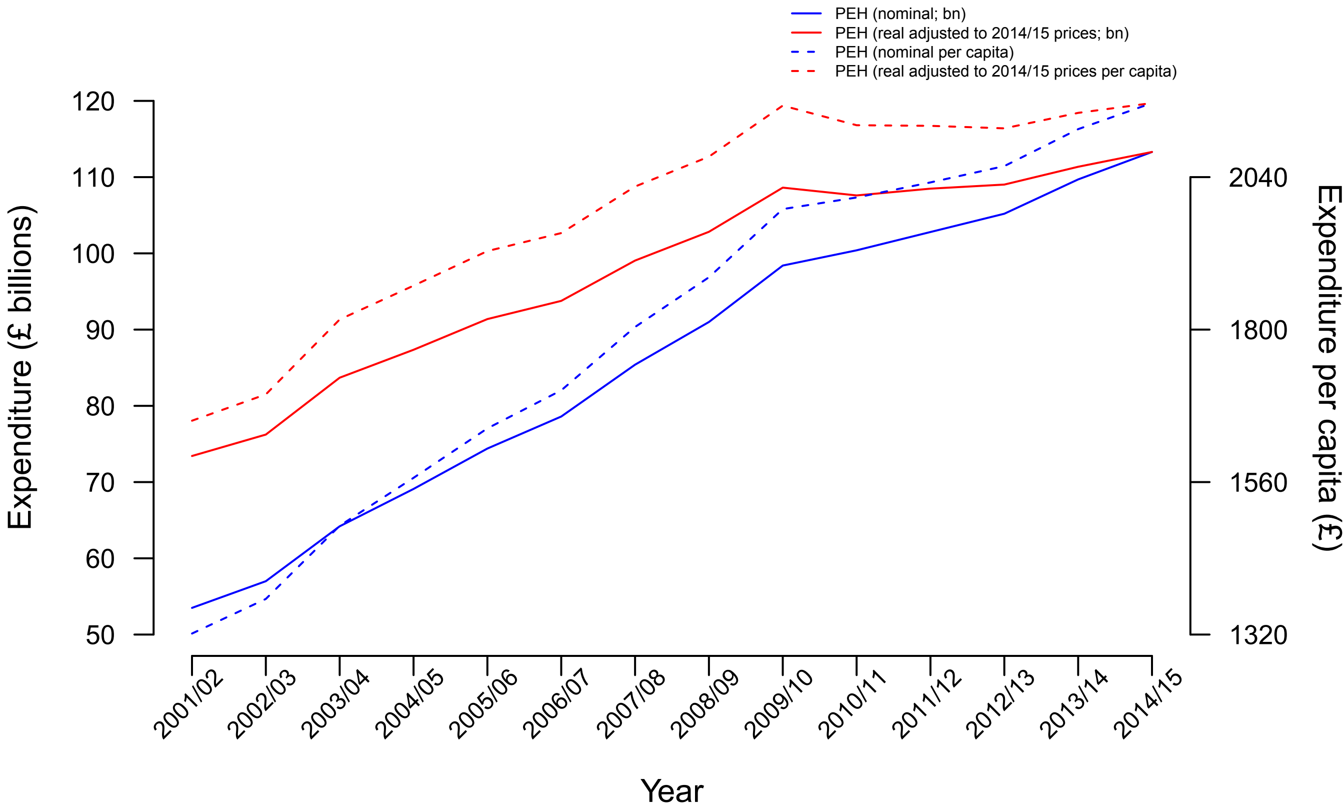
PEH (nominal; bn)
PEH (real adjusted to 2014/15 prices; bn)
PEH (nominal per capita)
PEH (real adjusted to 2014/15 prices per capita)
Expenditure (£ billions)
50 60 70 80 90 100 110 120
Expenditure per capita (£)
1320 1560 1800 2040
2001/02 2002/03 2003/04 2004/05 2005/06 2006/07 2007/08 2008/09 2009/10 2010/11 2011/12 2012/13 2013/14 2014/15
Year

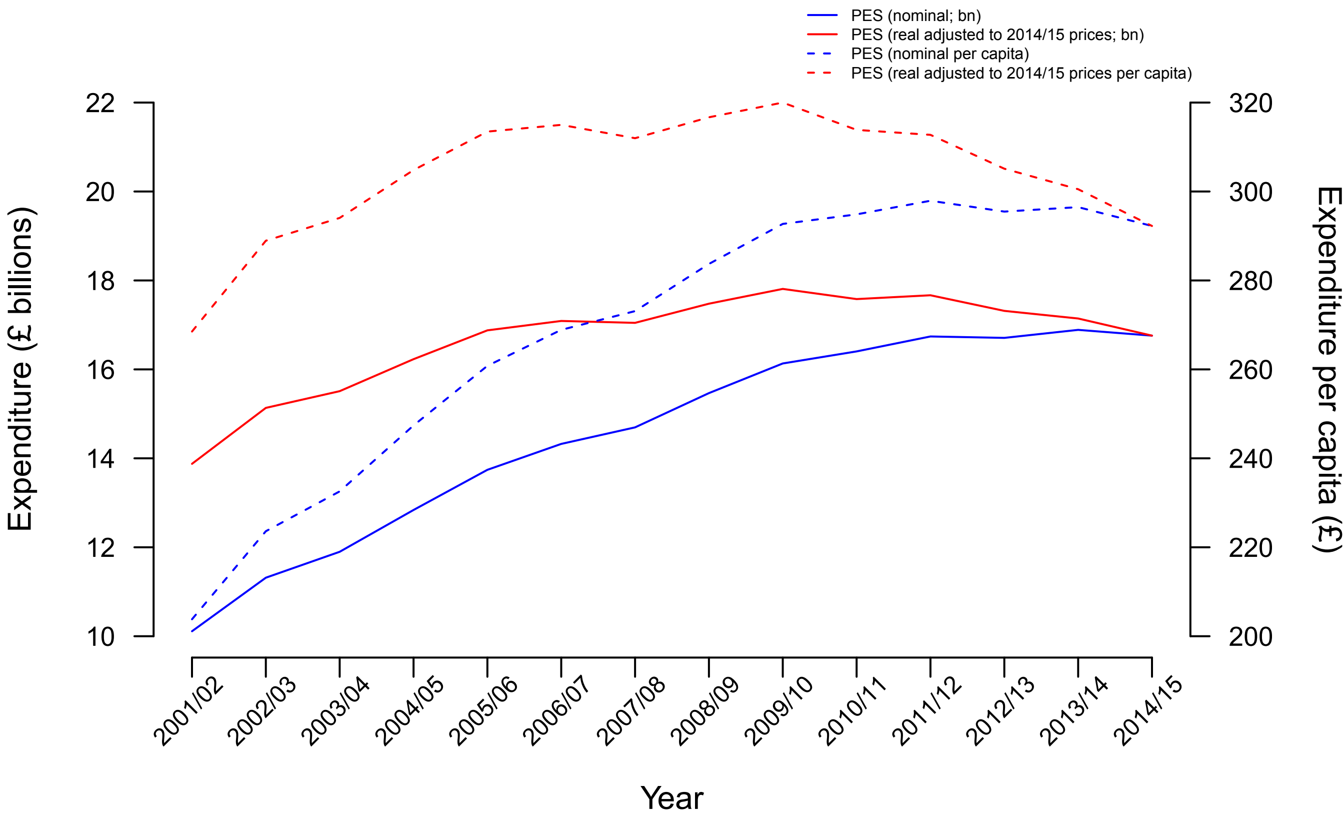
PES (nominal; bn)
PES (real adjusted to 2014/15 prices; bn)
PES (nominal per capita)
PES (real adjusted to 2014/15 prices per capita)
Expenditure (£ billions)
10 12 14 16 18 20 22
Expenditure per capita (£)
200 220 240 260 280 300 320
2001/02 2002/03 2003/04 2004/05 2005/06 2006/07 2007/08 2008/09 2009/10 2010/11 2011/12 2012/13 2013/14 2014/15
Year

Figure S2

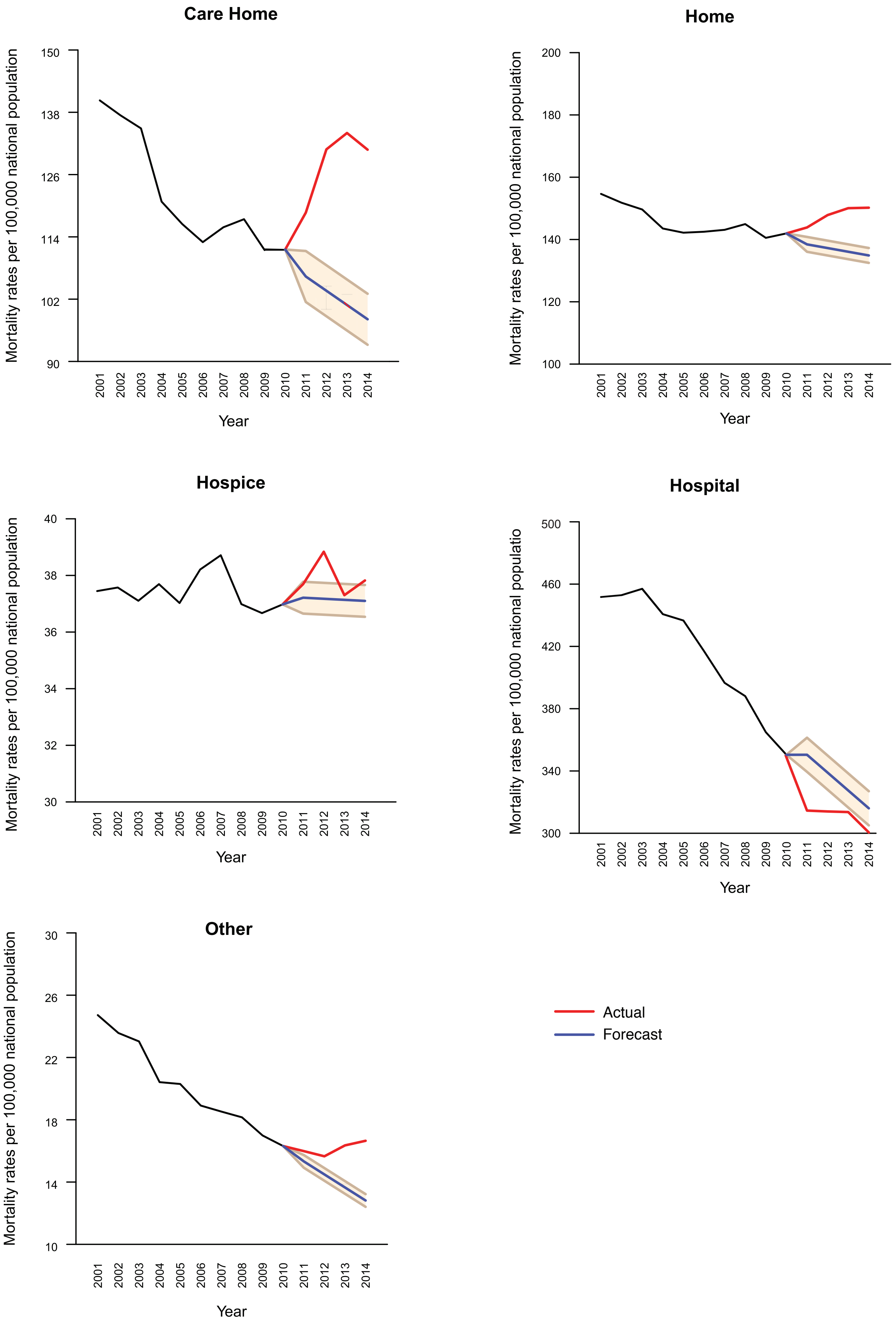

Care Home
Mortality rates per 100,000 national population
150
138
126
114
102
90
2001 2002 2003 2004 2005 2006 2007 2008 2009 2010 2011 2012 2013 2014
Year
Home
Mortality rates per 100,000 national population
200
180
160
140
120
100
2001 2002 2003 2004 2005 2006 2007 2008 2009 2010 2011 2012 2013 2014
Year
Hospice
Mortality rates per 100,000 national population
40
38
36
34
32
30
2001 2002 2003 2004 2005 2006 2007 2008 2009 2010 2011 2012 2013 2014
Year
Hospital
Mortality rates per 100,000 national populatio
500
460
420
380
340
300
2001 2002 2003 2004 2005 2006 2007 2008 2009 2010 2011 2012 2013 2014
Year
Other
Mortality rates per 100,000 national population
30
26
22
18
14
10
2001 2002 2003 2004 2005 2006 2007 2008 2009 2010 2011 2012 2013 2014
Year
Actual
Forecast

Figure S3

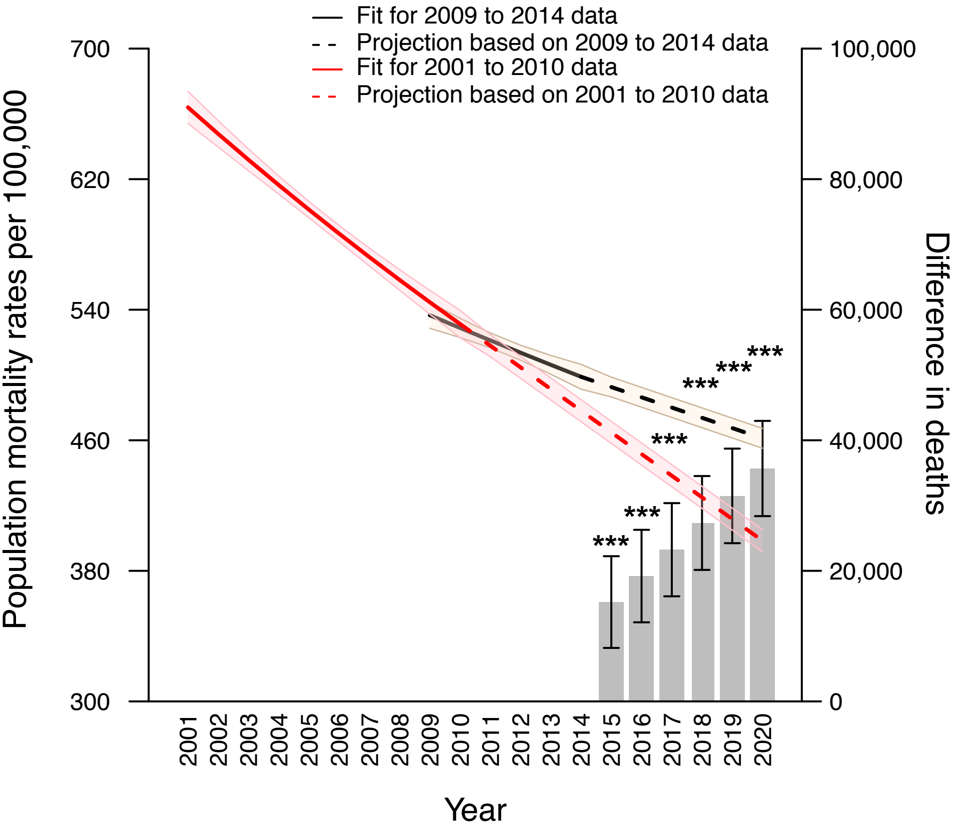

Fit for 2009 to 2014 data
Projection based on 2009 to 2014 data
Fit for 2001 to 2010 data
Projection based on 2001 to 2010 data
Population mortality rates per 100,000
700
620
540
460
380
300
Difference in deaths
100,000
80,000
60,000
40,000
20,000
0
***
2001 2002 2003 2004 2005 2006 2007 2008 2009 2010 2011 2012 2013 2014 2015 2016 2017 2018 2019 2020
Year
