# Supplementary material for: Effects of health and social care spending constraints on mortality in England: a time trend analysis
Source: BMJ Open. 2017 Nov 15;7(11):e017722. doi: 10.1136/bmjopen-2017-017722 (PMC5719267; doi:10.1136/bmjopen-2017-017722)
Supplement: Supplementary file 3 [file bmjopen-2017-017722supp003.pdf]

Table S1. Absolute and relative differences between observed and predicted age-standardised death rates overall and by sex, 2011-2014.

	No. of observed deaths	Difference in deaths (95% CI)	Rate ratio (95% CI)	p-value
**Both men and women**				
2011	452860	-4875.2 (-10850 to 1099.8)	0.99 (0.98 to 1.00)	0.11
2012	466780	8147.8 (2003.6 to 14292)	1.02 (1.00 to 1.03)	0.009
2013	473550	18896 (12641 to 25152)	1.04 (1.03 to 1.06)	<0.0001
2014	468880	18324 (11953 to 24695)	1.04 (1.03 to 1.06)	<0.0001
**Men**				
2011	219070	-1591.2 (-3917.4 to 734.98)	0.99 (0.98 to 1.00)	0.18
2012	224460	3710.4 (1312 to 6108.8)	1.02 (1.01 to 1.03)	0.0003
2013	229290	10166 (7709.7 to 12622)	1.05 (1.03 to 1.06)	<0.0001
2014	229120	11298 (8777.2 to 13819)	1.05 (1.04 to 1.06)	<0.0001
**Women**				
2011	233790	-2888.6 (-6692.4 to 915.16)	0.99 (0.97 to 1.00)	0.14
2012	242320	5343.6 (1444.8 to 9242.3)	1.02 (1.01 to 1.04)	0.01
2013	244260	9530 (5574.5 to 13486)	1.04 (1.02 to 1.06)	<0.0001
2014	239760	8054.3 (4052.6 to 12056)	1.03 (1.02 to 1.05)	<0.0001

Table S2. Predicted and observed life expectancy (years) overall and by sex, 2010-2014.

	Life expectancy (years)		p-value
	**Predicted**	**Observed**	
**2010-2012**			
Male	79.05 (78.98 to 79.12)	79.03	0.71
Female	82.95 (82.86 to 83.04)	82.83	0.08
**2011-2013**			
Male	79.37 (79.27 to 79.47)	79.23	0.009
Female	83.21 (83.08 to 83.33)	82.97	0.0007
**2012-2014**			
Male	79.69 (79.57 to 79.81)	79.37	<0.0001
Female	83.46 (83.31 to 83.62)	83.03	<0.0001

Table S3. Absolute and relative differences between predicted and observed age-standardised PYLL overall and by sex, 2011-2014.

	No. of observed PYLL	Difference in PYLL (95% CI)	Rate ratio (95% CI)	p-value
**Both men and women**				
2011	1110600	5878.8 (214.85 to 11543)	1.01 (1.00 to 1.01)	0.04
2012	1074400	-9359.3 (-15124 to -3594.2)	0.99 (0.99 to 1.00)	0.002
2013	1093200	22568 (16650 to 28485)	1.02 (1.02 to 1.03)	<0.0001
2014	1193000	75496 (69069 to 81923)	1.07 (1.06 to 1.07)	<0.0001
**Men**				
2011	610170	2882.3 (-3093.8 to 8858.5)	1.00 (0.99 to 1.01)	0.35
2012	583320	1794.9 (-4152 to 7741. 8)	1.00 (0.99 to 1.01)	0.57
2013	600650	27893 (21797 to 33989)	1.05 (1.04 to 1.06)	<0.0001
2014	654510	44309 (37538 to 51080)	1.07 (1.06 to 1.08)	<0.0001
**Women**				
2011	500460	3368.2 (487.67 to 6248.7)	1.01 (1.00 to 1.01)	0.02
2012	491090	17219 (14373 to 20064)	1.04 (1.03 to 1.04)	<0.0001
2013	492530	24602 (21688 to 27517)	1.05 (1.05 to 1.06)	<0.0001
2014	538480	32157 (28881 to 35434)	1.06 (1.06 to 1.07)	<0.0001

Table S4. Overall and age group-specific relative differences between observed and predicted death rates, 2011-2014.

	Rate ratio (95% CI)	p-value
**2011**		
All ages (ASR)	0.99 (0.98 to1.00)	0.11
0-34	0.94 (0.92 to 0.96)	<0.0001
35-49	0.96 (0.94 to 0.97)	<0.0001
50-54	0.98 (0.96 to 0.99)	<0.0001
55-59	0.98 (0.97 to 0.99)	0.01
60-64	1.01 (1.01 to 1.02)	0.003
65-69	0.99 (0.98 to 1.00)	0.03
70-74	1.03 (1.02 to 1.05)	<0.0001
75-79	0.99 (0.98 to 1.00)	0.16
80-84	0.96 (0.94 to 0.98)	<0.0001
85 and older	0.99 (0.97 to 1.02)	0.59
**2012**		
All ages (ASR)	1.02 (1.00 to 1.03)	0.01
0-34	0.92 (0.90 to 0.93)	<0.0001
35-49	0.92 (0.91 to 0.93)	<0.0001
50-54	0.96 (0.94 to 0.97)	<0.0001
55-59	0.97 (0.96 to 0.99)	0.0001
60-64	1.04 (1.03 to 1.05)	<0.0001
65-69	1.00 (0.99 to 1.00)	0.32
70-74	1.06 (1.05 to 1.07)	<0.0001
75-79	1.03 (1.02 to 1.04)	<0.0001
80-84	0.99 (0.97 to 1.01)	0.33
85 and older	1.06 (1.03 to 1.08)	<0.0001
**2013**		
All ages (ASR)	1.04 (1.03 to 1.06)	<0.0001
0-34	0.93 (0.91 to 0.94)	<0.0001
35-49	0.94 (0.93 to 0.95)	<0.0001
50-54	0.97 (0.96 to 0.99)	<0.0001
55-59	0.98 (0.96 to 0.99)	<0.0001
60-64	1.08 (1.06 to 1.09)	<0.0001
65-69	1.01 (1.00 to 1.02)	0.02
70-74	1.09 (1.07 to 1.10)	<0.0001
75-79	1.06 (1.05 to 1.08)	<0.0001
80-84	1.01 (0.99 to 1.03)	0.28
85 and older	1.09 (1.06 to 1.11)	<0.0001
**2014**		
All ages (ASR)	1.04 (1.03 to 1.06)	<0.0001
0-34	0.97 (0.95 to 0.99)	0.001
35-49	0.94 (0.93 to 0.95)	<0.0001
50-54	0.98 (0.97 to 1.00)	0.02
55-59	0.97 (0.95 to 0.98)	<0.0001
60-64	1.10 (1.09 to 1.11)	<0.0001
65-69	1.03 (1.02 to 1.04)	<0.0001
70-74	1.11 (1.09 to 1.12)	<0.0001
75-79	1.07 (1.06 to 1.09)	<0.0001
80-84	1.00 (0.98 to 1.02)	0.64
85 and older	1.06 (1.03 to 1.09)	<0.0001

Table S5. Overall and age group-specific relative differences between observed and predicted death rates by place of death, 2011-2014.

	**All ages (ASR)**		**0-59**		**60+**	
	**Rate ratio (95% CI)**	**p-value**	**Rate ratio (95% CI)**	**p-value**	**Rate ratio (95% CI)**	**p-value**
**Care-home**						
2011	1.12 (1.07 to 1.17)	<0.0001	1.03 (1.00 to 1.07)	0.03	1.12 (1.07 to 1.17)	<0.0001
2012	1.26 (1.21 to 1.33)	<0.0001	1.13 (1.10 to 1.17)	<0.0001	1.26 (1.21 to 1.33)	<0.0001
2013	1.33 (1.27 to 1.40)	<0.0001	1.23 (1.19 to 1.27)	<0.0001	1.33 (1.27 to 1.40)	<0.0001
2014	1.33 (1.27 to 1.40)	<0.0001	1.22 (1.18 to 1.26)	<0.0001	1.33 (1.27 to 1.41)	<0.0001
**Home**						
2011	1.04 (1.02 to 1.06)	<0.0001	0.99 (0.98 to 1.01)	0.30	1.05 (1.03 to 1.08)	<0.0001
2012	1.08 (1.06 to 1.10)	<0.0001	0.99 (0.98 to 1.01)	0.26	1.10 (1.08 to 1.13)	<0.0001
2013	1.10 (1.08 to 1.12)	<0.0001	1.03 (1.03 to 1.04)	<0.0003	1.13 (1.11 to 1.15)	<0.0001
2014	1.11 (1.09 to 1.13)	<0.0001	1.04 (1.03 to 1.06)	<0.0001	1.14 (1.11 to 1.16)	<0.0001
**Hospital**						
2011	0.93 (0.90 to 0.96)	<0.0001	0.94 (0.92 to 0.96)	<0.0001	0.93 (0.90 to 0.96)	<0.0001
2012	0.94 (0.91 to 0.97)	0.0001	0.91 (0.89 to 0.93)	<0.0001	0.94 (0.89 to 0.93)	0.0007
2013	0.96 (0.93 to 0.99)	0.01	0.94 (0.92 to 0.96)	<0.0001	0.96 (0.93 to 1.00)	0.03
2014	0.95 (0.92 to 0.99)	0.005	0.96 (0.94 to 0.98)	0.0002	0.95 (0.92 to 0.99)	0.008
**Hospice**						
2011	1.01 (1.00 to 1.03)	0.09	1.01 (0.99 to 1.02)	0.49	1.01 (1.00 to 1.03)	0.04
2012	1.04 (1.00 to 1.03)	<0.0001	1.04 (1.03 to 1.05)	<0.0001	1.04 (1.02 to 1.05)	<0.0001
2013	1.00 (0.99 to 1.02)	0.58	1.00 (0.99 to 1.02)	0.55	1.01 (0.99 to 1.02)	0.29
2014	1.02 (1.00 to 1.03)	0.01	1.01 (1.00 to 1.03)	0.08	1.02 (1.01 to 1.03)	0.006
**Other**						
2011	1.04 (1.02 to 1.07)	0.002	0.96 (0.94 to 0.99)	0.01	1.14 (1.09 to 1.19)	<0.0001
2012	1.08 (1.05 to 1.11)	<0.0001	0.95 (0.92 to 0.98)	0.0003	1.27 (1.21 to 1.33)	<0.0001
2013	1.20 (1.16 to 1.23)	<0.0001	1.02 (0.99 to 1.05)	0.17	1.48 (1.40 to 1.57)	<0.0001
2014	1.30 (1.26 to 1.34)	<0.0001	1.07 (1.04 to 1.10)	<0.0001	1.71 (1.61 to 1.83)	<0.0001

Table S6. Changes in home deaths per 100,000 persons for every £10 change in public expenditure in health (PEH) or social care (PES). Associations were evaluated for up to 2 years of interval between PEH or PES and subsequent home deaths. Number of observations (sex-years) per analysis is 28.

	Lag (year)	PEH per capita (£10)		PES per capita (£10)	
		**β (95% CI)**	**p -value**	**β (95% CI)**	**p-value**
**Model 1**	0	-0.13 (-0.21 to -0.05)	0.005	-2.63 (-3.27 to -1.99)	<0.0001
	1	-0.07 (-0.15 to 0.02)	0.13	-1.78 (-2.54 to -1.01)	0.0002
	2	0.005 (-0.07 to 0.08)	0.90	-0.71 (-1.58 to 0.16)	0.12
**Model 2**	0	-0.31 (-0.39 to -0.22)	<0.0001	-2.65 (-3.30 to -2.01)	<0.0001
	1	-0.25 (-0.33 to -0.15)	<0.0001	-2.05 (-2.69 to -1.40)	<0.0001
	2	-0.20 (-0.29 to -0.11)	0.0002	-1.22 (-1.93 to -0.52)	0.003
**Model 3**	0	-0.41 (-0.53 to -0.29)	<0.0001	-2.68 (-3.40 to -1.96)	<0.0001
	1	-0.36 (-0.46 to -0.26)	<0.0001	-2.32 (-3.09 to -1.54)	<0.0001
	2	-0.25 (-0.35 to -0.14)	0.0002	-1.17 (-2.56 to -0.88)	0.0008
**Model 4**	0	0.02 (-0.05 to 0.09)	0.62	-2.78 (-3.65 to -1.91)	<0.0001
	1	0.12 (0.03 to 0.21)	0.01	-2.96 (-4.06 to -1.86)	<0.0001
	2	0.21 (0.09 to 0.32)	0.002	-2.79 (-4.11 to -1.47)	0.0005

Model 1: Unadjusted model
Model 2: Adjusted for basic state pension per week
Model 3: Adjusted for unemployment rate and CPI
Model 4: PEH and PES were included in the same model

Table S7. Changes in hospital deaths per 100,000 persons for every £10 change in public expenditure in health (PEH) or social care (PES). Associations were evaluated for up to 2 years of interval between PEH or PES and subsequent hospital deaths. Number of observations (sex-years) per analysis is 28.

	Lag (year)	PEH per capita (£10)		PES per capita (£10)	
		**β (95% CI)**	**p -value**	**β (95% CI)**	**p-value**
**Model 1**	0	-2.60 (-3.11 to -2.08)	<0.0001	-11.13 (-2.62 to 2.35)	0.12
	1	-2.70 (-3.11 to -2.29)	<0.0001	-19.92 (-31.80 to -8.04)	0.003
	2	-2.66 (-2.30 to -2.35)	<0.0001	-24.02 (-33.30 to -14.74)	<0.0001
**Model 2**	0	-1.36 (-1.79 to -0.94)	<0.0001	-9.11 (-13.57 to -4.65)	0.0005
	1	-1.66 (-1.94 to -1.38)	<0.0001	-12.69 (-15.96 to -9.42)	0.0002
	2	-1.78 (-2.07 to -1.49)	<0.0001	-13.21 (-15.49 to -10.94)	<0.0001
**Model 3**	0	-0.33 (-0.76 to 0.11)	0.16	-1.39 (-4.24 to 1.46)	0.35
	1	-0.07 (-0.12 to -0.03)	0.003	-6.37 (-9.14 to -3.60)	0.01
	2	-0.11 (-0.16 to -0.05)	0.001	-10.14 (-12.81 to -7.48)	0.0006
**Model 4**	0	-3.56 (-3.96 to -3.15)	<0.0001	17.98 (12.95 to 23.02)	<0.0001
	1	-3.69 (-4.11 to -3.27)	<0.0001	15.49 (10.31 to 20.66)	<0.0001
	2	-3.36 (-3.84 to -2.89)	<0.0001	9.73 (4.11 to 15.35)	0.003

Model 1: Unadjusted model
Model 2: Adjusted for basic state pension per week
Model 3: Adjusted for unemployment rate and CPI
Model 4: PEH and PES were included in the same model

Table S8. Changes in home deaths per 100,000 persons associated with changes in public expenditure in social care (PES), adjusted for quantity of resources. Associations were evaluated for up to 2 years of interval between real PES per capita and subsequent home deaths. Number of observations (sex-years) per analysis is 28.

Resource	Lag (year)	Home deaths per 100,000 persons			
		PES per capita (£10)		Resource quantity (thousands)[1]	
		β (95% CI)	p-value	β (95% CI)	p-value
No. of hospital doctors	0	-2.76 (-3.53 to -1.98)	<0.0001	0.03 (-0.07 to 0.12)	0.57
	1	-2.86 (-3.76 to -1.97)	<0.0001	0.20 (-0.08 to 0.31)	0.003
	2	-2.74 (-3.72 to -1.76)	<0.0001	0.35 (0.21 to 0.49)	<0.0001
No. of GPs	0	-2.73 (-3.52 to -1.95)	<0.0001	0.10 (-0.32 to 0.52)	0.0005
	1	-2.76 (-3.68 to -1.84)	<0.0001	0.78 (-0.28 to 1.28)	0.001
	2	-2.51 (-3.47 to -1.55)	<0.0001	1.34 (0.79 to 1.90)	0.002
No. of nurses	0	-2.65 (-3.51 to -1.79)	<0.0001	0.002 (-0.08 to 0.09)	0.96
	1	-0.92 (-1.79 to -0.05)	0.05	-0.13 (-0.21 to -0.04)	0.007
	2	0.45 (-0.40 to 1.30)	0.31	-0.17 (-0.26 to -0.09)	0.0005
No. of scientific, therapeutic and technical staff	0	-2.76 (-3.57 to -1.96)	<0.0001	0.02 (-0.06 to 0.11)	0.60
	1	-2.91 (-3.86 to -1.97)	<0.0001	0.18 (0.07 to 0.28)	0.004
	2	-2.80 (-3.82 to -1.78)	<0.0001	0.31 (0.18 to 0.43)	<0.0001
No. of ambulance staff	0	-2.69 (-3.49 to -1.90)	<0.0001	0.14 (-0.87 to 1.16)	0.79
	1	-2.60 (-3.54 to -1.66)	<0.0001	1.58 (0.36 to 2.80)	0.02
	2	-2.09 (-3.18 to -0.99)	0.001	2.49 (0.99 to 3.99)	0.004
No. of clinical support staff	0	-2.53 (-3.30 to -1.77)	<0.0001	-0.03 (-0.13 to 0.08)	0.63
	1	-1.11 (-1.97 to -0.26)	0.02	-0.15 (-0.27 to -0.04)	0.02
	2	-0.10 (-1.10 to 0.90)	0.85	-0.14 (-0.28 to -0.005)	0.05
No. of infrastructure support staff	0	-2.24 (-3.51 to -0.97)	0.002	-0.05 (-0.19 to 0.09)	0.49
	1	-2.01 (-3.71 to -0.31)	0.03	0.03 (-0.15 to 0.21)	0.76
	2	-2.46 (-4.21 to -0.71)	0.01	0.21 (0.02 to 0.40)	0.04
No. of overnight beds	0	-2.71 (-3.41 to -2.02)	<0.0001	-0.02 (-0.08 to 0.04)	0.522
	1	-2.44 (-3.14 to -1.75)	<0.0001	-0.12 (-0.18 to -0.06)	0.0009
	2	-1.79 (-2.52 to -1.07)	<0.0001	-0.18 (-0.25 to -0.11)	<0.0001
No. of social care staff with accommodation	0	-2.63 (-3.29 to -1.97)	<0.0001	-0.003 (-0.07 to 0.06)	0.94
	1	-2.03 (-2.76 to -1.31)	<0.0001	0.10 (0.03 to 0.18)	0.02
	2	-1.05 (-1.88 to -0.22)	0.02	0.12 (0.02 to 0.21)	0.0002
No. of social care staff without accommodation	0	-2.71 (-3.42 to -1.99)	<0.0001	0.002 (-0.007 to 0.01)	0.61
	1	-2.43 (-3.20 to -1.65)	<0.0001	0.02 (0.006 to 0.03)	0.006
	2	-1.87 (-2.66 to -1.08)	0.0002	0.03 (0.02 to 0.04)	0.0002

[1]Estimates are shown for each corresponding resource in the left-hand column.

Table S9. Changes in care-home deaths per 100,000 persons associated with changes in public expenditure in healthcare (PEH), adjusted for quantity of resources. Associations were evaluated for up to 2 years of interval between real PES per capita and subsequent care-home deaths. Number of observations (sex-years) per analysis is 28.

Resource	Lag (year)	Care-home deaths per 100,000 persons			
		PES per capita (£10)		Resource quantity (thousands)[1]	
		β (95% CI)	p-value	β (95% CI)	p-value
No. of hospital doctors	0	-1.59 (-2.30 to -0.87)	0.0002	2.17 (1.08 to 3.26)	0.003
	1	-1.72 (-2.43 to -1.01)	<0.0001	2.62 (1.51 to 3.73)	0.002
	2	-1.39 (-2.20 to -0.59)	0.003	2.41 (1.11 to 3.71)	0.001
No. of GPs	0	-1.48 (-2.28 to -0.68)	0.001	8.69 (3.41 to 13.97)	0.01
	1	-1.64 (-2.35 to -0.94)	0.0002	10.66 (5.92 to 15.41)	0.0002
	2	-1.37 (-2.05 to -0.69)	0.0008	9.96 (5.35 to 14.57)	0.0004
No. of nurses	0	-0.15 (-0.30 to -0.002)	0.07	-0.35 (-0.54 to -0.17)	0.001
	1	-0.01 (-0.04 to 0.11)	0.83	-0.45 (-0.59 to -0.31)	<0.0001
	2	0.16 (0.04 to 0.28)	0.02	-0.42 (-0.56 to -0.28)	<0.0001
No. of scientific, therapeutic and technical staff	0	-1.53 (-2.42 to -0.64)	0.003	1.87 (0.64 to 3.09)	0.006
	1	-1.58 (-2.45 to -0.71)	0.002	2.12 (0.91 to 3.32)	0.002
	2	-1.30 (-2.15 to -0.45)	0.007	1.96 (0.76 to 3.16)	0.004
No. of ambulance staff	0	-0.37 (-0.78 to 0.04)	0.09	3.17 (-3.39 to 9.72)	0.35
	1	-0.24 (-0.65 to 0.18)	0.28	2.69 (-3.90 to 9.29)	0.43
	2	-0.07 (-0.46 to 0.33)	0.74	2.39 (-3.93 to 8.71)	0.47
No. of clinical support staff	0	-0.15 (-0.33 to 0.02)	0.10	-0.33 (-0.63 to -0.03)	0.04
	1	-0.02 (-0.17 to 0.11)	0.69	-0.55 (-0.78 to -0.31)	0.0002
	2	0.12 (-0.04 to 0.28)	0.16	-0.43 (-0.68 to -0.18)	0.003
No. of infrastructure support staff	0	0.25 (0.004 to 0.50)	0.06	-0.74 (-1.08 to -0.41)	0.0002
	1	0.38 (0.08 to 0.68)	0.02	-0.72 (-1.11 to -0.32)	0.002
	2	0.33 (-0.09 to 0.74)	0.14	-0.37 (-0.89 to 0.15)	0.18
No. of overnight beds	0	-0.84 (-1.12 to -0.52)	<0.0001	-0.76 (-1.09 to -0.42)	0.0002
	1	-0.84 (-1.08 to -0.59)	<0.0001	-0.92 (-1.19 to -0.65)	<0.0001
	2	-0.60 (-0.85 to -0.35)	0.0001	-0.86 (-1.15 to -0.57)	<0.0001
No. of social care staff with accommodation	0	-0.29 (-0.50 to -0.08)	0.01	0.21 (-0.04 to 0.46)	0.11
	1	-0.15 (-0.36 to -0.06)	0.18	0.19 (-0.09 to 0.47)	0.20
	2	-0.02 (-0.18 to -0.23)	0.83	0.13 (-0.14 to 0.41)	0.35
No. of social care staff without accommodation	0	-0.96 (-1.34 to -0.58)	<0.0001	0.13 (0.07 to 0.20)	0.0003
	1	-0.96 (-1.28 to -0.64)	0.0003	0.16 (0.10 to 0.21)	<0.0001
	2	-0.79 (-1.06 to -0.52)	0.0003	0.16 (0.11 to 0.21)	<0.0001

[1]Estimates are shown for each corresponding resource in the left-hand column.

Table S10. Changes in home deaths per 100,000 persons associated with changes in public expenditure in healthcare (PEH), adjusted for quantity of resources. Associations were evaluated for up to 2 years of interval between real PES per capita and subsequent home deaths. Number of observations (sex-years) per analysis is 28.

Resource	Lag (year)	Home deaths per 100,000 persons			
		PES per capita (£10)		Resource quantity (thousands) [1]	
		β (95% CI)	p-value	β (95% CI)	p-value
No. of hospital doctors	0	-0.70 (-1.05 to -0.36)	0.0005	0.89 (0.36 to 1.42)	0.003
	1	-0.75 (-1.06 to -0.44)	0.0001	1.09 (0.61 to 1.58)	0.0002
	2	-0.67 (-0.97 to -0.37)	0.0003	1.11 (0.62 to 1.61)	0.0003
No. of GPs	0	-0.61 (-1.00 to -0.22)	0.005	3.26 (0.69 to 5.83)	0.02
	1	-0.65 (-0.98 to -0.32)	0.0009	4.00 (1.76 to 6.23)	0.002
	2	-0.62 (-0.89 to -0.36)	0.0002	4.37 (2.57 to 6.16)	0.0001
No. of nurses	0	-0.11 (-0.18 to -0.04)	0.006	-0.15 (-0.23 to -0.06)	0.004
	1	-0.03 (-0.10 to 0.02)	0.22	-0.18 (-0.25 to -0.11)	<0.0001
	2	0.04 (-0.01 to 0.10)	0.16	-0.16 (-0.22 to -0.10)	<0.0001
No. of scientific, therapeutic and technical staff	0	-0.59 (-1.02 to -0.15)	0.01	0.64 (0.03 to 1.24)	0.05
	1	-0.64 (-1.03 to -0.25)	0.004	0.81 (0.27 to 1.36)	0.008
	2	-0.59 (-0.93 to -0.25)	0.003	0.86 (0.38 to 1.34)	0.001
No. of ambulance staff	0	-0.14 (-0.34 to 0.05)	0.15	0.25 (-2.79 to 3.29)	0.87
	1	-0.12 (-0.30 to -0.05)	0.19	1.05 (-1.79 to 3.88)	0.48
	2	-0.06 (-0.22 to 0.11)	0.50	1.13 (-1.48 to 3.73)	0.41
No. of clinical support staff	0	-0.11 (-0.19 to -0.03)	0.01	-0.17 (-0.30 to -0.04)	0.02
	1	-0.04 (-0.11 to 0.02)	0.20	-0.23 (-0.33 to -0.12)	0.0003
	2	0.03 (-0.04 to 0.10)	0.48	-0.16 (-0.27 to -0.05)	0.01
No. of infrastructure support staff	0	0.08 (-0.02 to 0.19)	0.14	-0.36 (-0.50 to -0.21)	<0.0001
	1	0.15 (0.02 to 0.27)	0.03	-0.32 (-0.49 to -0.16)	0.0007
	2	0.11 (-0.06 to 0.28)	0.22	-0.15 (-0.36 to 0.07)	0.19
No. of overnight beds	0	-0.40 (-0.55 to -0.25)	<0.0001	-0.32 (-0.48 to -0.16)	0.0007
	1	-0.38 (-0.49 to -0.27)	<0.0001	-0.38 (-0.50 to -0.26)	<0.0001
	2	-0.26 (-0.37 to -0.15)	0.0002	-0.34 (-0.47 to -0.21)	<0.0001
No. of social care staff with accommodation	0	-0.16 (-0.26 to -0.06)	0.004	0.07 (-0.05 to 0.19)	0.25
	1	-0.10 (-0.18 to -0.01)	0.04	0.10 (-0.02 to 0.22)	0.11
	2	-0.02 (-0.10 to 0.06)	0.61	0.09 (-0.02 to 0.20)	0.13
No. of social care staff without accommodation	0	-0.45 (-0.63 to -0.27)	<0.0001	0.06 (0.03 to 0.09)	0.001
	1	-0.40 (-0.56 to -0.25)	<0.0001	0.06 (0.04 to 0.09)	0.0001
	2	-0.31 (-0.45 to -0.18)	0.0001	0.06 (0.04 to 0.08)	<0.0001

[1]Estimates are shown for each corresponding resource in the left-hand column.

Table S11. Changes in care-home and home deaths per 100,000 persons associated with change in no. of nurses. Associations were evaluated for up to 2 years of interval between no. of nurses and subsequent home deaths. Number of observations (sex-years) per analysis is 28.

**Place of deaths**	**Lag (year)**	**No. of nurses (thousands)**	
		**β (95% CI)**	**p-value**
Care-home deaths per 100,000 persons	0	-0.38 (-0.57 to -0.19)	0.0007
	1	-0.46 (-0.59 to -0.32)	<0.0001
	2	-0.37 (-0.52 to -0.22)	0.0001
Home deaths per 100,000 persons	0	-0.17 (-0.27 to -0.07)	0.003
	1	-0.18 (-0.25 to -0.12)	<0.0001
	2	-0.15 (-0.21 to -0.08)	0.0002

Table S12. Projected excess deaths prevented in 2015-20 by planned health and social spending under different scenarios of annual productivity increases along with the corresponding additional spending needed to completely close the mortality gap. Numbers in parentheses are 95% confidence intervals.

Scenario	Annual productivity (%)	Excess deaths prevented (%)	Additional spending to prevent 100% of excess deaths (£bn)
**2015**			
Conservative	0	23.58 (19.39 to 27.79)	4.548 (3.647 to 5.84)
Moderate	1	23.82 (19.58 to 28.06)	4.49 (3.6 to 5.76)
Aggressive	3	24.29 (19.96 to 28.62)	4.37 (3.5 to 5.63)
**2016**			
Conservative	0	45.3 (37.24 to 53.39)	4.11 (2.97 to 5.73)
Moderate	1	46.21 (37.99 to 54.48)	3.96 (2.85 to 5.55)
Aggressive	3	48.06 (39.51 to 56.61)	3.68 (2.61 to 5.21)
**2017**			
Conservative	0	51.56 (42.4 to 60.77)	4.4 (3.03 to 6.37)
Moderate	1	53.15 (43.68 to 62.62)	4.13 (2.8 to 6.04)
Aggressive	3	56.38 (46.35 to 66.4)	3.63 (2.37 to 5.43)
**2018**			
Conservative	0	53.56 (44.01 to 63.11)	4.96 (3.35 to 7.28)
Moderate	1	55.72 (45.8 to 65.67)	4.55 (2.99 to 6.77)
Aggressive	3	60.29 (49.54 to 71.05)	3.77 (2.34 to 5.83)
**2019**			
Conservative	0	58.33 (47.94 to 68.71)	5.13 (3.27 to 7.8)
Moderate	1	61.28 (50.38 to 72.24)	4.53 (2.76 to 7.07)
Aggressive	3	67.6 (55.56 to 79.67)	3.44 (1.83 to 5.74)
**2020**			
Conservative	0	54.1 (44.46 to 63.74)	6.41 (4.29 to 9.43)
Moderate	1	57.41 (47.2 to 67.67)	5.6 (3.61 to 8.44)
Aggressive	3	64.58 (53.09 to 76.1)	4.14 (2.37 to 6.67)
Ideal	10.79 (7.8 to 14.46)	100	0

SUPPLEMENTARY FIGURE LEGENDS

Figure S1: Public-sector expenditure on healthcare (PEH) and social care (PES). Data are shown from 2001/02–2014/15. Top plot shows data for PEH whereas bottom plot shows data for PES. Solid lines correspond to total PEH or PES in billions of pounds (left hand *y*-axis). Dotted lines correspond to PEH or PES in pounds per capita (right hand y-axis) calculated from PEH/PES and population size as estimated by the UK's Office for National Statistics (ONS). Blue lines indicate nominal expenditure. Red lines indicate real expenditure adjusted to 2014/15 prices.

Figure S2: Time-trend projections of mortality rates by place of death. Mortality rates (y-axis) per year from 2001 to 2014 are shown for five places of death with 'Other' representing any place of death that doesn't fall into the other four categories (data are unavailable for a breakdown of where these other places might be). The black and blue lines represent actual ASDR for the 2001–10 and 2011–14 periods, respectively. The red line represents predicted ASDR using 2001–10 as an observation base while the 95% CIs are denoted by the beige-colored area.

Figure S3: **Population mortality projections to 2020**. Age-standardised death rates (ASDR) (left hand y-axis) and difference in the number of deaths between actual and predicted rates (right hand y-axis) projected annually to 2020 are shown. Black lines indicate 2009–14 base-fitted (black solid line) and 2015–20 base-forecasted (black dotted line) values. Red lines indicate the same using an observation base for the years 2001–2010. The bisque-coloured area denotes the 95% CIs for the model for 2009–14 data, and the pink area, the 95% CIs for the model for 2001–10 data. The grey bars represent the difference between the number of deaths projected using a 2009–14 observation base and the number forecasted using a 2001–10 observation base, where positive values correspond to excess deaths. Error bars signify 95% CIs. *$P < 0.05$, **$P < 0.01$ and ***$P < 0.001$.
