# Supplementary material for: Effects of health and social care spending constraints on mortality in England: a time trend analysis
Source: BMJ Open. 2017 Nov 15;7(11):e017722. doi: 10.1136/bmjopen-2017-017722 (PMC5719267; doi:10.1136/bmjopen-2017-017722)
Supplement: Author's manuscript [file bmjopen-2017-017722.draft_revisions.pdf]

BMJ Open is committed to open peer review. As part of this commitment we make the peer review history of every article we publish publicly available.

When an article is published we post the peer reviewers' comments and the authors' responses online. We also post the versions of the paper that were used during peer review. These are the versions that the peer review comments apply to.

The versions of the paper that follow are the versions that were submitted during the peer review process. They are not the versions of record or the final published versions. They should not be cited or distributed as the published version of this manuscript.

BMJ Open is an open access journal and the full, final, typeset and author-corrected version of record of the manuscript is available on our site with no access controls, subscription charges or pay-per-view fees (http://bmjopen.bmj.com).

If you have any questions on BMJ Open's open peer review process please email editorial.bmjopen@bmj.com

BMJ Open

Effects of health and social care spending constraints on mortality in England

Journal:	*BMJ Open*
Manuscript ID	bmjopen-2017-017722
Article Type:	Research
Date Submitted by the Author:	12-May-2017
Complete List of Authors:	Watkins, Johnathan; PILAR Research and Education<br>Wulaningsih, Wahyu<br>Da Zhou, Charlie<br>Marshall, Dominic<br>Sylianteng, Guia<br>Dela Rosa, Phyllis<br>Miguel, Viveka<br>Raine, Rosalind<br>King, Lawrence; Cambridge University<br>Maruthappu, Mahiben
**Primary Subject Heading**:	Health economics
Secondary Subject Heading:	Epidemiology, Health policy, Health services research, Public health
Keywords:	HEALTH ECONOMICS, expenditure, mortality, social care, life expectancy

SCHOLARONE™ Manuscripts

Effects of health and social care spending constraints on mortality in England

Johnathan Watkins[1,2]*†, Wahyu Wulaningsih[2,3]*, Charlie Da Zhou[4], Dominic C. Marshall[5], Guia D.C Sylianteng[2,6], Phyllis G. Dela Rosa[2,7], Viveka A. Miguel[2,8], Rosalind Raine[9], Lawrence P. King[10], Mahiben Maruthappu[9†]

[1] Institute for Mathematical & Molecular Biomedicine, King's College London, London, UK

[2] PILAR Research and Education, Cambridge, UK

[3] MRC Unit for Lifelong Health and Ageing, University College London, London, UK

[4] Medical Sciences Division, University of Oxford, Oxford, UK

[5] Oxford University Clinical Academic Graduate School, John Radcliffe Hospital, Oxford, UK

[6] London School of Hygiene and Tropical Medicine, London, UK

[7] University of the Philippines Manila, Metro Manila, Philippines

[8] University of the Philippines Diliman, Quezon City, Metro Manila, Philippines

[9] Department of Applied Health Research, University College London, London, UK

[10] Department of Sociology, University of Cambridge, Cambridge, UK

*** These authors contributed equally**

† Corresponding authors:
Dr. Mahiben Maruthappu
Department of Applied Health Research,
1–19 Torrington Place, University College London, WC1E 7HB, UK
Email: m.maruthappu@gmail.com

Dr. Johnathan Watkins
PILAR Research and Education
20 Station Road, Cambridge, CB1 2JD, UK
Email: jwatkins@pilar.org.uk

Word count: abstract (250), manuscript (2,851)

Number of references: 22

Short title: Health and social care spending and mortality

For peer review only - http://bmjopen.bmj.com/site/about/guidelines.xhtml

For peer review only

ABSTRACT

Objective

In 2010, England experienced constraints in public expenditure on healthcare (PEH) and social care (PES). We sought whether these constraints affected subsequent mortality rates.

Methods

We collected data on health and social care resources and finances for England from 2001 to 2014. Time-trend analyses were conducted to compare the actual mortality rates in 2011–14 with the counterfactual rates expected based on trends before the spending constraints. Fixed-effects regression analyses using PES, PEH, resource data, and mortality were also performed.

Results

The 2010–14 spending constraints were associated with an estimated 45,368 (95% confidence interval (CI): 34,530–56,206) higher-than-expected number of deaths. Deaths in those aged ≥60 and in care homes accounted for the majority. PES was more strongly linked with care-home and home mortality than PEH, with each £10 per capita decline in real PES associated with an increase of 5.10 (3.65–6.54) ($P<0.001$) care-home deaths per 100,000. These associations persisted in lag analyses and after adjustment for macroeconomic forces. Furthermore, we found that real PES per capita may influence mortality primarily via changes in nurse numbers. Projections to 2020 suggested that planned health and social care spending would not close the gap and is cumulatively linked to an estimated 152,141 (95% CI: 134,597 and 169,685) additional deaths.

For peer review only - http://bmjopen.bmj.com/site/about/guidelines.xhtml

Conclusions

Spending constraints, especially PES, may have produced a substantial mortality gap. We suggest that spending should be targeted on improving care delivered in care homes and at home; and maintaining or increasing nurse numbers.

KEYWORDS

Health care, social care, spending, expenditure, mortality, PYLL, life expectancy, time trend

STRENGTHS AND LIMITATIONS

- Time trend analysis with projections of future outcomes and counterfactual analysis
- Sensitivity analyses by using other outcomes: potential years of life lost and life expectancy
- Analysis included potential effect modifiers and mediating factors
- Results only imply association rather than causation
- There might be other mediating factors beyond those identified in this study

For peer review only

INTRODUCTION

Health systems in most industrialised countries are facing the concurrent challenge of managing both rising demand and funding constraints following the recent global economic crisis. The single-payer National Health Service (NHS) in England, which provides tax-funded, universal health coverage, is no exception.[1–4] Since 2010, the NHS in England has seen a real-term annual increase in public healthcare spending in England of 1.30% between 2010 and 2014, as compared to historical annual growth of around 4%.[4] During the same period, demand and healthcare cost inflation increased sharply, with medical advances and a growing and ageing population contributing. By 2020/21, a funding gap, between what is needed and what is available, has been predicted, unless major changes are implemented [4]

Public-sector funding for social care in England has also suffered.[5] Such funds enable the provision of means-tested home care and care-home accommodation,[6] allowing, for example, hospitals to discharge frail patients who would otherwise have no adequate support. Real-term adult social care spending decreased by 1.19% annually between 2010 and 2014, reversing the annual increase of 3.17% between 2001 and 2009. This is despite increasing demand, with the group most likely to require social care – the over-85s – set to rise from 1.6 million in 2015 to 1.8 million in 2020.[7]

This supply-demand mismatch has manifested in several ways. During the first week of 2017, more than four in 10 NHS hospitals declared a major alert.[8] Emergency medicine departments (A&E) saw 900,000 (4.6%) more attendances in 2015/16 compared with the previous year, and 4% more emergency hospital admissions.[9] Over the past two years, the number of elderly patients waiting

For peer review only - http://bmjopen.bmj.com/site/about/guidelines.xhtml

over 12 hours in A&E has trebled, and there has been a 31% increase in delayed hospital discharges. [9]

While the funding gaps facing health and social care have been well-quantified,[10,11] the impact on population outcomes remains unclear. Here we sought to model the past, present, and future impact of funding constraints experienced by the public health and social care system in England on mortality, to provide insights into the association between funding and health outcomes and inform future financial allocations.

METHODS

Data collection

Population mortality data for England were extracted from the UK's Office for National Statistics (ONS)[12] based on Medical Certificates of Cause of Death from the Registration Online system. Age-standardized death rates (ASDR) were calculated with reference to a standard European population using mortality data split into ten age groups (see Supplementary Appendix). Information on deaths by place of occurrence (care homes, hospice, home, hospital, and other establishments) was provided by Public Health England. Additionally, mortality data were collected for 325 lower-tier local authorities (LAs) based on 2010 boundaries with Cornwall and the Isles of Scilly combined, due to the small population of the latter. Data on life expectancy were obtained from the ONS, whereas data on potential years of life lost (PYLL), a measure of premature mortality from causes considered amenable to healthcare, were obtained from the UK Health and

For peer review only - http://bmjopen.bmj.com/site/about/guidelines.xhtml

Social Care Information Centre[13] and age-standardized. Further details, including health and social care resource data (staff and bed numbers), are provided in the Supplementary Appendix.

Statistical analysis

For all analyses, we used 2001 as the start of the study period since complete population mortality data were available from this year onwards, as well as it being first year that ICD-10 was used. Time-trend analyses were conducted using Poisson or quasi-Poisson regression models with either all-cause ASDR or rates of PYLL as the dependent variable and calendar year as the independent variable. Analyses were repeated for each sex, age group, place of death, and local government area. Further details are provided in the Supplementary Appendix.

Fixed-effects regression models were conducted using real PEH/PES per capita and controls for economic variation (unemployment[14] and the average annual consumer price index [CPI][15]) and average weekly pensions as the independent variables, and care home, home and hospital mortality as the dependent variables. These were repeated for 1- and 2-year lag periods.

We explored potential mediating factors using the resource data by running fixed-effects regression models with real PEH/PES per capita and each resource variable as the independent variables; and care home/home mortality as the dependent variable. Resource variables that were associated with the dependent variable, resulted in weaker associations between PEH/PES and the dependent variable, and retained the same coefficient sign as univariable regressions were considered to be putative mediators.

For peer review only - http://bmjopen.bmj.com/site/about/guidelines.xhtml

Two different mortality projection analyses for 2015–20 were performed, each with a different observation base: one using 2001–10 data and the other using 2009–14 data, with the number of projected deaths based on 2001–10 data subtracted from the number of projected deaths based 2009–14 data. Using fixed-effects regression modeling, the total health and social care spending needed to close the mortality gap on top of planned spending budgets was computed for three different efficiency scenarios: 0%, 1% and 3% (see Supplementary Appendix for a definition and explanation).[16]

RESULTS

Health and social care spending

From 2001/02 to 2009/10, the average annual increase in real PEH per capita was 3.82%. Between 2010/11 and 2014/15, the average annual increase was 0.41% (Figure S1). Planned real PEH spending to 2020/21 is forecast to increase at an average of 0.72% per year. Real PES per capita experienced an average annual increase of 2.20% between 2001/02 and 2009/10, while between 2010/11 and 2014/15 it decreased by 1.57%.

Assessment of a mortality gap

From 2001 to 2010, the absolute number of deaths in England decreased by an average of 0.77% per year. From 2011 to 2014, the number of deaths increased by an average of 0.87% per year. To quantify the potential mortality gap associated with the 2010 spending constraints, we compared actual and predicted mortality rates using data from both sexes and found 8,148 higher-than-expected number of deaths in 2012 (95% confidence interval 2,004–14,292), 18,896 (12,641–

For peer review only - http://bmjopen.bmj.com/site/about/guidelines.xhtml
For peer review only

25,152) in 2013, and 18,324 (11,953–24,695) in 2014 (Figure 1). Sex-stratified time-series analyses revealed similar results between males and females (Table S1).

To validate these results, we looked at two alternative health outcomes: life expectancy and PYLL. We found 2012–14 male and female life expectancy to be 3.84 months (2.40–5.28) and 5.16 months (3.36–7.08) less, respectively, than the values anticipated from 2001–03 to 2009–11 data (Table S2). Moreover, we found that PYLL increased after the 2009/10 spending constraints compared with predicted rates (Table S3).

To see whether the possible effects of the 2010 spending constraints were sensitive to age, we conducted time-series analyses for ten different age groups (Table S4). Higher-than-expected numbers of deaths were confined to those ≥60 years of age with all six ≥60 age groups showed excess deaths in 2013. By contrast, <60 age groups most often exhibited fewer deaths.

We next looked at place of death for all age groups, while stratifying them by age into <60 and those ≥60 years. Time-trend analyses revealed care-home and home deaths to be the first and second largest contributors, respectively, to excess deaths across age groups and in those ≥60 (Figure 2, Figure S2 and Table S5), with a relative increase in these places of death over others during the study period. For every year analysed, lower-than-expected numbers of deaths occurred in hospitals. For those <60 years, hospitals were the most frequent place of death, and so the net result was a lower-than-expected number of <60 deaths. Time-series analyses by local government area (for time points up to 2013) revealed no correlation between changes in the number of expected deaths and deprivation (P=0.14, R_S=0.08).

For peer review only - http://bmjopen.bmj.com/site/about/guidelines.xhtml

Longitudinal associations between spending and mortality

Over the study period, we found that £10 per capita declines in real PEH and real PES were associated with increases of 0.19 (0.01–0.38) (P=0.049) and 5.10 (3.65–6.54) (P<0.001) care-home deaths per 100,000 in England, respectively (Table 1). For PES, these relationships persisted at least two years after the initial change but became weaker for PEH. We next sought to demonstrate that these associations were independent of macroeconomic forces, such as unemployment (for younger age groups) and pensions (for older age groups), which have each been linked with increased mortality.[17,18] On adjusting the regression models for macroeconomic factors and the annual average of weekly pensions, we found real PES per capita and real PEH per capita, remained inversely associated with care-home mortality (Table 1). Next, we used a regression model with both real PES and PEH per capita as explanatory variables, and found only PES remained inversely associated with care-home mortality (Table 1). The results for home deaths replicated those for care-home deaths (Table S6). To check whether PES was specifically related to care-home and home mortality rather than any place of death, we conducted the same set of analyses using hospital mortality as the response variable. In contrast to care-home and home mortality, real PEH per capita was more strongly related to hospital mortality than real PES per capita without adjusting for controls. However, on adjusting for macroeconomic forces, the relationship between PEH and hospital mortality was no longer apparent (Table S7).

For peer review only - http://bmjopen.bmj.com/site/about/guidelines.xhtml

Identification of resources potentially mediating the spending-mortality relationship

To discern the most important resources mediating the relationship between real PES per capita and care-home and home mortality, we collected data on workforce and bed numbers, and performed mediation analyses.

We were able to gather complete 2001–14 data for ten relevant health and social care resources (see Supplementary Appendix). Using these data, we found that the numbers of NHS hospital and community nurses, and NHS health and social care clinical support staff were each associated with care-home mortality while alleviating the relationship between real PES per capita and care-home mortality at lag years 1 and 2, suggesting these staff numbers were the most important mediators of the spending-mortality relationship (Table 2). When we looked at home mortality as the outcome, we found the number of nurses, but not clinical support staff, was suggested to be a mediator of the relationship between spending (both PEH and PES) and mortality rates for lag years 1 and 2 (Table S8). On repeating these analyses for PEH, we again found the number of nurses to be the sole mediator of the relationships between PEH per capita and care-home mortality (Table S9), and PEH per capita and home mortality (Table S10) for all years. In this regard, from 2001 to 2010, the average annual change in nurse numbers was 1.61 % whereas from 2010 to 2014, the average annual change was over twenty-fold lower at 0.07% (Table S11).

Mortality projections to 2020 and scenario modelling

To investigate the possibility of a future population mortality gap, we projected mortality rates to 2020 using two observation bases: one using 2001–10 data and a second using 2009–14 (2009 and 2010 were included to ensure a sufficiently large observation base). We found that the 2009–14-

For peer review only - http://bmjopen.bmj.com/site/about/guidelines.xhtml

based projection entailed an additional 152,141 deaths (aggregate 95% CI: 134,597–169,685) from 2015 to 2020 compared with the 2001–10-based projection (Figure S3).

To determine what it would take to close this gap above planned health and social care spending to 2020/21, we modelled three different scenarios (Figure 3 and Table S12). On top of the combined health and social care budget, the aggregate spending and efficiency combinations required to completely close the mortality gap would be: an additional £29.56 billion (£25.74–33.37 billion) for a conservative 0% annual efficiency gain; an additional £27.26 billion (£23.61–30.91 billion) for a moderate 1% annual efficiency gain, and an additional £23.03 billion (£19.67–26.39 billion) for an aggressive 3% annual efficiency gain (Figure 3 with an annual breakdown in Table S12). Under an ideal scenario in which no additional spending would be needed to close the mortality gap, the annual productivity increase to 2020 would need to reach 10.79% (7.8 to 14.46). In these scenarios, efficiency gains reflect improvements across the system required to meet respective spending constraints, while avoiding excess mortality.

DISCUSSION

This study demonstrates that recent constraints in PEH and PES spending in England have been associated with nearly 45,000 higher-than-expected numbers of deaths between 2012 and 2014. If these trends continue, even when considering the increased planned funding, we estimate approximately 150,000 additional deaths may arise between 2015 and 2020. Combining these projected excess deaths and the observed deaths prior to 2015 translate to around 95,000 excess

For peer review only - http://bmjopen.bmj.com/site/about/guidelines.xhtml

deaths from 2010 to 2016. Contemporaneous reductions in life expectancy and excesses in measures of preventable death validated our mortality findings.

The excess deaths observed in our study corroborated recent evidence highlighting the reversal of declining mortality trends observed in England and Wales in the past decade.[11] Similar to findings reported by Hiam and colleagues,[11] we observed that those aged ≥60 years are most susceptible to the observed excess mortality burden, as compared to those aged <60 years. We added to this evidence by additionally evaluated potential years of life lost (PYLL) and life expectancy as outcomes, projecting excess deaths to 2020, determining potential mediating factors which may explain the observed excess deaths, and testing different hypothetical scenarios of funding and efficiency improvement which may be able to close the mortality gap.

By setting, deaths at care homes and at home contributed most to the observed 'mortality gap', while hospital mortality was lower-than-expected. This is most likely because social care experienced greater relative spending constraints than healthcare. Furthermore, the recent drive to move patients with poor prognoses and who have reached their ceiling of care away from the hospital environment to care homes or their own homes may have contributed to this.[19] It is also important to consider the architectural differences between health and social care delivery in England. The NHS provides publicly delivered universal health coverage, with studies suggesting this confers a protective effect during episodes of substantial cost-constraint.[20] In contrast, social care is means-tested and often privately delivered, without universal coverage; factors that may influence access and quality.

For peer review only - http://bmjopen.bmj.com/site/about/guidelines.xhtml

The associations observed between PEH, PES, and mortality were independent of macroeconomic changes that often co-occur with periods of reduced public-sector spending. However, adjustment for health and social care resources, and nursing numbers in particular, nullified the associations between PEH, PES, and mortality. Our study suggests that the number of NHS-qualified nurses is the strongest tested mediator of the relationships between spending, and care-home and home mortality; this is congruent with findings in other reports.[21]

This study has several policy implications. First, it demonstrates that decelerated public health and social care funding increases in England may have adversely affected population mortality as demand increased and healthcare costs rose above inflation. This demonstrates that while health system design and ambition, such as delivery of universal coverage, is important, it must be adequately financed to ensure design translates into health improvement. Second, the finding that the elderly population and those in care homes were the most vulnerable to recent financial challenges, makes a strong case for targeted interventions to ensure adequate management of these patient groups.[4] This includes funding increases in social care, in addition to maintenance or rises in nursing numbers aligned with demand. Third, and perhaps most importantly, there remains a prospective cost to the current trajectory of system financing that entails a number of excess deaths. While it would be presumptive to class these deaths as avoidable (we have used the terms 'additional' or 'excess' to describe higher-than-expected numbers), we have outlined several funding-efficiency scenarios that attempt to demonstrate how such a gap could be closed. Given that the health system has historically achieved 1–2% annual productivity improvements, and that current demand is unprecedented, it seems unlikely that greater annual improvements could be

For peer review only - http://bmjopen.bmj.com/site/about/guidelines.xhtml

expected. This suggests that a cumulative spending increase of approximately £27.3 billion, which equates to around £6.8bn annually by 2020, may be required.

This is the first study to provide an in-depth analysis of the potential effects of public health and social care spending constraints on mortality. In this regard, we did not intend to analyze general changes in government policy, priorities, or the overall environment in which health and social spending operate. A limitation was that our study was observational and retrospective, lacking the resolution to robustly identify causal pathways. Second, between 2010 and 2012, nurse numbers dropped by approximately 6,000, which, by our regression analyses, translates to approximately 10% of explained deaths for that period. Therefore, the changes in NHS-qualified nurse numbers must only be partly responsible for the putative relationship between spending and mortality. Other resources and complex, emergent behavior, which do not depend solely on the component parts of the health and social care system but rather on their interactions with one another, might also explain some of the additional deaths observed.

CONCLUSION

We have found that spending constraints since 2010, especially PES, may have produced a substantial mortality gap in England. Our analyses demonstrate that if demand-side solutions are infeasible or otherwise disagreeable, large improvements in efficiency or, more feasibly, spending above growth in demand (and not just general inflation) are required to close this gap. We suggest that spending should be targeted on improving care delivered in care homes and at home; and maintaining or increasing nurse numbers.

For peer review only - http://bmjopen.bmj.com/site/about/guidelines.xhtml

AUTHOR CONTRIBUTIONS

JW and MM conceived and designed the study. JW, WW, CDZ, DM and MM obtained the data. JW, WW, GDS, PDR, and VAM conducted data formatting, and JW and WW carried out statistical analysis with input from LPK. All authors helped interpret the findings. JW, WW, and MM wrote the first draft of the manuscript with input from DM, CDZ, RR, and LPK; all authors provided input to subsequent drafts. All authors had full access to all of the data in the study and can take responsibility for its integrity and the accuracy of the data analysis.

ACKNOWLEDGEMENTS

The authors would like to thank Mr. Andrew Pring of Public Health England for extracting data used in the place-of-death analyses and Ms. Lauren Passby and Ms. Peggy Fooks for help regarding the initial data collection effort.

FUNDING

No funding was received for this study.

COMPETING INTERESTS

MM serves as an advisor to NHS England and is co-founder of Cera. However, the views expressed in this manuscript are his own and not those of Cera or NHS England.

ETHICAL APPROVAL

Not required.

DATA SHARING

All data are in the public domain.

For peer review only

For peer review only

For peer review only - http://bmjopen.bmj.com/site/about/guidelines.xhtml

REFERENCES

1. Ham C, Imison C, Goodwin N, Dixon A, South P. Where next for the NHS reforms? The case for integrated care [Internet]. London: The King's Fund; 2011. Available from: https://www.kingsfund.org.uk/sites/files/kf/where-next-nhs-reforms-case-for-integrated-care-ham-imison-goodwin-dixon-south-kings-fund-may-2011.pdf
2. Lutz W, O'Neill BC, Scherbov S. Demographics. Europe's population at a turning point. *Science*. 2003 Mar 28;**299**(5615):1991–2.
3. Okunade AA, Murthy VNR. Technology as a 'major driver' of health care costs: A cointegration analysis of the Newhouse conjecture. *J Health Econ*. 2002;**21**:147–159.
4. NHS England, Care Quality Commission, Public Health England, Monitor, Health Education England, NHS Trust Development Authority. NHS five year forward view. [Internet]. NHS England; 2014. Available from: https://www.england.nhs.uk/wp-content/uploads/2014/10/5yfv-web.pdf
5. Local Government Association. Adult social care funding: 2016 state of the nation report [Internet]. London: LGA; 2016. Available from: http://www.local.gov.uk/documents/10180/7632544/1+24+ASCF+state+of+the+nation+2016_WEB.pdf/e5943f2d-4dbd-41a8-b73e-da0c7209ec12
6. Miller C, Bunnin A, Rayner V. Older people who self fund their social care: A guide for health and wellbeing boards and commissioners. London: Office For Public Management; 2013.
7. Office for National Statistics. Population projections [Internet]. 2014. Available from: https://www.ons.gov.uk/peoplepopulationandcommunity/populationandmigration/populationprojections
8. Donnelly L. A&E crisis deepens with 65 hospital trusts issuing emergency alerts [Internet]. 2017. Available from: http://www.telegraph.co.uk/news/2017/01/13/ae-crisis-deepens-58-hospital-trusts-issuing-emergency-alerts/
9. NHS England. A&E Attendances and Emergency Admissions [Internet]. Available from: https://www.england.nhs.uk/statistics/statistical-work-areas/ae-waiting-times-and-activity/
10. UK Parliament. Impact of the Spending Review on health and social care [Internet]. 2016. Available from: https://www.publications.parliament.uk/pa/cm201617/cmselect/cmhealth/139/13902.htm?utm_source=139&utm_medium=fullbullet&utm_campaign=Modulereports
11. Hiam L, Dorling D, Harrison D, McKee M. Why has mortality in England and Wales been increasing? An iterative demographic analysis. *J R Soc Med*. 2017;
12. Office for National Statistics. Mortality statistics: deaths registered in England and Wales [Internet]. 2014. Available from: http://webarchive.nationalarchives.gov.uk/20160105160709/http://www.ons.gov.uk/ons/rel/vsob1/mortality-statistics--deaths-registered-in-england-and-wales--series-dr-/index.html
13. UK Health and Social Care Information Centre. NHS Outcomes Framework Indicators [Internet]. *May 2015* 2015. Available from: http://content.digital.nhs.uk/nhsof
14. Office for National Statistics. Unemployment in England, 1992-2015 [Internet]. 2015. p.

For peer review only - http://bmjopen.bmj.com/site/about/guidelines.xhtml

https://www.nomisweb.co.uk/reports/lmp/gor/2092957. Available from: https://www.ons.gov.uk/employmentandlabourmarket/peoplenotinwork/unemployment

15. Office for National Statistics. Consumer Price Inflation, release [Internet]. 2014. Available from: http://webarchive.nationalarchives.gov.uk/20160105160709/http://ons.gov.uk/ons/rel/cpi/consumer-price-indices/december-2014/index.html

16. NHS England. NHS Five Year Forward View. Recap briefing for the Health Select Committee on technical modelling and scenarios [Internet]. 2016. Available from: https://www.england.nhs.uk/wp-content/uploads/2016/05/fyfv-tech-note-090516.pdf

17. Maruthappu M, Watkins J, Noor AM, et al. Economic downturns , universal health coverage , and cancer mortality in high-income and middle-income countries , 1990 – 2010: a longitudinal analysis. *Lancet* [Internet]. Elsevier Ltd; 2016;**6736**(16):1–12. Available from: http://dx.doi.org/10.1016/S0140-6736(16)00577-8

18. Loopstra R, McKee M, Katikireddi SV, Taylor-Robinson D, Barr B, Stuckler D. Austerity and old-age mortality in England: a longitudinal cross-local area analysis, 2007-2013. *J R Soc Med* [Internet]. 2016 Mar;**109**(3):109–16. Available from: http://www.ncbi.nlm.nih.gov/pubmed/26980412

19. Department of Health. NHS-funded Nursing Care Practice Guide [Internet]. 2013. Available from: https://www.gov.uk/government/uploads/system/uploads/attachment_data/file/211256/NHS-funded_Nursing_Care_Best_Practice_Guidance.pdf

20. Karanikolos M, Mladovsky P, Cylus J, et al. Financial crisis, austerity, and health in Europe. *Lancet* [Internet]. Elsevier Ltd; 2013 Apr 13 [cited 2014 Jul 27];**381**(9874):1323–1331. Available from: http://www.ncbi.nlm.nih.gov/pubmed/23541059

21. Mid Staffordshire NHS Foundation Trust. Final Report [Internet]. 2013. Available from: http://webarchive.nationalarchives.gov.uk/20150407084003/http://www.midstaffspublicinquiry.com/report

For peer review only

For peer review only - http://bmjopen.bmj.com/site/about/guidelines.xhtml

Table 1. Changes in care-home deaths per 100,000 persons for every £10 change in public expenditure in health (PEH) or social care (PES). Associations were evaluated for up to 2 years of interval between PEH or PES and subsequent care-home deaths. Number of observations (sex-years) per analysis is 28.

	Lag (year)	PEH per capita (£10)		PES per capita (£10)	
		**β (95% CI)**	**p -value**	**β (95% CI)**	**p-value**
**Model 1**	0	-0.19 (-0.38 to -0.01)	0.05	-5.10 (-6.54 to -3.65)	<0.0001
	1	-0.09 (-0.28 to 0.11)	0.39	-3.54 (-5.43 to -1.65)	0.001
	2	0.06 (-0.13 to 0.25)	0.52	-1.38 (-3.54 to 0.79)	0.23
**Model 2**	0	-0.63 (-0.78 to -0.47)	<0.0001	-5.21 (-6.49 to -3.93)	<0.0001
	1	-0.49 (-0.65 to -0.33)	<0.0001	-4.34 (-5.70 to -2.99)	<0.0001
	2	-0.22 (-0.44 to -0.01)	0.05	-2.73 (-4.40 to -1.06)	0.005
**Model 3**	0	-0.88 (-1.11 to -0.65)	<0.0001	-5.59 (-7.06 to -4.11)	<0.0001
	1	-0.82 (-1.01 to -0.63)	<0.0001	-5.24 (-6.60 to -2.26)	<0.0001
	2	-0.55 (-0.78 to -0.32)	0.0002	-3.82 (-5.39 to -2.26)	<0.0001
**Model 4**	0	0.14 (-0.01 to 0.29)	0.08	-6.23 (-8.10 to -4.39)	<0.0001
	1	0.37 (0.16 to 0.56)	0.002	-7.05 (-9.55 to -4.56)	<0.0001
	2	0.60 (0.35 to 0.85)	0.0001	-7.40 (-10.31 to -4.49)	<0.0001

Model 1: Unadjusted model
Model 2: Adjusted for basic state pension per week
Model 3: Adjusted for unemployment rate and CPI
Model 4: PEH and PES were included in the same model

For peer review only

Table 2. Changes in care-home deaths per 100,000 persons associated with changes in public expenditure in social care (PES), adjusted for quantity of resources. Associations were evaluated for up to 2 years of interval between PES and subsequent care-home deaths.

Resource	Lag (year)	Care-home deaths per 100,000 persons			
		PES in 10 million (£)		Resource quantity (thousands)[1]	
		β (95% CI)	p-value	β (95% CI)	p-value
No. of hospital doctors	0	-6.01 (-7.64 to -4.38)	<0.0001	0.21 (0.006 to 0.41)	0.06
	1	-6.66 (-8.65 to -4.38)	<0.0001	0.57 (0.32 to 0.83)	0.0002
	2	-6.85 (-9.03 to -4.67)	<0.0001	0.94 (0.64 to 1.24)	<0.0001
No. of GPs	0	-6.01 (-7.66 to -4.36)	<0.0001	0.87 (-0.009 to 1.76)	0.06
	1	-6.52 (-8.53 to -4.51)	<0.0001	2.35 (1.25 to 3.45)	0.0004
	2	-6.28 (-8.42 to -4.15)	<0.0001	3.67 (2.43 to 4.90)	<0.0001
No. of nurses	0	-4.49 (-6.38 to -2.59)	0.0001	-0.09 (-0.28 to 0.09)	0.34
	1	-0.84 (-2.69 to 1.01)	0.39	-0.40 (-0.58 to -0.29)	0.0001
	2	1.80 (-0.12 to 3.73)	0.08	-0.48 (-0.67 to -0.29)	<0.0001
No. of scientific, therapeutic and technical staff	0	-6.15 (-7.83 to -4.48)	<0.0001	-0.20 (0.01 to 0.38)	0.04
	1	-6.89 (-8.96 to -4.82)	<0.0001	0.52 (0.28 to 0.76)	0.0002
	2	-7.12 (-9.31 to -4.93)	<0.0001	0.85 (0.59 to 1.11)	<0.0001
No. of ambulance staff	0	-6.09 (-7.73 to -4.46)	<0.0001	2.27 (0.17 to 4.37)	0.04
	1	-5.94 (-8.11 to -3.77)	<0.0001	4.60 (1.78 to 7.42)	0.004
	2	-5.09 (-7.70 to -2.47)	0.001	6.72 (3.14 to 10.29)	0.001
No. of clinical support staff	0	-5.01 (-6.74 to -3.38)	<0.0001	-0.02 (-0.26 to 0.22)	0.85
	1	-1.72 (-3.75 to 0.31)	0.11	-0.42 (-0.69 to -0.14)	0.007
	2	-0.49 (-1.87 to 2.86)	0.69	-0.43 (-0.75 to -0.11)	0.02
No. of infrastructure support staff	0	-5.78 (-8.63 to -2.91)	0.0006	0.09 9-0.23 to 0.40)	0.59
	1	-5.31 (-9.43 to -1.20)	0.02	0.21 (-0.23 to 0.66)	0.35
	2	-6.55 (-10.71 to -2.40)	0.006	0.62 (0.18 to 1.06)	0.01
No. of overnight beds	0	-5.56 (-7.10 to -4.19)	<0.0001	-0.13 (-0.25 to -0.008)	<0.0001
	1	-5.42 (-6.95 to -3.90)	<0.0001	-0.34 (-0.48 to -0.21)	<0.0001
	2	-4.38 (-5.92 to -2.84)	<0.0001	-0.50 (-0.65 to -0.35)	<0.0001
No. of social care staff with accommodation	0	-5.20 (-6.64 to -3.77)	0.01	0.09 (-0.05 to 0.23)	0.21
	1	-4.12 (-5.94 to -2.29)	0.01	0.22 (0.02 to 0.42)	0.04
	2	-2.03 (-4.21 to 0.16)	0.01	0.22 (-0.03 to 0.47)	0.09
No. of social care staff without accommodation	0	-5.69 (-7.19 to -4.20)	<0.0001	0.02 (-0.0002 to 0.04)	0.06
	1	-5.52 (-7.21 to -3.83)	<0.0001	0.05 (0.03 to 0.07)	0.0003
	2	-4.67 (-6.30 to -3.09)	0.009	0.08 (0.05 to 0.10)	<0.0001

[1]Estimates are shown for each corresponding resource in the left-hand column.

For peer review only - http://bmjopen.bmj.com/site/about/guidelines.xhtml

FIGURE LEGENDS

Figure 1: Time-trend projections of ASDR per 100,000 individuals. ASDR (left hand y-axis) and the difference in the number of deaths between actual and predicted mortality (right hand y-axis) per year from 2001 to 2014 are shown. The black and blue lines represent actual ASDR for the 2001–10 and 2011–14 periods, respectively. The red line represents predicted ASDR using 2001–10 as an observation base while the 95% CIs are denoted by the beige-colored area. The grey bars denote the differences between the number of deaths observed and the number predicted for 2011–14 where positive values correspond to excess deaths and negative values represent lower-than-expected deaths. Error bars represent 95% CIs. *P < 0.05, **P < 0.01 and ***P < 0.001.

Figure 2: Numbers of excess or lower-than-expected deaths for each place of death. Separate time-trend analyses comparing actual to predicted mortality from 2011 to 2014 were conducted using mortality data categorized by place of death. Contributions from each place of death are color-coded. Data are shown for mortality rates for all ages (top panel), those under 60 (middle panel), and those 60 years or over (bottom panel). *P < 0.05, **P < 0.01 and ***P < 0.001.

Figure 3: Additional spending needed to close the 2020 mortality gap. Data are shown for PEH (blue) and on top of that, PES (beige), in real terms according to 2014/15 prices. Actual outturn data are shown for 2001/02 to 2014/15. However, for 2015/16 to 2020/21, the budgeted total Department of Health expenditure limit is shown for PEH. For PES, data are based on the continuation of -2.25% annual percentage change in core PES in 2014/15 supplemented by the potential revenue from the adult social care precept for council tax.[11] Additional spending needed to close the projected mortality gap for each year from 2015 to 2020 is shown as color-coded dotted lines for three different scenarios, each of which assumes different annual efficiency improvements. The additional annual spending numbers with associated 95% CI are shown as bar plots for each scenario.

SUPPLEMENTARY FIGURE LEGENDS

Figure S1: Public-sector expenditure on healthcare (PEH) and social care (PES). Data are shown from 2001/02–2014/15. Top plot shows data for PEH whereas bottom plot shows data for

For peer review only - http://bmjopen.bmj.com/site/about/guidelines.xhtml

PES. Solid lines correspond to total PEH or PES in billions of pounds (left hand *y*-axis). Dotted lines correspond to PEH or PES in pounds per capita (right hand y-axis) calculated from PEH/PES and population size as estimated by the UK's Office for National Statistics (ONS). Blue lines indicate nominal expenditure. Red lines indicate real expenditure adjusted to 2014/15 prices.

Figure S2: Time-trend projections of mortality rates by place of death. Mortality rates (y-axis) per year from 2001 to 2014 are shown for five places of death with 'Other' representing any place of death that doesn't fall into the other four categories (data are unavailable for a breakdown of where these other places might be). The black and blue lines represent actual ASDR for the 2001–10 and 2011–14 periods, respectively. The red line represents predicted ASDR using 2001–10 as an observation base while the 95% CIs are denoted by the beige-colored area.

Figure S3: **Population mortality projections to 2020**. Age-standardised death rates (ASDR) (left hand y-axis) and difference in the number of deaths between actual and predicted rates (right hand y-axis) projected annually to 2020 are shown. Black lines indicate 2009–14 base-fitted (black solid line) and 2015–20 base-forecasted (black dotted line) values. Red lines indicate the same using an observation base for the years 2001–2010. The bisque-coloured area denotes the 95% CIs for the model for 2009–14 data, and the pink area, the 95% CIs for the model for 2001–10 data. The grey bars represent the difference between the number of deaths projected using a 2009–14 observation base and the number forecasted using a 2001–10 observation base, where positive values correspond to excess deaths. Error bars signify 95% CIs. *P < 0.05, **P < 0.01 and ***P < 0.001.

For peer review only

For peer review only - http://bmjopen.bmj.com/site/about/guidelines.xhtml

Figure 1

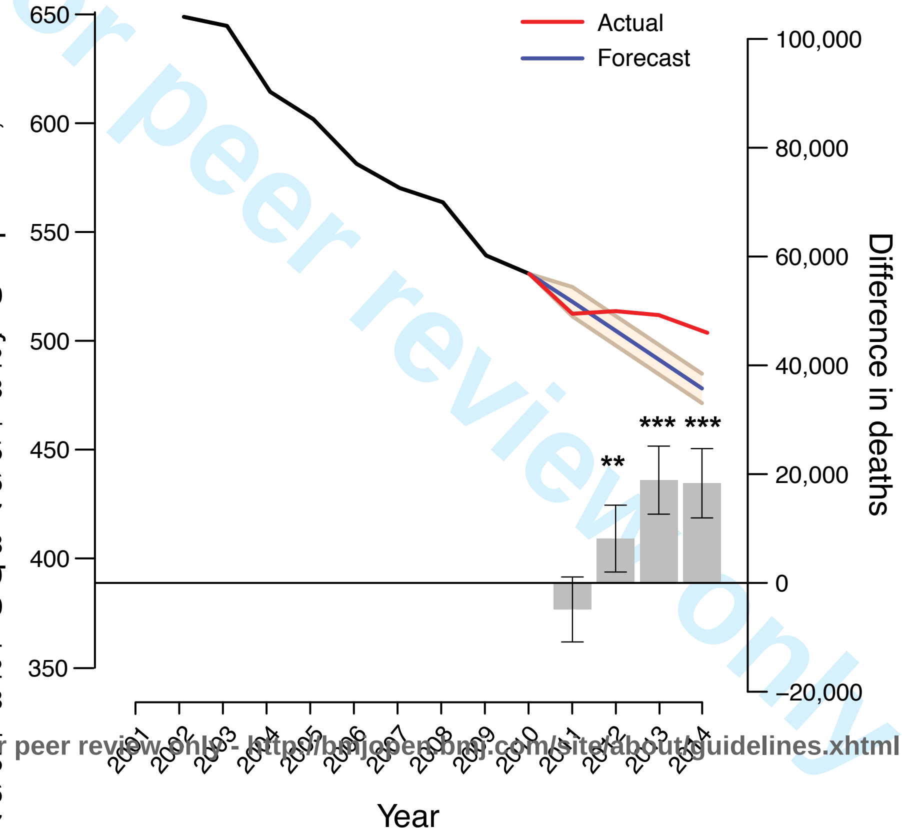

For peer review only

Figure 2

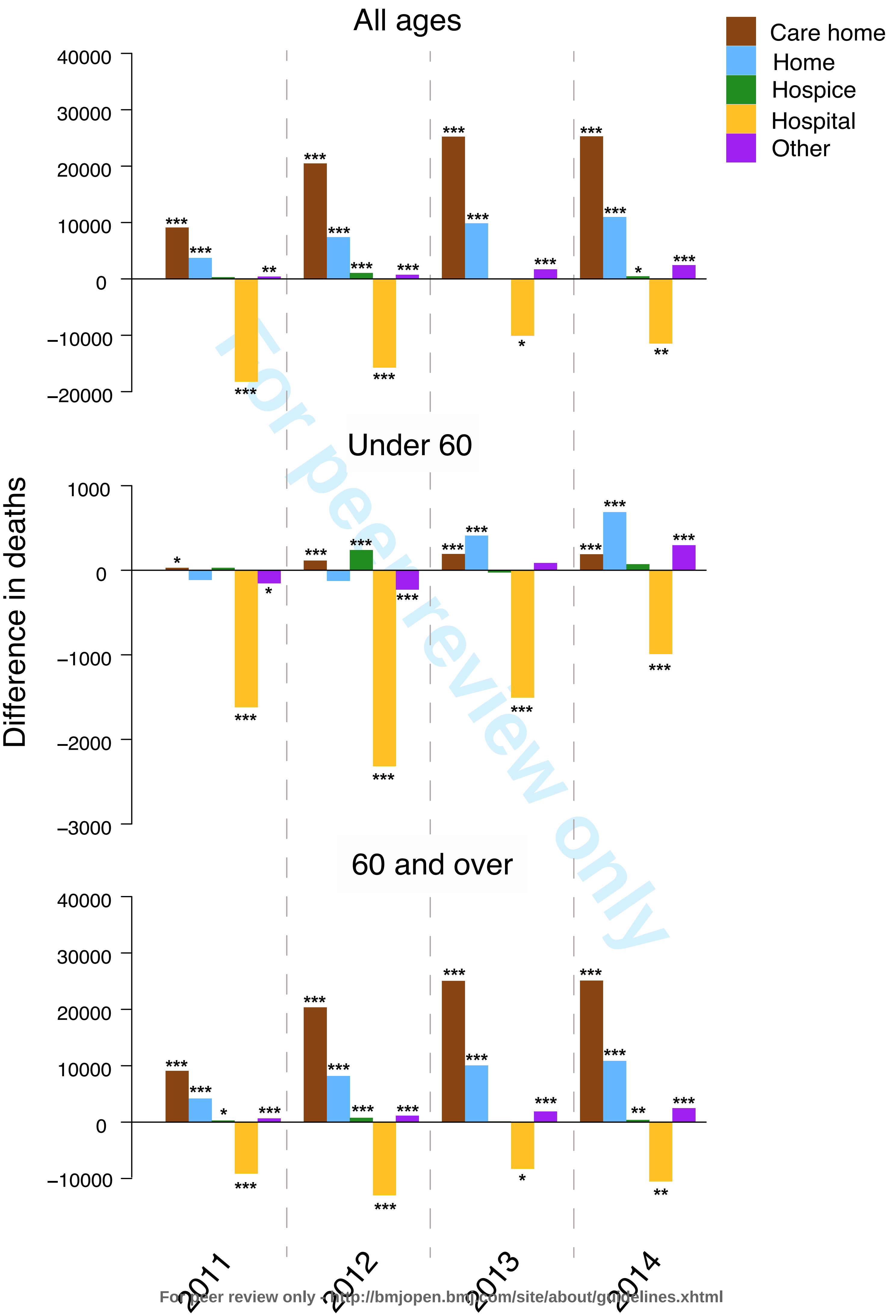

For peer review only

BMJ Open

Figure 3

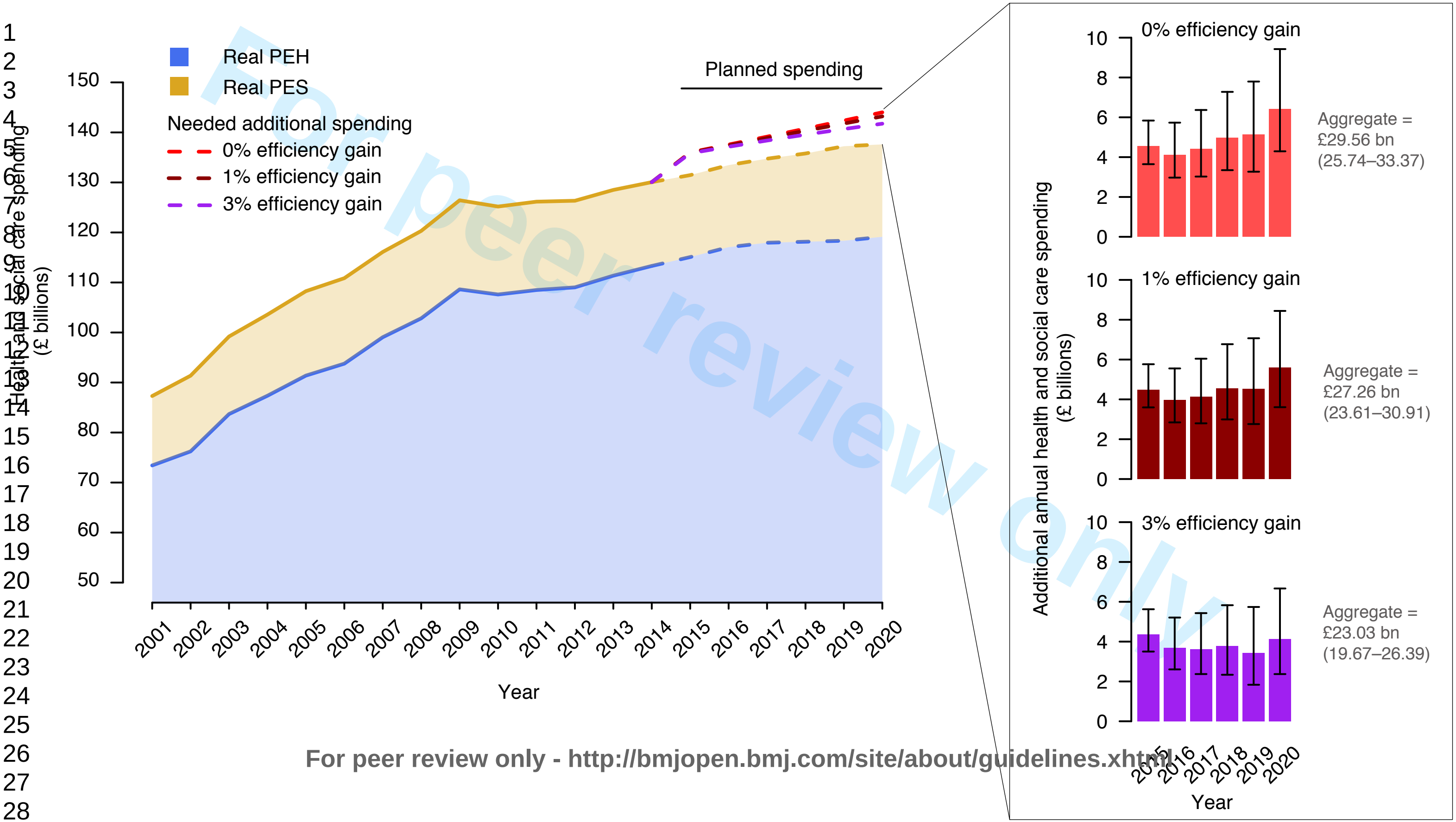

For peer review only

Table S1. Absolute and relative differences between observed and predicted age-standardised death rates overall and by sex, 2011-2014.

	No. of observed deaths	Difference in deaths (95% CI)	Rate ratio (95% CI)	p-value
**Both men and women**				
2011	452860	-4875.2 (-10850 to 1099.8)	0.99 (0.98 to 1.00)	0.11
2012	466780	8147.8 (2003.6 to 14292)	1.02 (1.00 to 1.03)	0.009
2013	473550	18896 (12641 to 25152)	1.04 (1.03 to 1.06)	<0.0001
2014	468880	18324 (11953 to 24695)	1.04 (1.03 to 1.06)	<0.0001
**Men**				
2011	219070	-1591.2 (-3917.4 to 734.98)	0.99 (0.98 to 1.00)	0.18
2012	224460	3710.4 (1312 to 6108.8)	1.02 (1.01 to 1.03)	0.0003
2013	229290	10166 (7709.7 to 12622)	1.05 (1.03 to 1.06)	<0.0001
2014	229120	11298 (8777.2 to 13819)	1.05 (1.04 to 1.06)	<0.0001
**Women**				
2011	233790	-2888.6 (-6692.4 to 915.16)	0.99 (0.97 to 1.00)	0.14
2012	242320	5343.6 (1444.8 to 9242.3)	1.02 (1.01 to 1.04)	0.01
2013	244260	9530 (5574.5 to 13486)	1.04 (1.02 to 1.06)	<0.0001
2014	239760	8054.3 (4052.6 to 12056)	1.03 (1.02 to 1.05)	<0.0001

For peer review only

Table S2. Predicted and observed life expectancy (years) overall and by sex, 2010-2014.

	Life expectancy (years)		p-value
	Predicted	Observed	
**2010-2012**			
Male	79.05 (78.98 to 79.12)	79.03	0.71
Female	82.95 (82.86 to 83.04)	82.83	0.08
**2011-2013**			
Male	79.37 (79.27 to 79.47)	79.23	0.009
Female	83.21 (83.08 to 83.33)	82.97	0.0007
**2012-2014**			
Male	79.69 (79.57 to 79.81)	79.37	<0.0001
Female	83.46 (83.31 to 83.62)	83.03	<0.0001

For peer review only

Table S3. Absolute and relative differences between predicted and observed age-standardised PYLL overall and by sex, 2011-2014.

	No. of observed PYLL	Difference in PYLL (95% CI)	Rate ratio (95% CI)	p-value
**Both men and women**				
2011	1110600	5878.8 (214.85 to 11543)	1.01 (1.00 to 1.01)	0.04
2012	1074400	-9359.3 (-15124 to -3594.2)	0.99 (0.99 to 1.00)	0.002
2013	1093200	22568 (16650 to 28485)	1.02 (1.02 to 1.03)	<0.0001
2014	1193000	75496 (69069 to 81923)	1.07 (1.06 to 1.07)	<0.0001
**Men**				
2011	610170	2882.3 (-3093.8 to 8858.5)	1.00 (0.99 to 1.01)	0.35
2012	583320	1794.9 (-4152 to 7741. 8)	1.00 (0.99 to 1.01)	0.57
2013	600650	27893 (21797 to 33989)	1.05 (1.04 to 1.06)	<0.0001
2014	654510	44309 (37538 to 51080)	1.07 (1.06 to 1.08)	<0.0001
**Women**				
2011	500460	3368.2 (487.67 to 6248.7)	1.01 (1.00 to 1.01)	0.02
2012	491090	17219 (14373 to 20064)	1.04 (1.03 to 1.04)	<0.0001
2013	492530	24602 (21688 to 27517)	1.05 (1.05 to 1.06)	<0.0001
2014	538480	32157 (28881 to 35434)	1.06 (1.06 to 1.07)	<0.0001

For peer review only

Table S4. Overall and age group-specific relative differences between observed and predicted death rates, 2011-2014.

	Rate ratio (95% CI)	p-value
**2011**		
All ages (ASR)	0.99 (0.98 to1.00)	0.11
0-34	0.94 (0.92 to 0.96)	<0.0001
35-49	0.96 (0.94 to 0.97)	<0.0001
50-54	0.98 (0.96 to 0.99)	<0.0001
55-59	0.98 (0.97 to 0.99)	0.01
60-64	1.01 (1.01 to 1.02)	0.003
65-69	0.99 (0.98 to 1.00)	0.03
70-74	1.03 (1.02 to 1.05)	<0.0001
75-79	0.99 (0.98 to 1.00)	0.16
80-84	0.96 (0.94 to 0.98)	<0.0001
85 and older	0.99 (0.97 to 1.02)	0.59
**2012**		
All ages (ASR)	1.02 (1.00 to 1.03)	0.01
0-34	0.92 (0.90 to 0.93)	<0.0001
35-49	0.92 (0.91 to 0.93)	<0.0001
50-54	0.96 (0.94 to 0.97)	<0.0001
55-59	0.97 (0.96 to 0.99)	0.0001
60-64	1.04 (1.03 to 1.05)	<0.0001
65-69	1.00 (0.99 to 1.00)	0.32
70-74	1.06 (1.05 to 1.07)	<0.0001
75-79	1.03 (1.02 to 1.04)	<0.0001
80-84	0.99 (0.97 to 1.01)	0.33
85 and older	1.06 (1.03 to 1.08)	<0.0001
**2013**		
All ages (ASR)	1.04 (1.03 to 1.06)	<0.0001
0-34	0.93 (0.91 to 0.94)	<0.0001
35-49	0.94 (0.93 to 0.95)	<0.0001
50-54	0.97 (0.96 to 0.99)	<0.0001
55-59	0.98 (0.96 to 0.99)	<0.0001
60-64	1.08 (1.06 to 1.09)	<0.0001
65-69	1.01 (1.00 to 1.02)	0.02
70-74	1.09 (1.07 to 1.10)	<0.0001
75-79	1.06 (1.05 to 1.08)	<0.0001
80-84	1.01 (0.99 to 1.03)	0.28
85 and older	1.09 (1.06 to 1.11)	<0.0001
**2014**		
All ages (ASR)	1.04 (1.03 to 1.06)	<0.0001
0-34	0.97 (0.95 to 0.99)	0.001
35-49	0.94 (0.93 to 0.95)	<0.0001
50-54	0.98 (0.97 to 1.00)	0.02
55-59	0.97 (0.95 to 0.98)	<0.0001
60-64	1.10 (1.09 to 1.11)	<0.0001
65-69	1.03 (1.02 to 1.04)	<0.0001
70-74	1.11 (1.09 to 1.12)	<0.0001
75-79	1.07 (1.06 to 1.09)	<0.0001
80-84	1.00 (0.98 to 1.02)	0.64
85 and older	1.06 (1.03 to 1.09)	<0.0001

For peer review only

Table S5. Overall and age group-specific relative differences between observed and predicted death rates by place of death, 2011-2014.

	All ages (ASR)		0-59		60+	
	**Rate ratio (95% CI)**	**p-value**	**Rate ratio (95% CI)**	**p-value**	**Rate ratio (95% CI)**	**p-value**
**Care-home**						
2011	1.12 (1.07 to 1.17)	<0.0001	1.03 (1.00 to 1.07)	0.03	1.12 (1.07 to 1.17)	<0.0001
2012	1.26 (1.21 to 1.33)	<0.0001	1.13 (1.10 to 1.17)	<0.0001	1.26 (1.21 to 1.33)	<0.0001
2013	1.33 (1.27 to 1.40)	<0.0001	1.23 (1.19 to 1.27)	<0.0001	1.33 (1.27 to 1.40)	<0.0001
2014	1.33 (1.27 to 1.40)	<0.0001	1.22 (1.18 to 1.26)	<0.0001	1.33 (1.27 to 1.41)	<0.0001
**Home**						
2011	1.04 (1.02 to 1.06)	<0.0001	0.99 (0.98 to 1.01)	0.30	1.05 (1.03 to 1.08)	<0.0001
2012	1.08 (1.06 to 1.10)	<0.0001	0.99 (0.98 to 1.01)	0.26	1.10 (1.08 to 1.13)	<0.0001
2013	1.10 (1.08 to 1.12)	<0.0001	1.03 (1.03 to 1.04)	<0.0003	1.13 (1.11 to 1.15)	<0.0001
2014	1.11 (1.09 to 1.13)	<0.0001	1.04 (1.03 to 1.06)	<0.0001	1.14 (1.11 to 1.16)	<0.0001
**Hospital**						
2011	0.93 (0.90 to 0.96)	<0.0001	0.94 (0.92 to 0.96)	<0.0001	0.93 (0.90 to 0.96)	<0.0001
2012	0.94 (0.91 to 0.97)	0.0001	0.91 (0.89 to 0.93)	<0.0001	0.94 (0.89 to 0.93)	0.0007
2013	0.96 (0.93 to 0.99)	0.01	0.94 (0.92 to 0.96)	<0.0001	0.96 (0.93 to 1.00)	0.03
2014	0.95 (0.92 to 0.99)	0.005	0.96 (0.94 to 0.98)	0.0002	0.95 (0.92 to 0.99)	0.008
**Hospice**						
2011	1.01 (1.00 to 1.03)	0.09	1.01 (0.99 to 1.02)	0.49	1.01 (1.00 to 1.03)	0.04
2012	1.04 (1.00 to 1.03)	<0.0001	1.04 (1.03 to 1.05)	<0.0001	1.04 (1.02 to 1.05)	<0.0001
2013	1.00 (0.99 to 1.02)	0.58	1.00 (0.99 to 1.02)	0.55	1.01 (0.99 to 1.02)	0.29
2014	1.02 (1.00 to 1.03)	0.01	1.01 (1.00 to 1.03)	0.08	1.02 (1.01 to 1.03)	0.006
**Other**						
2011	1.04 (1.02 to 1.07)	0.002	0.96 (0.94 to 0.99)	0.01	1.14 (1.09 to 1.19)	<0.0001
2012	1.08 (1.05 to 1.11)	<0.0001	0.95 (0.92 to 0.98)	0.0003	1.27 (1.21 to 1.33)	<0.0001
2013	1.20 (1.16 to 1.23)	<0.0001	1.02 (0.99 to 1.05)	0.17	1.48 (1.40 to 1.57)	<0.0001
2014	1.30 (1.26 to 1.34)	<0.0001	1.07 (1.04 to 1.10)	<0.0001	1.71 (1.61 to 1.83)	<0.0001

For Peer Review Only

Table S6. Changes in home deaths per 100,000 persons for every £10 change in public expenditure in health (PEH) or social care (PES). Associations were evaluated for up to 2 years of interval between PEH or PES and subsequent home deaths. Number of observations (sex-years) per analysis is 28.

	Lag (year)	PEH per capita (£10)		PES per capita (£10)	
		β (95% CI)	p -value	β (95% CI)	p-value
**Model 1**	0	-0.13 (-0.21 to -0.05)	0.005	-2.63 (-3.27 to -1.99)	<0.0001
	1	-0.07 (-0.15 to 0.02)	0.13	-1.78 (-2.54 to -1.01)	0.0002
	2	0.005 (-0.07 to 0.08)	0.90	-0.71 (-1.58 to 0.16)	0.12
**Model 2**	0	-0.31 (-0.39 to -0.22)	<0.0001	-2.65 (-3.30 to -2.01)	<0.0001
	1	-0.25 (-0.33 to -0.15)	<0.0001	-2.05 (-2.69 to -1.40)	<0.0001
	2	-0.20 (-0.29 to -0.11)	0.0002	-1.22 (-1.93 to -0.52)	0.003
**Model 3**	0	-0.41 (-0.53 to -0.29)	<0.0001	-2.68 (-3.40 to -1.96)	<0.0001
	1	-0.36 (-0.46 to -0.26)	<0.0001	-2.32 (-3.09 to -1.54)	<0.0001
	2	-0.25 (-0.35 to -0.14)	0.0002	-1.17 (-2.56 to -0.88)	0.0008
**Model 4**	0	0.02 (-0.05 to 0.09)	0.62	-2.78 (-3.65 to -1.91)	<0.0001
	1	0.12 (0.03 to 0.21)	0.01	-2.96 (-4.06 to -1.86)	<0.0001
	2	0.21 (0.09 to 0.32)	0.002	-2.79 (-4.11 to -1.47)	0.0005

Model 1: Unadjusted model
Model 2: Adjusted for basic state pension per week
Model 3: Adjusted for unemployment rate and CPI
Model 4: PEH and PES were included in the same model

For peer review only

Table S7. Changes in hospital deaths per 100,000 persons for every £10 change in public expenditure in health (PEH) or social care (PES). Associations were evaluated for up to 2 years of interval between PEH or PES and subsequent hospital deaths. Number of observations (sex-years) per analysis is 28.

	Lag (year)	PEH per capita (£10)		PES per capita (£10)	
		**β (95% CI)**	**p -value**	**β (95% CI)**	**p-value**
**Model 1**	0	-2.60 (-3.11 to -2.08)	<0.0001	-11.13 (-2.62 to 2.35)	0.12
	1	-2.70 (-3.11 to -2.29)	<0.0001	-19.92 (-31.80 to -8.04)	0.003
	2	-2.66 (-2.30 to -2.35)	<0.0001	-24.02 (-33.30 to -14.74)	<0.0001
**Model 2**	0	-1.36 (-1.79 to -0.94)	<0.0001	-9.11 (-13.57 to -4.65)	0.0005
	1	-1.66 (-1.94 to -1.38)	<0.0001	-12.69 (-15.96 to -9.42)	0.0002
	2	-1.78 (-2.07 to -1.49)	<0.0001	-13.21 (-15.49 to -10.94)	<0.0001
**Model 3**	0	-0.33 (-0.76 to 0.11)	0.16	-1.39 (-4.24 to 1.46)	0.35
	1	-0.07 (-0.12 to -0.03)	0.003	-6.37 (-9.14 to -3.60)	0.01
	2	-0.11 (-0.16 to -0.05)	0.001	-10.14 (-12.81 to -7.48)	0.0006
**Model 4**	0	-3.56 (-3.96 to -3.15)	<0.0001	17.98 (12.95 to 23.02)	<0.0001
	1	-3.69 (-4.11 to -3.27)	<0.0001	15.49 (10.31 to 20.66)	<0.0001
	2	-3.36 (-3.84 to -2.89)	<0.0001	9.73 (4.11 to 15.35)	0.003

Model 1: Unadjusted model
Model 2: Adjusted for basic state pension per week
Model 3: Adjusted for unemployment rate and CPI
Model 4: PEH and PES were included in the same model

For peer review only

Table S8. Changes in home deaths per 100,000 persons associated with changes in public expenditure in social care (PES), adjusted for quantity of resources. Associations were evaluated for up to 2 years of interval between real PES per capita and subsequent home deaths. Number of observations (sex-years) per analysis is 28.

Resource	Lag (year)	Home deaths per 100,000 persons			
		PES per capita (£10)		Resource quantity (thousands)[1]	
		β (95% CI)	p-value	β (95% CI)	p-value
No. of hospital doctors	0	-2.76 (-3.53 to -1.98)	<0.0001	0.03 (-0.07 to 0.12)	0.57
	1	-2.86 (-3.76 to -1.97)	<0.0001	0.20 (-0.08 to 0.31)	0.003
	2	-2.74 (-3.72 to -1.76)	<0.0001	0.35 (0.21 to 0.49)	<0.0001
No. of GPs	0	-2.73 (-3.52 to -1.95)	<0.0001	0.10 (-0.32 to 0.52)	0.0005
	1	-2.76 (-3.68 to -1.84)	<0.0001	0.78 (-0.28 to 1.28)	0.001
	2	-2.51 (-3.47 to -1.55)	<0.0001	1.34 (0.79 to 1.90)	0.002
No. of nurses	0	-2.65 (-3.51 to -1.79)	<0.0001	0.002 (-0.08 to 0.09)	0.96
	1	-0.92 (-1.79 to -0.05)	0.05	-0.13 (-0.21 to -0.04)	0.007
	2	0.45 (-0.40 to 1.30)	0.31	-0.17 (-0.26 to -0.09)	0.0005
No. of scientific, therapeutic and technical staff	0	-2.76 (-3.57 to -1.96)	<0.0001	0.02 (-0.06 to 0.11)	0.60
	1	-2.91 (-3.86 to -1.97)	<0.0001	0.18 (0.07 to 0.28)	0.004
	2	-2.80 (-3.82 to -1.78)	<0.0001	0.31 (0.18 to 0.43)	<0.0001
No. of ambulance staff	0	-2.69 (-3.49 to -1.90)	<0.0001	0.14 (-0.87 to 1.16)	0.79
	1	-2.60 (-3.54 to -1.66)	<0.0001	1.58 (0.36 to 2.80)	0.02
	2	-2.09 (-3.18 to -0.99)	0.001	2.49 (0.99 to 3.99)	0.004
No. of clinical support staff	0	-2.53 (-3.30 to -1.77)	<0.0001	-0.03 (-0.13 to 0.08)	0.63
	1	-1.11 (-1.97 to -0.26)	0.02	-0.15 (-0.27 to -0.04)	0.02
	2	-0.10 (-1.10 to 0.90)	0.85	-0.14 (-0.28 to -0.005)	0.05
No. of infrastructure support staff	0	-2.24 (-3.51 to -0.97)	0.002	-0.05 (-0.19 to 0.09)	0.49
	1	-2.01 (-3.71 to -0.31)	0.03	0.03 (-0.15 to 0.21)	0.76
	2	-2.46 (-4.21 to -0.71)	0.01	0.21 (0.02 to 0.40)	0.04
No. of overnight beds	0	-2.71 (-3.41 to -2.02)	<0.0001	-0.02 (-0.08 to 0.04)	0.522
	1	-2.44 (-3.14 to -1.75)	<0.0001	-0.12 (-0.18 to -0.06)	0.0009
	2	-1.79 (-2.52 to -1.07)	<0.0001	-0.18 (-0.25 to -0.11)	<0.0001
No. of social care staff with accommodation	0	-2.63 (-3.29 to -1.97)	<0.0001	-0.003 (-0.07 to 0.06)	0.94
	1	-2.03 (-2.76 to -1.31)	<0.0001	0.10 (0.03 to 0.18)	0.02
	2	-1.05 (-1.88 to -0.22)	0.02	0.12 (0.02 to 0.21)	0.0002
No. of social care staff without accommodation	0	-2.71 (-3.42 to -1.99)	<0.0001	0.002 (-0.007 to 0.01)	0.61
	1	-2.43 (-3.20 to -1.65)	<0.0001	0.02 (0.006 to 0.03)	0.006
	2	-1.87 (-2.66 to -1.08)	0.0002	0.03 (0.02 to 0.04)	0.0002

[1]Estimates are shown for each corresponding resource in the left-hand column.

For peer review only

Table S9. Changes in care-home deaths per 100,000 persons associated with changes in public expenditure in healthcare (PEH), adjusted for quantity of resources. Associations were evaluated for up to 2 years of interval between real PES per capita and subsequent care-home deaths. Number of observations (sex-years) per analysis is 28.

Resource	Lag (year)	Care-home deaths per 100,000 persons			
		PES per capita (£10)		Resource quantity (thousands)[1]	
		β (95% CI)	p-value	β (95% CI)	p-value
No. of hospital doctors	0	-1.59 (-2.30 to -0.87)	0.0002	2.17 (1.08 to 3.26)	0.003
	1	-1.72 (-2.43 to -1.01)	<0.0001	2.62 (1.51 to 3.73)	0.002
	2	-1.39 (-2.20 to -0.59)	0.003	2.41 (1.11 to 3.71)	0.001
No. of GPs	0	-1.48 (-2.28 to -0.68)	0.001	8.69 (3.41 to 13.97)	0.01
	1	-1.64 (-2.35 to -0.94)	0.0002	10.66 (5.92 to 15.41)	0.0002
	2	-1.37 (-2.05 to -0.69)	0.0008	9.96 (5.35 to 14.57)	0.0004
No. of nurses	0	-0.15 (-0.30 to -0.002)	0.07	-0.35 (-0.54 to -0.17)	0.001
	1	-0.01 (-0.04 to 0.11)	0.83	-0.45 (-0.59 to -0.31)	<0.0001
	2	0.16 (0.04 to 0.28)	0.02	-0.42 (-0.56 to -0.28)	<0.0001
No. of scientific, therapeutic and technical staff	0	-1.53 (-2.42 to -0.64)	0.003	1.87 (0.64 to 3.09)	0.006
	1	-1.58 (-2.45 to -0.71)	0.002	2.12 (0.91 to 3.32)	0.002
	2	-1.30 (-2.15 to -0.45)	0.007	1.96 (0.76 to 3.16)	0.004
No. of ambulance staff	0	-0.37 (-0.78 to 0.04)	0.09	3.17 (-3.39 to 9.72)	0.35
	1	-0.24 (-0.65 to 0.18)	0.28	2.69 (-3.90 to 9.29)	0.43
	2	-0.07 (-0.46 to 0.33)	0.74	2.39 (-3.93 to 8.71)	0.47
No. of clinical support staff	0	-0.15 (-0.33 to 0.02)	0.10	-0.33 (-0.63 to -0.03)	0.04
	1	-0.02 (-0.17 to 0.11)	0.69	-0.55 (-0.78 to -0.31)	0.0002
	2	0.12 (-0.04 to 0.28)	0.16	-0.43 (-0.68 to -0.18)	0.003
No. of infrastructure support staff	0	0.25 (0.004 to 0.50)	0.06	-0.74 (-1.08 to -0.41)	0.0002
	1	0.38 (0.08 to 0.68)	0.02	-0.72 (-1.11 to -0.32)	0.002
	2	0.33 (-0.09 to 0.74)	0.14	-0.37 (-0.89 to 0.15)	0.18
No. of overnight beds	0	-0.84 (-1.12 to -0.52)	<0.0001	-0.76 (-1.09 to -0.42)	0.0002
	1	-0.84 (-1.08 to -0.59)	<0.0001	-0.92 (-1.19 to -0.65)	<0.0001
	2	-0.60 (-0.85 to -0.35)	0.0001	-0.86 (-1.15 to -0.57)	<0.0001
No. of social care staff with accommodation	0	-0.29 (-0.50 to -0.08)	0.01	0.21 (-0.04 to 0.46)	0.11
	1	-0.15 (-0.36 to -0.06)	0.18	0.19 (-0.09 to 0.47)	0.20
	2	-0.02 (-0.18 to -0.23)	0.83	0.13 (-0.14 to 0.41)	0.35
No. of social care staff without accommodation	0	-0.96 (-1.34 to -0.58)	<0.0001	0.13 (0.07 to 0.20)	0.0003
	1	-0.96 (-1.28 to -0.64)	0.0003	0.16 (0.10 to 0.21)	<0.0001
	2	-0.79 (-1.06 to -0.52)	0.0003	0.16 (0.11 to 0.21)	<0.0001

[1]Estimates are shown for each corresponding resource in the left-hand column.

For peer review only

Table S10. Changes in home deaths per 100,000 persons associated with changes in public expenditure in healthcare (PEH), adjusted for quantity of resources. Associations were evaluated for up to 2 years of interval between real PES per capita and subsequent home deaths. Number of observations (sex-years) per analysis is 28.

Resource	Lag (year)	Home deaths per 100,000 persons			
		PES per capita (£10)		Resource quantity (thousands) [1]	
		β (95% CI)	p-value	β (95% CI)	p-value
No. of hospital doctors	0	-0.70 (-1.05 to -0.36)	0.0005	0.89 (0.36 to 1.42)	0.003
	1	-0.75 (-1.06 to -0.44)	0.0001	1.09 (0.61 to 1.58)	0.0002
	2	-0.67 (-0.97 to -0.37)	0.0003	1.11 (0.62 to 1.61)	0.0003
No. of GPs	0	-0.61 (-1.00 to -0.22)	0.005	3.26 (0.69 to 5.83)	0.02
	1	-0.65 (-0.98 to -0.32)	0.0009	4.00 (1.76 to 6.23)	0.002
	2	-0.62 (-0.89 to -0.36)	0.0002	4.37 (2.57 to 6.16)	0.0001
No. of nurses	0	-0.11 (-0.18 to -0.04)	0.006	-0.15 (-0.23 to -0.06)	0.004
	1	-0.03 (-0.10 to 0.02)	0.22	-0.18 (-0.25 to -0.11)	<0.0001
	2	0.04 (-0.01 to 0.10)	0.16	-0.16 (-0.22 to -0.10)	<0.0001
No. of scientific, therapeutic and technical staff	0	-0.59 (-1.02 to -0.15)	0.01	0.64 (0.03 to 1.24)	0.05
	1	-0.64 (-1.03 to -0.25)	0.004	0.81 (0.27 to 1.36)	0.008
	2	-0.59 (-0.93 to -0.25)	0.003	0.86 (0.38 to 1.34)	0.001
No. of ambulance staff	0	-0.14 (-0.34 to 0.05)	0.15	0.25 (-2.79 to 3.29)	0.87
	1	-0.12 (-0.30 to -0.05)	0.19	1.05 (-1.79 to 3.88)	0.48
	2	-0.06 (-0.22 to 0.11)	0.50	1.13 (-1.48 to 3.73)	0.41
No. of clinical support staff	0	-0.11 (-0.19 to -0.03)	0.01	-0.17 (-0.30 to -0.04)	0.02
	1	-0.04 (-0.11 to 0.02)	0.20	-0.23 (-0.33 to -0.12)	0.0003
	2	0.03 (-0.04 to 0.10)	0.48	-0.16 (-0.27 to -0.05)	0.01
No. of infrastructure support staff	0	0.08 (-0.02 to 0.19)	0.14	-0.36 (-0.50 to -0.21)	<0.0001
	1	0.15 (0.02 to 0.27)	0.03	-0.32 (-0.49 to -0.16)	0.0007
	2	0.11 (-0.06 to 0.28)	0.22	-0.15 (-0.36 to 0.07)	0.19
No. of overnight beds	0	-0.40 (-0.55 to -0.25)	<0.0001	-0.32 (-0.48 to -0.16)	0.0007
	1	-0.38 (-0.49 to -0.27)	<0.0001	-0.38 (-0.50 to -0.26)	<0.0001
	2	-0.26 (-0.37 to -0.15)	0.0002	-0.34 (-0.47 to -0.21)	<0.0001
No. of social care staff with accommodation	0	-0.16 (-0.26 to -0.06)	0.004	0.07 (-0.05 to 0.19)	0.25
	1	-0.10 (-0.18 to -0.01)	0.04	0.10 (-0.02 to 0.22)	0.11
	2	-0.02 (-0.10 to 0.06)	0.61	0.09 (-0.02 to 0.20)	0.13
No. of social care staff without accommodation	0	-0.45 (-0.63 to -0.27)	<0.0001	0.06 (0.03 to 0.09)	0.001
	1	-0.40 (-0.56 to -0.25)	<0.0001	0.06 (0.04 to 0.09)	0.0001
	2	-0.31 (-0.45 to -0.18)	0.0001	0.06 (0.04 to 0.08)	<0.0001

[1]Estimates are shown for each corresponding resource in the left-hand column.

For peer review only

Table S11. Changes in care-home and home deaths per 100,000 persons associated with change in no. of nurses. Associations were evaluated for up to 2 years of interval between no. of nurses and subsequent home deaths. Number of observations (sex-years) per analysis is 28.

Place of deaths	Lag (year)	No. of nurses (thousands)	
		β (95% CI)	p-value
Care-home deaths per 100,000 persons	0	-0.38 (-0.57 to -0.19)	0.0007
	1	-0.46 (-0.59 to -0.32)	<0.0001
	2	-0.37 (-0.52 to -0.22)	0.0001
Home deaths per 100,000 persons	0	-0.17 (-0.27 to -0.07)	0.003
	1	-0.18 (-0.25 to -0.12)	<0.0001
	2	-0.15 (-0.21 to -0.08)	0.0002

For peer review only

Table S12. Projected excess deaths prevented in 2015-20 by planned health and social spending under different scenarios of annual productivity increases along with the corresponding additional spending needed to completely close the mortality gap. Numbers in parentheses are 95% confidence intervals.

Scenario	Annual productivity (%)	Excess deaths prevented (%)	Additional spending to prevent 100% of excess deaths (£bn)
**2015**			
Conservative	0	23.58 (19.39 to 27.79)	4.548 (3.647 to 5.84)
Moderate	1	23.82 (19.58 to 28.06)	4.49 (3.6 to 5.76)
Aggressive	3	24.29 (19.96 to 28.62)	4.37 (3.5 to 5.63)
**2016**			
Conservative	0	45.3 (37.24 to 53.39)	4.11 (2.97 to 5.73)
Moderate	1	46.21 (37.99 to 54.48)	3.96 (2.85 to 5.55)
Aggressive	3	48.06 (39.51 to 56.61)	3.68 (2.61 to 5.21)
**2017**			
Conservative	0	51.56 (42.4 to 60.77)	4.4 (3.03 to 6.37)
Moderate	1	53.15 (43.68 to 62.62)	4.13 (2.8 to 6.04)
Aggressive	3	56.38 (46.35 to 66.4)	3.63 (2.37 to 5.43)
**2018**			
Conservative	0	53.56 (44.01 to 63.11)	4.96 (3.35 to 7.28)
Moderate	1	55.72 (45.8 to 65.67)	4.55 (2.99 to 6.77)
Aggressive	3	60.29 (49.54 to 71.05)	3.77 (2.34 to 5.83)
**2019**			
Conservative	0	58.33 (47.94 to 68.71)	5.13 (3.27 to 7.8)
Moderate	1	61.28 (50.38 to 72.24)	4.53 (2.76 to 7.07)
Aggressive	3	67.6 (55.56 to 79.67)	3.44 (1.83 to 5.74)
**2020**			
Conservative	0	54.1 (44.46 to 63.74)	6.41 (4.29 to 9.43)
Moderate	1	57.41 (47.2 to 67.67)	5.6 (3.61 to 8.44)
Aggressive	3	64.58 (53.09 to 76.1)	4.14 (2.37 to 6.67)
Ideal	10.79 (7.8 to 14.46)	100	0

For peer review only

SUPPLEMENTARY FIGURE LEGENDS

Figure S1: Public-sector expenditure on healthcare (PEH) and social care (PES). Data are shown from 2001/02–2014/15. Top plot shows data for PEH whereas bottom plot shows data for PES. Solid lines correspond to total PEH or PES in billions of pounds (left hand *y*-axis). Dotted lines correspond to PEH or PES in pounds per capita (right hand y-axis) calculated from PEH/PES and population size as estimated by the UK's Office for National Statistics (ONS). Blue lines indicate nominal expenditure. Red lines indicate real expenditure adjusted to 2014/15 prices.

Figure S2: Time-trend projections of mortality rates by place of death. Mortality rates (y-axis) per year from 2001 to 2014 are shown for five places of death with 'Other' representing any place of death that doesn't fall into the other four categories (data are unavailable for a breakdown of where these other places might be). The black and blue lines represent actual ASDR for the 2001–10 and 2011–14 periods, respectively. The red line represents predicted ASDR using 2001–10 as an observation base while the 95% CIs are denoted by the beige-colored area.

Figure S3: **Population mortality projections to 2020**. Age-standardised death rates (ASDR) (left hand y-axis) and difference in the number of deaths between actual and predicted rates (right hand y-axis) projected annually to 2020 are shown. Black lines indicate 2009–14 base-fitted (black solid line) and 2015–20 base-forecasted (black dotted line) values. Red lines indicate the same using an observation base for the years 2001–2010. The bisque-coloured area denotes the 95% CIs for the model for 2009–14 data, and the pink area, the 95% CIs for the model for 2001–10 data. The grey bars represent the difference between the number of deaths projected using a 2009–14 observation base and the number forecasted using a 2001–10 observation base, where positive values correspond to excess deaths. Error bars signify 95% CIs. *P < 0.05, **P < 0.01 and ***P < 0.001.

For peer review only

Figure S1

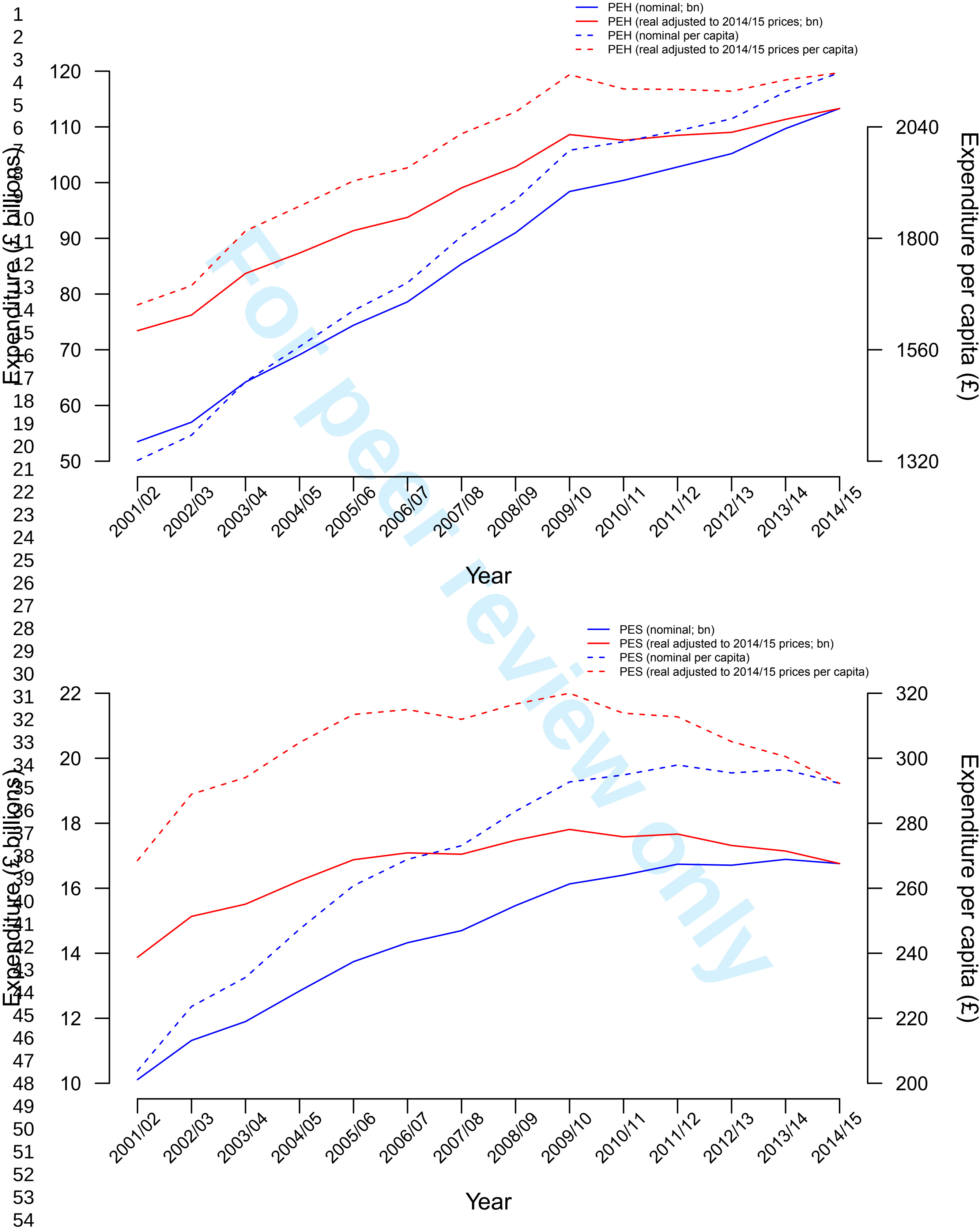

For peer review only

Figure S2

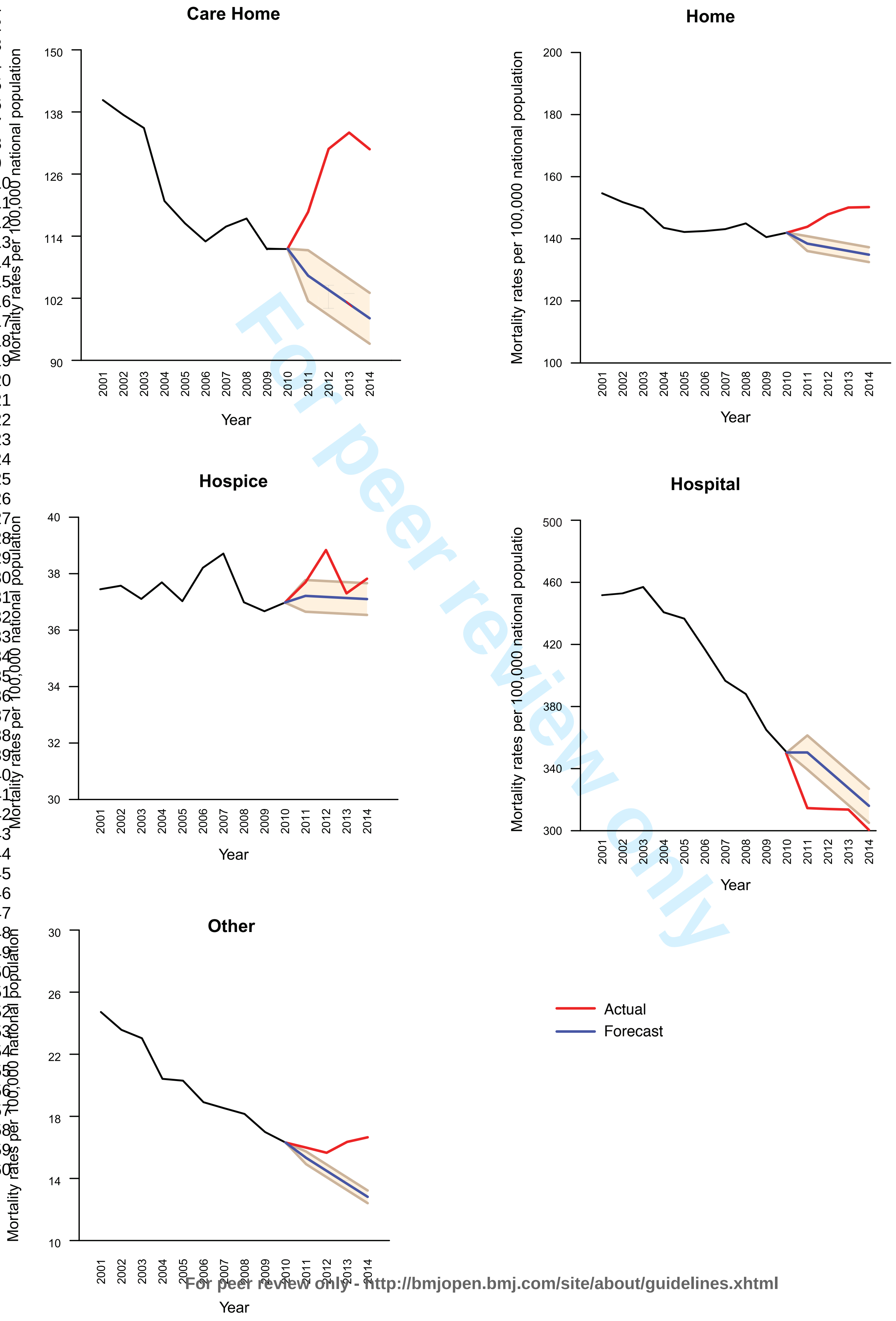

For peer review only

BMJ Open

Figure S3

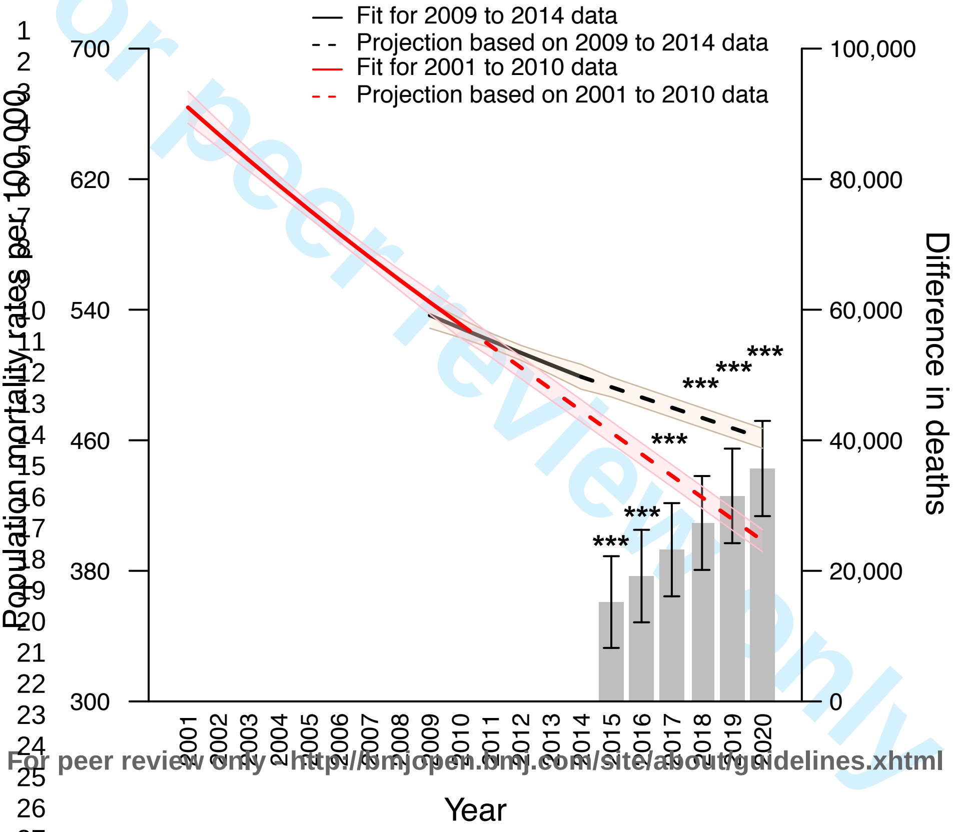

For peer review only

SUPPLEMENTARY APPENDIX

Data collection and generation

Age-standardized rates

All-cause age-standardized death rates (ASDR) were calculated using the ONS' standardized mortality rate calculation template with the standard European population.[1] The ten age groups used were: 0-34, 35-49, 50-54, 55-59, 60-64, 65-69, 70-74, 75-79, 80-84, and ≥85.

Age-standardized potential years of life lost rates were calculated using the same age groups and by weighting the number of 'amenable' deaths in a given year by the standard European population and the number of additional years that the deceased might have been expected to live in the presence of timely and effective healthcare.[2]

Spending data

Nominal public expenditure on health (PEH) data were defined as total expenditure limits for the Department of Health (responsible for the NHS in England), for the financial years 2001/02 to 2014/15, and were collated from Her Majesty's Treasury's Public Expenditure Statistical Analyses.[3] To account for inflation, real PEH using 2014/15 financial year prices was calculated as the product of nominal PEH and the gross domestic product (GDP) deflator for a given financial year, divided by 100, which denoted the GDP deflator for the financial year 2014/15. To ease interpretation, real PEH per capita was converted to units of £10. Public expenditure on social care (PES) in nominal terms was defined as total gross adult social care expenditure, which excludes the Supporting People fund (a block grant to local government supporting vulnerable people living in their own homes available from 2003/04) and NHS transfers.[4] To calculate the 2014/15 data point, we used the percentage change in the bridging data between 2013/14 and 2014/15. From this, we calculated real PEH per capita in units of £10.

Planned spending from 2015/16 to 2020/21 for healthcare was sourced from the 2015 Spending Review and Autumn Statement.[5] For adult social care spending, no consolidated planned expenditure limit was available. Therefore, we defined 2015/16 to 2020/21 planned real PES based on the continuation of –2.25% change in core PES in 2014/15 supplemented by the potential

For peer review only

revenue from the adult social care precept, which is intended to raise funds through 2% per annum increases in local government tax revenues.[6]

Deprivation data

Indices of Multiple Deprivation (IMD), a composite measure using data on topics such as employment, education, and crime, were provided for each local government area by the UK Department for Communities and Local Government for 2010[7]. Population-weighted deprivation status was provided as the average rank of the ranks of the five IMD variables, ranging from 1 to 325, in order of decreasing deprivation.

Resource data

Health and social care staff and bed numbers (resource data) available for 2001 to 2014 were obtained for the following: the number of NHS hospital doctors; general practitioners (GPs); qualified NHS health and social care nurses; qualified NHS scientific, therapeutic and technical staff; NHS ambulance staff; NHS health and social care clinical support staff; NHS infrastructure support staff[8,9]; the number of overnight beds in NHS hospitals[10]; and the numbers of staff providing social care with accommodation and without (see Supplementary Appendix)[11,12].

Employment data in social work activities with accommodation (e.g. care homes) and without accommodation provision (e.g. home care) in England between 2001 and 2014 were obtained from the UK labour market statistics provided by the ONS[11,12]. The 2003 version of Standard Industrial Classification (SIC) codes (SIC 2003) were used to identify businesses conducting social work activities with and without accommodation (SIC 2003 code: 8531 and 8532, respectively). Prior to 2009, data was available from the Annual Business Enquiry, whereas the Business Register Employment Survey provided data from 2009 onwards under the SIC 2007 codes. Proportional mapping using ONS-published proportions between SIC 2003 and SIC 2007 was conducted to estimate the number of employment which corresponds to the aforementioned SIC 2003 codes between 2009 and 2014.[13]

For peer review only

Time-trend prediction analysis

Mortality and potential years of life lost

Given that consistent mortality data were available from 2001 onwards, and that the reductions in real PEH and PES occurred in 2010/11, we used 2001–2010 as an observation base on which to build our time-trend model for predicting mortality rates in 2011–2014.

Depending on whether overdispersion was observed (where the variance exceeds the mean), we resolved to use either a Poisson or quasi-Poisson regression model, the latter of which incorporates a scaling parameter in order to address overdispersion. In every dataset for which we conducted time-series modelling, the variance of the mortality rates exceeded the mean rate. Therefore, we employed a quasi-Poisson model in all analyses, with the logarithmic link function preventing the prediction of negative rates. The model takes the following form:

$$E(M_t) = \exp(\alpha + \beta t), \qquad (1)$$

where $E(M_t)$ is the estimated mortality rate for year t, α is the baseline mortality rate, and β is the estimated drift parameter. For increasing observation-base trends, the following non-linear prediction model proposed by Dyba and colleagues,[14] also assuming a Poisson or quasi-Poisson distribution, was used:

$$E(M_t) = \alpha(1 + \beta t). \qquad (2)$$

The trend of a mortality rate was determined to be decreasing or increasing according to whether the geometric mean of the ratio of the annual percentage change was negative or positive, respectively.

Rate ratios for the observed and expected mortality rates were calculated. We also estimated the number of “excess” (higher-than-expected) or “prevented” (lower-than-expected) deaths in 2011, 2012, 2013 and 2014 by finding the difference between the observed number of deaths and the number of deaths predicted by the 2001–2010-based time-series models above. 95% confidence intervals were computed using the standard errors of the fitted mortality rate estimates.

For peer review only

We ran separate analyses using the above model for males, females, and both sexes. The age-stratified time-trend analyses examined 10 age groups. The place-specific mortality rates used the same approach but were split into just two age groups, those 60 and over, and those less than 60 in addition to an all-age group analysis.

For the analyses of potential years of life lost, the same process was repeated but using the directly standardized rates of potential years of life lost as the outcome instead of mortality rates.

Life expectancy

A univariate autoregressive integrated moving average (ARIMA) model was fitted to life expectancy data available from 1998 to 2010, and used to predict data points for 2011 and 2012 as per a method by Torri and Vaupel[15]. In order to optimize the p, d, and q parameters, we conducted a model search and selected the parameterized model that minimized the Akaike Information Criterion (AIC) value, which is defined as:

$$AIC = 2k - 2\ln(L) \quad (3)$$

where k is 3, i.e. the number of parameters in the ARIMA model, and L is the maximized value of the likelihood function for the model. A drift term was included to account for the clear upward trend in life expectancy over the 1998 to 2010 period.

Aggregating results across years

Aggregate central estimates of deaths across time-trend analyses of each projected year were calculated by addition. For 95% confidence intervals, the root-sum-of-squares method was used:

$$d_{agg_{CI}} = d_{agg} \pm \sqrt{\textstyle\sum_t^n (d_t - d_{t_{CI}})^2} \quad (4)$$

where $d_{agg_{CI}}$ is the aggregate confidence intervals (lower and upper), d_{agg} is the aggregate central estimate, n is the final year in the projected time series, d_t is the central estimate for year t, and $d_{t_{CI}}$ is the confidence interval (lower or upper) for the year t.

Panel-based regression analysis

For peer review only

Model selection

To assess the suitability of different regression approaches, we separately fitted pooled ordinary least squares (OLS), fixed-effects, and random-effects models to the real PEH per capita and population mortality data with sex as the time-invariant entity. The assumption of the random-effects model that the error terms for unobserved or observed time-invariant variables such as sex are uncorrelated with the independent variables means that sex is therefore also able to serve as an independent variable. In fixed-effects models, this is not the case.

We therefore tested whether the consistent but inefficient fixed-effects model was more suitable than the potentially inconsistent but efficient pooled OLS and random-effects approaches by conducting Hausman tests, both of which were highly significant (P<0.001), leading us to reject the random-effects and pooled OLS approaches and their assumptions in favour of a fixed-effects model.

Regression model

We therefore used the following fixed-effects regression model:

$$H_{it} = \beta_0 + \beta_1 X_{1it} + \ldots + \beta_k X_{kit} + U_{it} \quad (5)$$

where H_{it} is the response variable for which i is sex and t is time in years; X_{kit} represents the independent variables; β_k is the coefficient for the independent variables; and U_{it} is the error term. For population-based mortality, H was all-cause ASDR. For the regression analyses in which economic and health resource indicators were not controlled, X was just real PEH or real PES per capita in units of ten pounds sterling. To test whether our results were robust to variations in the economy, we added average annual consumer price index (CPI)[16] and unemployment rate[17] as independent variables to the model. For mediation analyses, each health and social care resource was added as an independent variable.[8–12]

Lag analyses

We conducted 1- and 2-year lag analyses. For an ith year lag analysis, regression was performed using the mortality rate in year x and the value of the explanatory variables (real PEH or real PES per capita, etc.) in year $x - i$.

Mortality projections to 2020

For peer review only

For the 2020 projection analysis, two projections were performed each with a different observation base: 2001–10 for one and 2009–14 for the other. Data points for 2009 and 2010 were included for the second observation base to give at least six observations from which to make predictions.Instead of comparing actual rates with trend-determined expected rates, the projected rates from[14] using these two different observation bases were compared against each other to calculate rate ratios, and the number of excess deaths annually between 2015 and 2020. Mortality rates for 2015 to 2020 were standardized against the National Population 2012-based projections.[18]

Scenario modelling

For the 2020 projection analysis, we assumed that the mortality projections were underpinned by flat real PEH and PES per capita and no change in efficiency since 2014/15. We performed scenario modeling to test how much of the mortality gap could be closed by planned spending under varying assumptions of annual efficiency changes.

To do this, we first performed fixed-effects regression as before but instead using all-cause population ASDR as the outcome variable, and combined real PEH or PES per capita as the explanatory variable. From these analyses, an increase of £10 per capita real spending on health and social care is associated with 2.56 (2.10 to 3.01) lives saved per 100,000.

To calculate the number of lives saved, l, for year t, which could be any year between 2015 to 2020, inclusive, we used the following formula:

$$l_t = (1 + p)^{t-2014} \times \frac{r}{100{,}000} \times \frac{(S_t - S_{2014})}{10} \quad (6)$$

where p is the assumed annual efficiency change; r is the coefficient (or upper or lower bound of the 95% confidence interval); 100,000 is that the coefficient is for all-cause ASDR per 100,000; S_t represents planned health and social care spending combined for year t and S_{2014} denotes the combined health and social care spending outturn for 2014/15. The difference in spending is divided by 10 to acknowledge that the regression coefficient was obtained with units of £10 changes per capita.

For peer review only

The percentage of lives saved was calculated using the number of excess deaths from the mortality projections for year *t*.

The additional spending needed to completely close the gap, i.e. make sure that 100% of lives were saved for year *t*, was computed as follows:

$$S_{additional} = \frac{d_t \times 1{,}000{,}000}{(1+p)^{t-2014} \times r} - S_t + S_{2014} \quad (7)$$

where d_t is the excess deaths calculated for *t*.

For each year between 2015 and 2020, we modeled three different scenarios, each assuming different values for *p*:

1) a conservative 0% annual efficiency change;
2) a moderate 1% annual efficiency gain (1% was the average efficiency to the nearest integer for the period 2001 to 2013 [2014 data were unavailable]); and
3) an aggressive 3% annual efficiency gain (3% was the highest efficiency to the nearest integer reached in the 2001 to 2013 period.

These productivities were defined by using historical and targeted efficiency changes in healthcare as a guide.[19]

In addition, we asked what annual efficiency gain up to 2020 would be needed if there was no spending on top of the planned PEH and PES budgets. This was calculated as follows:

$$p = \left(\frac{d_{2020} \times 1{,}000{,}000}{r \times (S_{2020} - S_{2014})}\right)^{1/(2020-2014)} - 1 \quad (8)$$

REFERENCES

1. Office for National Statistics. Mortality Statistics: Metadata. Newport: ONS; 2015.
2. UK Health and Social Care Information Centre. NHS Outcomes Framework Indicators. May 2015. 2015;Available from: http://content.digital.nhs.uk/nhsof
3. HM Treasury. Public Expenditure Statistical Analyses 2015. London: Williams Lea Group;

2015.

4. NHS Digital. Personal Social Services: Expenditure and Unit Costs, England. 2016;Available from: https://www.gov.uk/government/statistics/personal-social-services-expenditure-and-unit-costs-england-2015-to-2016
5. UK Parliament. Impact of the Spending Review on health and social care. 2016;Available from: https://www.publications.parliament.uk/pa/cm201617/cmselect/cmhealth/139/13902.htm?utm_source=139&utm_medium=fullbullet&utm_campaign=Modulereports
6. UK Parliament. The impact of the Spending Review on health and social care finances. 2016;Available from: https://www.publications.parliament.uk/pa/cm201617/cmselect/cmhealth/139/13906.htm
7. UK Department for Communities and Local Government. English Indices of Deprivation. December 2012. 2012;Available from: https://www.gov.uk/government/collections/english-indices-of-deprivation
8. The NHS Information Centre. NHS workforce: summary of staff in the NHS: results from September 2011 Census. 2012;1–31. Available from: https://catalogue.ic.nhs.uk/publications/workforce/numbers/nhs-staf-2001-2011-medi-dent/nhs-staf-2001-2011-medi-dent-work-rep.pdf
9. Health and Social Care Information Centre. NHS workforce statistics in England, summary of staff in the NHS - 2004-2014. 2015.
10. NHS England. Overnight bed availability and occupancy data. London: 2014.
11. Office for National Statistics. Business Register Employment Survey, 2009-2014. 2014;Available from: https://www.nomisweb.co.uk/
12. Office for National Statistics. Annual Business Enquiry, 2001-2008. 2008;Available from: https://www.nomisweb.co.uk/
13. Office for National Statistics. Correlation between UK SIC 2003 and UK SIC 2007. 2015;Available from: http://webarchive.nationalarchives.gov.uk/20160105160709/http://www.ons.gov.uk/ons/guide-method/classifications/archived-standard-classifications/uk-standard-industrial-classification-1992--sic92-/index.html
14. Dyba T, Hakulinen T, Päivärinta L. A simple non-linear model in incidence prediction. Stat Med 1997;16(20):2297–309.
15. Torri T, Vaupel JW. Forecasting life expectancy in an international context. Int J Forecast 2012;28:519–31.
16. Office for National Statistics. Consumer Price Inflation, release. 2014;Available from: http://webarchive.nationalarchives.gov.uk/20160105160709/http://ons.gov.uk/ons/rel/cpi/consumer-price-indices/december-2014/index.html
17. Office for National Statistics. Unemployment in England, 1992-2015. 2015;https://www.nomisweb.co.uk/reports/lmp/gor/2092957. Available from: https://www.ons.gov.uk/employmentandlabourmarket/peoplenotinwork/unemployment
18. Office for National Statistics. Time series: England population mid-year estimate. 2015;
19. NHS England. NHS Five Year Forward View. Recap briefing for the Health Select Committee on technical modelling and scenarios. 2016.

For peer review only

CHEERS Checklist
Items to include when reporting economic evaluations of health interventions

The **ISPOR CHEERS Task Force Report**, *Consolidated Health Economic Evaluation Reporting Standards (CHEERS)—Explanation and Elaboration: A Report of the ISPOR Health Economic Evaluations Publication Guidelines Good Reporting Practices Task Force*, provides examples and further discussion of the 24-item CHEERS Checklist and the CHEERS Statement. It may be accessed via the *Value in Health* or via the ISPOR Health Economic Evaluation Publication Guidelines – CHEERS: Good Reporting Practices webpage: http://www.ispor.org/TaskForces/EconomicPubGuidelines.asp

Section/item	Item No	Recommendation	Reported on page No/ line No
**Title and abstract**			
Title	1	Identify the study as an economic evaluation or use more specific terms such as "cost-effectiveness analysis", and describe the interventions compared.	
Abstract	2	Provide a structured summary of objectives, perspective, setting, methods (including study design and inputs), results (including base case and uncertainty analyses), and conclusions.	
**Introduction**			
Background and objectives	3	Provide an explicit statement of the broader context for the study. Present the study question and its relevance for health policy or practice decisions.	
**Methods**			
Target population and subgroups	4	Describe characteristics of the base case population and subgroups analysed, including why they were chosen.	
Setting and location	5	State relevant aspects of the system(s) in which the decision(s) need(s) to be made.	
Study perspective	6	Describe the perspective of the study and relate this to the costs being evaluated.	
Comparators	7	Describe the interventions or strategies being compared and state why they were chosen.	
Time horizon	8	State the time horizon(s) over which costs and consequences are being evaluated and say why appropriate.	
Discount rate	9	Report the choice of discount rate(s) used for costs and outcomes and say why appropriate.	
Choice of health outcomes	10	Describe what outcomes were used as the measure(s) of benefit in the evaluation and their relevance for the type of analysis performed.	
Measurement of effectiveness	11a	*Single study-based estimates:* Describe fully the design features of the single effectiveness study and why the single study was a sufficient source of clinical effectiveness data.	

For peer review only

	11b	*Synthesis-based estimates:* Describe fully the methods used for identification of included studies and synthesis of clinical effectiveness data.	
Measurement and valuation of preference based outcomes	12	If applicable, describe the population and methods used to elicit preferences for outcomes.	
Estimating resources and costs	13a	*Single study-based economic evaluation:* Describe approaches used to estimate resource use associated with the alternative interventions. Describe primary or secondary research methods for valuing each resource item in terms of its unit cost. Describe any adjustments made to approximate to opportunity costs.	
	13b	*Model-based economic evaluation:* Describe approaches and data sources used to estimate resource use associated with model health states. Describe primary or secondary research methods for valuing each resource item in terms of its unit cost. Describe any adjustments made to approximate to opportunity costs.	
Currency, price date, and conversion	14	Report the dates of the estimated resource quantities and unit costs. Describe methods for adjusting estimated unit costs to the year of reported costs if necessary. Describe methods for converting costs into a common currency base and the exchange rate.	
Choice of model	15	Describe and give reasons for the specific type of decision-analytical model used. Providing a figure to show model structure is strongly recommended.	
Assumptions	16	Describe all structural or other assumptions underpinning the decision-analytical model.	
Analytical methods	17	Describe all analytical methods supporting the evaluation. This could include methods for dealing with skewed, missing, or censored data; extrapolation methods; methods for pooling data; approaches to validate or make adjustments (such as half cycle corrections) to a model; and methods for handling population heterogeneity and uncertainty.	
**Results**			
Study parameters	18	Report the values, ranges, references, and, if used, probability distributions for all parameters. Report reasons or sources for distributions used to represent uncertainty where appropriate. Providing a table to show the input values is strongly recommended.	
Incremental costs and outcomes	19	For each intervention, report mean values for the main categories of estimated costs and outcomes of interest, as well as mean differences between the comparator groups. If applicable, report incremental cost-effectiveness ratios.	
Characterising uncertainty	20a	*Single study-based economic evaluation:* Describe the effects of sampling uncertainty for the estimated incremental cost and incremental effectiveness parameters, together with the impact	

		of methodological assumptions (such as discount rate, study perspective).	
	20b	*Model-based economic evaluation:* Describe the effects on the results of uncertainty for all input parameters, and uncertainty related to the structure of the model and assumptions.	
Characterising heterogeneity	21	If applicable, report differences in costs, outcomes, or cost-effectiveness that can be explained by variations between subgroups of patients with different baseline characteristics or other observed variability in effects that are not reducible by more information.	
**Discussion**			
Study findings, limitations, generalisability, and current knowledge	22	Summarise key study findings and describe how they support the conclusions reached. Discuss limitations and the generalisability of the findings and how the findings fit with current knowledge.	
**Other**			
Source of funding	23	Describe how the study was funded and the role of the funder in the identification, design, conduct, and reporting of the analysis. Describe other non-monetary sources of support.	
Conflicts of interest	24	Describe any potential for conflict of interest of study contributors in accordance with journal policy. In the absence of a journal policy, we recommend authors comply with International Committee of Medical Journal Editors recommendations.	

For consistency, the CHEERS Statement checklist format is based on the format of the CONSORT statement checklist

The **ISPOR CHEERS Task Force Report** provides examples and further discussion of the 24-item CHEERS Checklist and the CHEERS Statement. It may be accessed via the *Value in Health* link or via the ISPOR Health Economic Evaluation Publication Guidelines – CHEERS: Good Reporting Practices webpage: http://www.ispor.org/TaskForces/EconomicPubGuidelines.asp

The citation for the CHEERS Task Force Report is:
Husereau D, Drummond M, Petrou S, et al. Consolidated health economic evaluation reporting standards (CHEERS)—Explanation and elaboration: A report of the ISPOR health economic evaluations publication guidelines good reporting practices task force. Value Health 2013;16:231-50.

For peer review only

BMJ Open

Effects of health and social care spending constraints on mortality in England: A time trend analysis

Journal:	*BMJ Open*
Manuscript ID	bmjopen-2017-017722.R1
Article Type:	Research
Date Submitted by the Author:	10-Aug-2017
Complete List of Authors:	Watkins, Johnathan; PILAR Research and Education<br>Wulaningsih, Wahyu<br>Da Zhou, Charlie<br>Marshall, Dominic<br>Sylianteng, Guia<br>Dela Rosa, Phyllis<br>Miguel, Viveka<br>Raine, Rosalind<br>King, Lawrence; Cambridge University<br>Maruthappu, Mahiben
**Primary Subject Heading**:	Health economics
Secondary Subject Heading:	Epidemiology, Health policy, Health services research, Public health
Keywords:	HEALTH ECONOMICS, expenditure, mortality, social care, life expectancy

SCHOLARONE™ Manuscripts

Effects of health and social care spending constraints on mortality in England: A time trend analysis

Johnathan Watkins[1,2]*†, Wahyu Wulaningsih[2,3]*, Charlie Da Zhou[4], Dominic C. Marshall[5], Guia D.C Sylianteng[2,6], Phyllis G. Dela Rosa[2,7], Viveka A. Miguel[2,8], Rosalind Raine[9], Lawrence P. King[10], Mahiben Maruthappu[9]†

[1] Institute for Mathematical & Molecular Biomedicine, King's College London, London, UK

[2] PILAR Research and Education, Cambridge, UK

[3] MRC Unit for Lifelong Health and Ageing, University College London, London, UK

[4] Medical Sciences Division, University of Oxford, Oxford, UK

[5] Oxford University Clinical Academic Graduate School, John Radcliffe Hospital, Oxford, UK

[6] London School of Hygiene and Tropical Medicine, London, UK

[7] University of the Philippines Manila, Metro Manila, Philippines

[8] University of the Philippines Diliman, Quezon City, Metro Manila, Philippines

[9] Department of Applied Health Research, University College London, London, UK

[10] Department of Sociology, University of Cambridge, Cambridge, UK

*** These authors contributed equally**

† Corresponding authors:
Dr. Mahiben Maruthappu
Department of Applied Health Research,
1–19 Torrington Place, University College London, WC1E 7HB, UK
Email: m.maruthappu@gmail.com

Dr. Johnathan Watkins
PILAR Research and Education
20 Station Road, Cambridge, CB1 2JD, UK
Email: jwatkins@pilar.org.uk

Word count: abstract (250), manuscript (2,851)

Number of references: 22

Short title: Health and social care spending and mortality

For peer review only - http://bmjopen.bmj.com/site/about/guidelines.xhtml

For peer review only

ABSTRACT

Objective

Since 2010, England has experienced relative constraints in public expenditure on healthcare (PEH) and social care (PES). We sought whether these constraints have affected mortality rates.

Methods

We collected data on health and social care resources and finances for England from 2001 to 2014. Time-trend analyses were conducted to compare the actual mortality rates in 2011 to 2014 with the counterfactual rates expected based on trends before the spending constraints. Fixed-effects regression analyses were conducted using annual data on PES and PEH with mortality as the outcome, with further adjustments for macroeconomic factors and resources. Analyses were stratified by age group, place of death, and lower-tier local authority (N=325). Mortality rates to 2020 were projected based on recent trends.

Results

Spending constraints between 2010–2014 were associated with an estimated 45,368 (95% confidence interval (CI): 34,530–56,206) higher-than-expected number of deaths compared to pre-2010 trends. Deaths in those aged ≥60 and in care homes accounted for the majority. PES was more strongly linked with care-home and home mortality than PEH, with each £10 per capita decline in real PES associated with an increase of 5.10 (3.65–6.54) (P<0.001) care-home deaths per 100,000. These associations persisted in lag analyses and after adjustment for macroeconomic factors. Furthermore, we found that changes in real PES per capita, may be linked to mortality primarily via changes in nurse numbers. Projections to 2020 suggested that planned health and social care

For peer review only

spending would not close the gap and is cumulatively linked to an estimated 152,141 (95% CI: 134,597 and 169,685) additional deaths.

Conclusions

Spending constraints, especially PES, are associated with a substantial mortality gap. We suggest that spending should be targeted on improving care delivered in care homes and at home; and maintaining or increasing nurse numbers.

KEYWORDS

Health care, social care, spending, expenditure, mortality, PYLL, life expectancy, time trend

STRENGTHS AND LIMITATIONS

- Time trend analysis with projections of future outcomes and counterfactual analysis
- Sensitivity analyses by using other outcomes: potential years of life lost and life expectancy
- Analysis included potential effect modifiers and mediating factors
- Variations may exist at the local level which were not identified by the study
- There may be other mediating factors beyond those explored in this analysis

For peer review only

For peer review only - http://bmjopen.bmj.com/site/about/guidelines.xhtml

INTRODUCTION

Health systems in most industrialised countries are facing the concurrent challenge of managing rising deman amidst funding constraints, following the recent global economic crisis. The National Health Service (NHS) in England, which provides tax-funded, universal health coverage, is no exception.[1–4] Since 2010, the NHS in England has seen a real-term annual increase in public healthcare spending in England of 1.30% between 2010 and 2014, as compared to historical annual growth of around 4%.[4] During the same period, demand and healthcare cost inflation have increased, with a growing and ageing population, in addition to new treatments and technologies. By 2020/21, a funding gap, between what is needed and what is available, has been predicted, unless major changes are implemented.[4]

Although the role of social determinants in health is increasingly acknowledged,[5] there is underinvestment in social care in many high-income countries such as the United States.[6] In England, public-sector funding for social care has also suffered.[7] Such funds enable the provision of means-tested home care and care-home accommodation,[8] allowing, for example, hospitals to discharge frail patients who would otherwise have no adequate support. Real-term adult social care spending decreased by 1.19% annually between 2010 and 2014 after correcting for the effect of inflation, reversing the annual increase of 3.17% between 2001 and 2009. This is despite increasing demand, with the group most likely to require social care – the over-85s – set to rise from 1.6 million in 2015 to 1.8 million in 2020.[9]

For peer review only

For peer review only - http://bmjopen.bmj.com/site/about/guidelines.xhtml

This supply-demand mismatch has manifested in several ways. During the first week of 2017, more than four in 10 NHS hospitals declared a major alert.[10] Emergency medicine departments (A&E) saw 900,000 (4.6%) more attendances in 2015/16 compared with the previous year, and 4% more emergency hospital admissions.[11] Over the past two years, the number of elderly patients waiting over 12 hours in A&E has trebled, and there has been a 31% increase in delayed hospital discharges. [11]

While the funding gaps facing health and social care have been well-quantified,[12,13] the impact on population outcomes remains unclear. Here we sought to model the past, present, and future impact of funding constraints experienced by the publicly financed health & social care system in England on mortality, to provide insights into the association between funding and health outcomes and inform future financial allocations.

METHODS

Data collection

Population mortality, PYLL and life expectancy

Annual population mortality data for England were extracted from the UK's Office for National Statistics (ONS)[14] based on Medical Certificates of Cause of Death from the Registration Online system. Age-standardized death rates (ASDR) were calculated with reference to a standard European population using mortality data split into ten age groups (see Supplementary Appendix). Information on deaths by place of occurrence (care homes, hospice, home, hospital, and other

For peer review only - http://bmjopen.bmj.com/site/about/guidelines.xhtml

establishments) was provided by Public Health England. Additionally, mortality data were collected for 325 lower-tier local authorities (LAs), which are the equal of districts, boroughs or city councils, based on 2010 boundaries with Cornwall and the Isles of Scilly combined, due to the small population of the latter. Data on life expectancy were obtained from the ONS, whereas data on potential years of life lost (PYLL), a measure of premature mortality from causes considered amenable to healthcare, were obtained from the UK Health and Social Care Information Centre[15] and age-standardized

Spending and resources data

Nominal public expenditure on health (PEH) data were defined as total expenditure limits for the Department of Health (responsible for the NHS in England), for the financial years 2001/2002 to 2014/2015, and were collated from Her Majesty's Treasury's Public Expenditure Statistical Analyses.[16] To account for inflation, real PEH using 2014/2015 financial year prices was calculated as the product of nominal PEH and the gross domestic product (GDP) deflator for a given financial year, divided by 100, which denoted the GDP deflator for the financial year 2014/2015. To ease interpretation, real PEH per capita was converted to units of £10. Public expenditure on social care (PES) in nominal terms was defined as total gross adult social care expenditure, which excludes the Supporting People fund (a block grant to local government supporting vulnerable people living in their own homes available from 2003/2004) and NHS transfers.[17].Further details, including health and social care resources (staff and bed numbers), are provided in the Supplementary Appendix.

Statistical analysis

For peer review only - http://bmjopen.bmj.com/site/about/guidelines.xhtml

For all analyses, we used 2001 as the start of the study period since complete population mortality data were available from this year onwards. To assess mortality gap, time-trend analyses were conducted using Poisson or quasi-Poisson regression models with all-cause ASDR as the dependent variable and calendar year as the independent variable. Analyses were stratified by sex and repeated using rates of PYLL and life expectancy as alternative health outcomes. Analysis for mortality outcomes was further stratified by age groups and place of death as well as their combination, and by local government area. Further details are provided in the Supplementary Appendix.

Fixed-effects regression models were conducted using real PEH/PES per capita and controls for economic variation (unemployment[18] and the average annual consumer price index [CPI][19]) and average weekly pensions as the independent variables, and care home, home and hospital mortality as the dependent variables. For a given year, population mortality on the same year was the outcome whereas spending data for a financial year starting at the given year was used as the main predictor. Since population mortality was collected annually and spending data is reported for each financial year (starting 1 April in the UK), we repeated our analysis using 1- and 2-year lag periods. We additionally incorporated both PEH and PES in the same model.

Effects of public spending on resources such as staff or infrastructure have been documented[20] and these resources have also been linked with health outcomes.[21] Therefore, we explored resources of health and social care as potential mediating factors by running fixed-effects regression models with real PEH/PES per capita and each resource variable as the independent variables; and care home/home mortality as the dependent variable. Resource variables that were associated with the dependent variable, resulted in weaker associations between PEH/PES and the dependent variable,

For peer review only - http://bmjopen.bmj.com/site/about/guidelines.xhtml

and retained the same coefficient sign as univariable regressions were considered to be putative mediators. Analyses were repeated for 1- and 2-year lag periods.

To investigate the possibility of a future population mortality gap, two different mortality projection analyses for 2015 to 2020 were performed. Each analysis had a different observation base: one using 2001 to 2010 data and the other using 2009 to 2014 data, with the number of projected deaths based on 2001 to 2010 data subtracted from the number of projected deaths based 2009 to 2014 data.

Finally, using fixed-effects regression modeling, the total health and social care spending needed to close the mortality gap on top of planned spending budgets, as of 2016, were computed for three different efficiency scenarios: 0%, 1% and 3% (see Supplementary Appendix for a definition and explanation).[22]

RESULTS

Health and social care spending

From 2001/02 to 2009/10, the average annual increase in real PEH per capita was 3.82%. Between 2010/11 and 2014/15, the average annual increase was 0.41% (Figure S1). Planned real PEH spending to 2020/21 is forecasted to increase at an average of 0.72% per year, based on published information up to 2016. Real PES per capita experienced an average annual increase of 2.20% between 2001/02 and 2009/10, while between 2010/11 and 2014/15 it decreased by 1.57%.

For peer review only - http://bmjopen.bmj.com/site/about/guidelines.xhtml

Assessment of a mortality gap

From 2001 to 2010, the absolute number of deaths in England decreased by an average of 0.77% per year. From 2011 to 2014, the number of deaths increased by an average of 0.87% per year. To quantify the potential mortality gap associated with the 2010 spending constraints, we compared actual and predicted mortality rates for both sexes and found 8,148 higher-than-expected number of deaths in 2012 (95% confidence interval 2,004–14,292), 18,896 (12,641–25,152) in 2013, and 18,324 (11,953–24,695) in 2014 (Figure 1). Sex-stratified time-series analyses revealed similar results between males and females (Table S1).

To validate these results, we looked at two alternative health outcomes: life expectancy and PYLL. We found 2012–14 male and female life expectancy to be 3.84 months (2.40–5.28) and 5.16 months (3.36–7.08) less, respectively, than the values anticipated from 2001–03 to 2009–11 data (Table S2). Moreover, we found that PYLL increased after the 2009/10 spending constraints compared with predicted rates (Table S3).

To see whether the possible effects of the 2010 spending constraints were sensitive to age, we conducted time-series analyses for ten different age groups (Table S4). Higher-than-expected numbers of deaths were confined to those ≥60 years of age with all six ≥60 age groups showing excess deaths in 2013. By contrast, <60 age groups most often exhibited fewer deaths.

We next looked at place of death for all age groups, while stratifying them by age into <60 and those ≥60 years. Time-trend analyses revealed care-home and home deaths to be the first and second largest contributors, respectively, to excess deaths across age groups and in those ≥60

For peer review only - http://bmjopen.bmj.com/site/about/guidelines.xhtml

(Figure 2, Figure S2 and Table S5), with a relative increase in these places of death over others during the study period. For every year analysed, lower-than-expected numbers of deaths occurred in hospitals. For those <60 years, hospitals were the most frequent place of death, and so the net result was a lower-than-expected number of <60 deaths. Time-series analyses by local government area (for time points up to 2013) revealed no correlation between changes in the number of expected deaths and deprivation (P=0.14, R_S=0.08).

Longitudinal associations between spending and mortality

We found that £10 per capita declines in real PEH and real PES were associated with increases of 0.19 (0.01–0.38) (P=0.049) and 5.10 (3.65–6.54) (P<0.001) care-home deaths per 100,000 in England, respectively (Table 1). For PES, these relationships persisted at least two years after the initial change but became weaker for PEH. We next sought to demonstrate that these associations were independent of macroeconomic forces such as unemployment (for younger age groups) and pensions (for older age groups), which have each been linked with increased mortality.[23,24] On adjusting the regression models for macroeconomic factors and the annual average of weekly pensions, we found real PES per capita and real PEH per capita to remain inversely associated with care-home mortality (Table 1). Next, we used a regression model with both real PES and PEH per capita as explanatory variables and found only PES remained inversely associated with care-home mortality (Table 1). In this model, the variance inflation factor between PEH and PES was 4.15, suggesting that multicollinearity was not a problem. The results for home deaths replicated those for care-home deaths (Table S6). In contrast to care-home and home mortality, real PEH per capita was more strongly related to hospital mortality than real PES per capita without adjusting for controls.

For peer review only - http://bmjopen.bmj.com/site/about/guidelines.xhtml

On adjusting for macroeconomic forces, the relationship between PEH and hospital mortality was no longer apparent (Table S7).

Identification of resources potentially mediating the spending-mortality relationship

We found that the numbers of NHS hospital and community nurses, and NHS health and social care clinical support staff were each associated with care-home mortality. These factors alleviated the relationship between real PES per capita and care-home mortality at lag years 1 and 2, suggesting these staff numbers were important mediators of the spending-mortality relationship (Table 2). We found the number of nurses, but not clinical support staff, was suggested to be a mediator of the relationship between spending (both PEH and PES) and home mortality rates for lag years 1 and 2 (Table S8). We also found the number of nurses to be the sole mediator of the relationships between PEH per capita and care-home mortality (Table S9), and PEH per capita and home mortality (Table S10) for all years. From 2001 to 2010, the average annual increase in nurse numbers was 1.61% whereas from 2010 to 2014, the average annual increase was over twenty-fold lower at 0.07% (Table S11).

Mortality projections to 2020 and scenario modelling

We projected mortality rates to 2020 using two observation bases: one using 2001–10 data and a second using 2009–14 (2009 and 2010 were included to ensure a sufficiently large observation base). We found that the 2009–14-based projection entailed an additional 152,141 deaths (aggregate

For peer review only - http://bmjopen.bmj.com/site/about/guidelines.xhtml

95% CI: 134,597–169,685) from 2015 to 2020 compared with the 2001–10-based projection (Figure S3).

To determine what it would take to close this gap above planned health and social care spending to 2020/21, we modelled three different scenarios (Figure 3 and Table S12). On top of the combined health and social care budget, as of the end of 2016, the aggregate spending and efficiency combinations required to completely close the mortality gap would be: an additional £29.56 billion (£25.74–33.37 billion) for a conservative 0% annual efficiency increase; an additional £27.26 billion (£23.61–30.91 billion) for a moderate 1% annual efficiency increase, and an additional £23.03 billion (£19.67–26.39 billion) for an aggressive 3% annual efficiency increase (Figure 3 with an annual breakdown in Table S12). Under an ideal scenario in which no additional spending would be needed to close the mortality gap, the annual efficiency increase to 2020 would need to reach 10.79% (7.8 to 14.46). In these scenarios, efficiency gains reflect improvements across the system required to meet respective spending constraints, while avoiding excess mortality.

DISCUSSION

This study demonstrates that recent constraints in PEH and PES spending in England have been associated with nearly 45,000 higher-than-expected numbers of deaths between 2012 and 2014. If these trends continue, even when considering the increased planned funding as of 2016, we estimate approximately 150,000 additional deaths may arise between 2015 and 2020. Combining these projected excess deaths and the observed deaths prior to 2015 translate to around 120,000 excess

For peer review only - http://bmjopen.bmj.com/site/about/guidelines.xhtml

For peer review only

deaths from 2010 to 2017. Contemporaneous reductions in life expectancy and excesses in measures of preventable death both validated our mortality findings.

The excess deaths observed in our study corroborated recent evidence highlighting the reversal of declining mortality trends observed in England and Wales in the past decade.[13] Similar to findings reported by Hiam and colleagues,[13] we observed that those aged ≥60 years are most susceptible to the observed excess mortality burden, as compared to those aged <60 years. We added to this evidence by additionally evaluated potential years of life lost (PYLL) and life expectancy as outcomes, projecting excess deaths to 2020, determining potential mediating factors which may explain the observed excess deaths, and testing different hypothetical scenarios of funding and efficiency improvement which may be able to close the mortality gap.

By setting, deaths at care homes and at home contributed most to the observed 'mortality gap', while hospital mortality was lower-than-expected. This is most likely because social care experienced greater relative spending constraints than healthcare. Furthermore, the recent drive to move patients with poor prognoses and who have reached their ceiling of care away from the hospital environment to care homes or their own homes may have contributed to this.[25] It is also important to consider the architectural differences between health and social care delivery in England. The NHS provides publicly delivered universal health coverage, with studies suggesting this confers a protective effect during episodes of substantial cost-constraint.[26] In contrast, social care is means-tested and often privately delivered, without universal coverage; factors that may influence access and quality.

For peer review only - http://bmjopen.bmj.com/site/about/guidelines.xhtml

The associations observed between PEH, PES, and mortality were independent of macroeconomic changes that often co-occur with periods of reduced public-sector spending. However, adjustment for potential mediating factors, nursing numbers in particular, nullified the associations between PEH, PES, and mortality. Our study suggests that the number of NHS-qualified nurses is the strongest tested mediator of the relationships between spending, and care-home and home mortality; this is congruent with findings in other reports.[27]

This study has several policy implications. First, it demonstrates that decelerated increases in public expenditure on healthcare and social care, in England, may have adversely affected population mortality as demand increased and healthcare costs rose above inflation. This demonstrates that while health system design and ambition, such as delivery of universal coverage, is important, it must be adequately financed to ensure design translates into health improvement. Second, the finding that the elderly population and those in care homes were the most vulnerable to recent financial challenges, makes a strong case for targeted interventions to ensure adequate management of these patient groups.[4] This includes funding increases in social care, in addition to maintenance or rises in nursing numbers aligned with demand. Third, and perhaps most importantly, there remains a prospective cost to the current trajectory of system financing that entails a number of excess deaths. While it would be presumptive to class these deaths as avoidable (we have used the terms ‘additional’ or ‘excess’ to describe higher-than-expected numbers), we have outlined several funding-efficiency scenarios that attempt to demonstrate how such a gap could be closed. Given that the health system has historically achieved 1–2% annual productivity improvements, and that current demand is unprecedented, it seems unlikely that greater annual improvements could be expected. After factoring planned Government spending, and the £2 billion funding increase to

For peer review only - http://bmjopen.bmj.com/site/about/guidelines.xhtml

social care announced in the 2017 Spring Budget, our analyses suggest that a cumulative spending increase of approximately £25.3 billion would be required to close this gap across health and social care by 2020/21, equating to around £6.3 billion annually. Future studies may also be needed to provide in-depth investigations on how funding allocations across PEH and PES may influence projected mortality rates.

This is the first study to provide an in-depth analysis of the potential effects of constraints in public expenditure on health and social care, on mortality. In this regard, we did not intend to analyze general changes in government policy, priorities, or the overall environment in which health and social spending operate. A limitation was that our study was observational and retrospective, thereby our findings reflect association rather than causation. Future studies combining different countries or regions with and without spending constraints may allow shock-based causal strategies or natural experiments which mimic the random assignment in clinical trials. We were unable to analyse specific causes of death as outcomes because there were differences in how causes of death in 2001-2010 and 2010 onwards were coded, resulting in a lack of comparability for causes of death such as circulatory disease.[28] Finally, between 2010 and 2012, nurse numbers dropped by approximately 6,000, which, by our regression analyses, translates to approximately 10% of explained deaths for that period. Therefore, the changes in NHS-qualified nurse numbers must only be partly responsible for the putative relationship between spending and mortality. Other resources and complex, emergent behaviour, which do not depend solely on the component parts of the health and social care system but rather on their interactions with one another, might also explain some of the additional deaths observed.

For peer review only - http://bmjopen.bmj.com/site/about/guidelines.xhtml

CONCLUSION

We have found that spending constraints since 2010, especially public expenditure on social care, may have produced a substantial mortality gap in England. Our analyses demonstrate that if demand-side solutions are infeasible, large improvements in efficiency or, more feasibly, spending above growth-in-demand (and not just general inflation) are required to close this gap. We suggest that spending should be targeted on improving care delivered in care homes and at home; and maintaining or increasing nurse numbers.

For peer review only

For peer review only - http://bmjopen.bmj.com/site/about/guidelines.xhtml

AUTHOR CONTRIBUTIONS

JW and MM conceived and designed the study. JW, WW, CDZ, DM and MM obtained the data. JW, WW, GDS, PDR, and VAM conducted data formatting, and JW and WW carried out statistical analysis with input from LPK. All authors helped interpret the findings. JW, WW, and MM wrote the first draft of the manuscript with input from DM, CDZ, RR, and LPK; all authors provided input to subsequent drafts. All authors had full access to all of the data in the study and can take responsibility for its integrity and the accuracy of the data analysis.

ACKNOWLEDGEMENTS

The authors would like to thank Mr. Andrew Pring of Public Health England for extracting data used in the place-of-death analyses and Ms. Lauren Passby and Ms. Peggy Fooks for help regarding the initial data collection effort.

FUNDING

No funding was received for this study.

COMPETING INTERESTS

MM is a co-founder of Cera, a technology-enabled homecare provider.

ETHICAL APPROVAL

Not required.

DATA SHARING

All data are in the public domain.

For peer review only

For peer review only - http://bmjopen.bmj.com/site/about/guidelines.xhtml

REFERENCES

1. Ham C, Imison C, Goodwin N, Dixon A, South P. Where next for the NHS reforms? The case for integrated care [Internet]. London: The King's Fund; 2011. Available from: https://www.kingsfund.org.uk/sites/files/kf/where-next-nhs-reforms-case-for-integrated-care-ham-imison-goodwin-dixon-south-kings-fund-may-2011.pdf
2. Lutz W, O'Neill BC, Scherbov S. Demographics. Europe's population at a turning point. *Science*. 2003 Mar 28;**299**(5615):1991–2.
3. Okunade AA, Murthy VNR. Technology as a 'major driver' of health care costs: A cointegration analysis of the Newhouse conjecture. *J Health Econ*. 2002;**21**:147–159.
4. NHS England, Care Quality Commission, Public Health England, Monitor, Health Education England, NHS Trust Development Authority. NHS five year forward view. [Internet]. NHS England; 2014. Available from: https://www.england.nhs.uk/wp-content/uploads/2014/10/5yfv-web.pdf
5. Marmot M. Social determinants of health inequalities. *Lancet*. 2005;**365**(9464):1099–1104.
6. Bradley EH, Sipsma H, Taylor LA. American health care paradox - high spending on health care and poor health. *Qjm* [Internet]. 2016;(October 2016):hcw187. Available from: https://academic.oup.com/qjmed/article-lookup/doi/10.1093/qjmed/hcw187
7. Local Government Association. Adult social care funding: 2016 state of the nation report [Internet]. London: LGA; 2016. Available from: http://www.local.gov.uk/documents/10180/7632544/1+24+ASCF+state+of+the+nation+2016_WEB.pdf/e5943f2d-4dbd-41a8-b73e-da0c7209ec12
8. Miller C, Bunnin A, Rayner V. Older people who self fund their social care: A guide for health and wellbeing boards and commissioners. London: Office For Public Management; 2013.
9. Office for National Statistics. Population projections [Internet]. 2014. Available from: https://www.ons.gov.uk/peoplepopulationandcommunity/populationandmigration/populationprojections
10. Donnelly L. A&E crisis deepens with 65 hospital trusts issuing emergency alerts [Internet]. 2017. Available from: http://www.telegraph.co.uk/news/2017/01/13/ae-crisis-deepens-58-hospital-trusts-issuing-emergency-alerts/
11. NHS England. A&E Attendances and Emergency Admissions [Internet]. Available from: https://www.england.nhs.uk/statistics/statistical-work-areas/ae-waiting-times-and-activity/
12. UK Parliament. Impact of the Spending Review on health and social care [Internet]. 2016. Available from: https://www.publications.parliament.uk/pa/cm201617/cmselect/cmhealth/139/13902.htm?utm_source=139&utm_medium=fullbullet&utm_campaign=Modulereports
13. Hiam L, Dorling D, Harrison D, McKee M. Why has mortality in England and Wales been increasing? An iterative demographic analysis. *J R Soc Med*. 2017;
14. Office for National Statistics. Mortality statistics: deaths registered in England and Wales [Internet]. 2014. Available from: http://webarchive.nationalarchives.gov.uk/20160105160709/http://www.ons.gov.uk/ons/rel/v

For peer review only - http://bmjopen.bmj.com/site/about/guidelines.xhtml

sob1/mortality-statistics--deaths-registered-in-england-and-wales--series-dr-/index.html

15. UK Health and Social Care Information Centre. NHS Outcomes Framework Indicators [Internet]. *May 2015* 2015. Available from: http://content.digital.nhs.uk/nhsof

16. HM Treasury. Public Expenditure Statistical Analyses 2015 [Internet]. London: Williams Lea Group; 2015. Available from: https://www.gov.uk/government/uploads/system/uploads/attachment_data/file/539465/PESA_2016_Publication.pdf

17. NHS Digital. Personal Social Services: Expenditure and Unit Costs, England [Internet]. 2016. Available from: https://www.gov.uk/government/statistics/personal-social-services-expenditure-and-unit-costs-england-2015-to-2016

18. Office for National Statistics. Unemployment in England, 1992-2015 [Internet]. 2015. p. https://www.nomisweb.co.uk/reports/lmp/gor/2092957. Available from: https://www.ons.gov.uk/employmentandlabourmarket/peoplenotinwork/unemployment

19. Office for National Statistics. Consumer Price Inflation, release [Internet]. 2014. Available from: http://webarchive.nationalarchives.gov.uk/20160105160709/http://ons.gov.uk/ons/rel/cpi/consumer-price-indices/december-2014/index.html

20. Wu VY, Shen Y-C, Yun M-S, Melnick G. Decomposition of the drivers of the U.S. hospital spending growth, 2001-2009. *BMC Health Serv Res* [Internet]. 2014 May 21;**14**:230. Available from: http://www.ncbi.nlm.nih.gov/pubmed/24886580

21. Aiken LH, Sloane DM, Bruyneel L, et al. Nurse staffing and education and hospital mortality in nine European countries: a retrospective observational study. *Lancet* [Internet]. 2014 May;**383**(9931):1824–1830. Available from: http://linkinghub.elsevier.com/retrieve/pii/S0140673613626318

22. NHS England. NHS Five Year Forward View. Recap briefing for the Health Select Committee on technical modelling and scenarios [Internet]. 2016. Available from: https://www.england.nhs.uk/wp-content/uploads/2016/05/fyfv-tech-note-090516.pdf

23. Maruthappu M, Watkins J, Noor AM, et al. Economic downturns , universal health coverage , and cancer mortality in high-income and middle-income countries , 1990 – 2010: a longitudinal analysis. *Lancet* [Internet]. Elsevier Ltd; 2016;**6736**(16):1–12. Available from: http://dx.doi.org/10.1016/S0140-6736(16)00577-8

24. Loopstra R, McKee M, Katikireddi SV, Taylor-Robinson D, Barr B, Stuckler D. Austerity and old-age mortality in England: a longitudinal cross-local area analysis, 2007-2013. *J R Soc Med* [Internet]. 2016 Mar;**109**(3):109–16. Available from: http://www.ncbi.nlm.nih.gov/pubmed/26980412

25. Department of Health. NHS-funded Nursing Care Practice Guide [Internet]. 2013. Available from: https://www.gov.uk/government/uploads/system/uploads/attachment_data/file/211256/NHS-funded_Nursing_Care_Best_Practice_Guidance.pdf

26. Karanikolos M, Mladovsky P, Cylus J, et al. Financial crisis, austerity, and health in Europe. *Lancet (London, England)* [Internet]. Elsevier Ltd; 2013 Apr 13 [cited 2014 Jul 27];**381**(9874):1323–31. Available from: http://www.ncbi.nlm.nih.gov/pubmed/23541059

For peer review only - http://bmjopen.bmj.com/site/about/guidelines.xhtml

27. Mid Staffordshire NHS Foundation Trust. Final Report [Internet]. 2013. Available from: http://webarchive.nationalarchives.gov.uk/20150407084003/http://www.midstaffspublicinquiry.com/report

28. Office for National Statistics (ONS), Office for National Statistics. Results from the ICD – 10 v2010 bridge coding study. *Stat Bull* [Internet]. London; 2011;(February):1–8. Available from: http://webarchive.nationalarchives.gov.uk/20160105160709/http://ons.gov.uk/ons/rel/subnational-health3/results-of-the-icd-10-v2010-bridge-coding-study--england-and-wales--2009/2009/statistical-bulletin--results-of-the-bridge-coding-study.pdf

For peer review only

Table 1. Associations between public expenditure in health (PEH) or social care (PES) and care home deaths. Analyses were performed for 0 to 2 years of interval between PEH or PES and subsequent care-home deaths.

	Lag[1] (year)	Care home deaths per 100,000 persons			
		PEH per capita (£10)		PES per capita (£10)	
		β (95% CI)	p -value	β (95% CI)	p-value
**Model 1**	0	-0.19 (-0.38 to -0.01)	0.05	-5.10 (-6.54 to -3.65)	<0.0001
	1	-0.09 (-0.28 to 0.11)	0.39	-3.54 (-5.43 to -1.65)	0.001
	2	0.06 (-0.13 to 0.25)	0.52	-1.38 (-3.54 to 0.79)	0.23
**Model 2**	0	-0.63 (-0.78 to -0.47)	<0.0001	-5.21 (-6.49 to -3.93)	<0.0001
	1	-0.49 (-0.65 to -0.33)	<0.0001	-4.34 (-5.70 to -2.99)	<0.0001
	2	-0.22 (-0.44 to -0.01)	0.05	-2.73 (-4.40 to -1.06)	0.005
**Model 3**	0	-0.88 (-1.11 to -0.65)	<0.0001	-5.59 (-7.06 to -4.11)	<0.0001
	1	-0.82 (-1.01 to -0.63)	<0.0001	-5.24 (-6.60 to -2.26)	<0.0001
	2	-0.55 (-0.78 to -0.32)	0.0002	-3.82 (-5.39 to -2.26)	<0.0001
**Model 4**	0	0.14 (-0.01 to 0.29)	0.08	-6.23 (-8.10 to -4.39)	<0.0001
	1	0.37 (0.16 to 0.56)	0.002	-7.05 (-9.55 to -4.56)	<0.0001
	2	0.60 (0.35 to 0.85)	0.0001	-7.40 (-10.31 to -4.49)	<0.0001

Number of observations (sex-years) per analysis is 28.
Model 1: Unadjusted model
Model 2: Adjusted for basic state pension per week
Model 3: Adjusted for unemployment rate and CPI
Model 4: PEH and PES were included in the same model
[1]Lag year of which PEH or PES preceded mortality rates

Table 2. Associations of public expenditure in social care (PES) and resources as potential mediating factors with care home deaths. Analyses were conducted for 0 to 2 years of interval between PES and subsequent care-home deaths.

Potential mediators	Lag[1] (year)	Care-home deaths per 100,000 persons			
		PES in 10 million (£)		Potential mediator (thousands)[2]	
		β (95% CI)	p-value	β (95% CI)	p-value
No. of hospital doctors	0	-6.01 (-7.64 to -4.38)	<0.0001	0.21 (0.006 to 0.41)	0.06
	1	-6.66 (-8.65 to -4.38)	<0.0001	0.57 (0.32 to 0.83)	0.0002
	2	-6.85 (-9.03 to -4.67)	<0.0001	0.94 (0.64 to 1.24)	<0.0001
No. of GPs	0	-6.01 (-7.66 to -4.36)	<0.0001	0.87 (-0.009 to 1.76)	0.06
	1	-6.52 (-8.53 to -4.51)	<0.0001	2.35 (1.25 to 3.45)	0.0004
	2	-6.28 (-8.42 to -4.15)	<0.0001	3.67 (2.43 to 4.90)	<0.0001
No. of nurses	0	-4.49 (-6.38 to -2.59)	0.0001	-0.09 (-0.28 to 0.09)	0.34
	1	-0.84 (-2.69 to 1.01)	0.39	-0.40 (-0.58 to -0.29)	0.0001
	2	1.80 (-0.12 to 3.73)	0.08	-0.48 (-0.67 to -0.29)	<0.0001
No. of scientific, therapeutic and technical staff	0	-6.15 (-7.83 to -4.48)	<0.0001	-0.20 (0.01 to 0.38)	0.04
	1	-6.89 (-8.96 to -4.82)	<0.0001	0.52 (0.28 to 0.76)	0.0002
	2	-7.12 (-9.31 to -4.93)	<0.0001	0.85 (0.59 to 1.11)	<0.0001
No. of ambulance staff	0	-6.09 (-7.73 to -4.46)	<0.0001	2.27 (0.17 to 4.37)	0.04
	1	-5.94 (-8.11 to -3.77)	<0.0001	4.60 (1.78 to 7.42)	0.004
	2	-5.09 (-7.70 to -2.47)	0.001	6.72 (3.14 to 10.29)	0.001
No. of clinical support staff	0	-5.01 (-6.74 to -3.38)	<0.0001	-0.02 (-0.26 to 0.22)	0.85
	1	-1.72 (-3.75 to 0.31)	0.11	-0.42 (-0.69 to -0.14)	0.007
	2	-0.49 (-1.87 to 2.86)	0.69	-0.43 (-0.75 to -0.11)	0.02
No. of infrastructure support staff	0	-5.78 (-8.63 to -2.91)	0.0006	0.09 9-0.23 to 0.40)	0.59
	1	-5.31 (-9.43 to -1.20)	0.02	0.21 (-0.23 to 0.66)	0.35
	2	-6.55 (-10.71 to -2.40)	0.006	0.62 (0.18 to 1.06)	0.01
No. of overnight beds	0	-5.56 (-7.10 to -4.19)	<0.0001	-0.13 (-0.25 to -0.008)	<0.0001
	1	-5.42 (-6.95 to -3.90)	<0.0001	-0.34 (-0.48 to -0.21)	<0.0001
	2	-4.38 (-5.92 to -2.84)	<0.0001	-0.50 (-0.65 to -0.35)	<0.0001
No. of social care staff with accommodation	0	-5.20 (-6.64 to -3.77)	0.01	0.09 (-0.05 to 0.23)	0.21
	1	-4.12 (-5.94 to -2.29)	0.01	0.22 (0.02 to 0.42)	0.04
	2	-2.03 (-4.21 to 0.16)	0.01	0.22 (-0.03 to 0.47)	0.09
No. of social care staff without accommodation	0	-5.69 (-7.19 to -4.20)	<0.0001	0.02 (-0.0002 to 0.04)	0.06
	1	-5.52 (-7.21 to -3.83)	<0.0001	0.05 (0.03 to 0.07)	0.0003
	2	-4.67 (-6.30 to -3.09)	0.009	0.08 (0.05 to 0.10)	<0.0001

[1]Lag year of which PEH or PES preceded mortality rates
[2]Estimates are shown for each corresponding factorin the left-hand column.

For peer review only - http://bmjopen.bmj.com/site/about/guidelines.xhtml

FIGURE LEGENDS

Figure 1: Time-trend projections of ASDR per 100,000 individuals. ASDR (left hand y-axis) and the difference in the number of deaths between actual and predicted mortality (right hand y-axis) per year from 2001 to 2014 are shown. The black and blue lines represent actual ASDR for the 2001–10 and 2011–14 periods, respectively. The red line represents predicted ASDR using 2001–10 as an observation base while the 95% CIs are denoted by the beige-colored area. The grey bars denote the differences between the number of deaths observed and the number predicted for 2011–14 where positive values correspond to excess deaths and negative values represent lower-than-expected deaths. Error bars represent 95% CIs. *P < 0.05, **P < 0.01 and ***P < 0.001.

Figure 2: Numbers of excess or lower-than-expected deaths for each place of death. Separate time-trend analyses comparing actual to predicted mortality from 2011 to 2014 were conducted using mortality data categorized by place of death. Contributions from each place of death are color-coded. Data are shown for mortality rates for all ages (top panel), those under 60 (middle panel), and those 60 years or over (bottom panel). *P < 0.05, **P < 0.01 and ***P < 0.001.

Figure 3: Additional spending needed to close the 2020 mortality gap. Data are shown for PEH (blue) and on top of that, PES (beige), in real terms according to 2014/15 prices. Actual outturn data are shown for 2001/02 to 2014/15. However, for 2015/16 to 2020/21, the budgeted total Department of Health expenditure limit is shown for PEH. For PES, data are based on the continuation of -2.25% annual percentage change in core PES in 2014/15 supplemented by the potential revenue from the adult social care precept for council tax.[12] Additional spending needed to close the projected mortality gap for each year from 2015 to 2020 is shown as color-coded dotted lines for three different scenarios, each of which assumes different annual efficiency improvements. The additional annual spending numbers with associated 95% CI are shown as bar plots for each scenario.

SUPPLEMENTARY FIGURE LEGENDS

Figure S1: Public-sector expenditure on healthcare (PEH) and social care (PES). Data are shown from 2001/02–2014/15. Top plot shows data for PEH whereas bottom plot shows data for

For peer review only - http://bmjopen.bmj.com/site/about/guidelines.xhtml

PES. Solid lines correspond to total PEH or PES in billions of pounds (left hand *y*-axis). Dotted lines correspond to PEH or PES in pounds per capita (right hand y-axis) calculated from PEH/PES and population size as estimated by the UK's Office for National Statistics (ONS). Blue lines indicate nominal expenditure. Red lines indicate real expenditure adjusted to 2014/15 prices.

Figure S2: Time-trend projections of mortality rates by place of death. Mortality rates (y-axis) per year from 2001 to 2014 are shown for five places of death with 'Other' representing any place of death that doesn't fall into the other four categories (data are unavailable for a breakdown of where these other places might be). The black and blue lines represent actual ASDR for the 2001–10 and 2011–14 periods, respectively. The red line represents predicted ASDR using 2001–10 as an observation base while the 95% CIs are denoted by the beige-colored area.

Figure S3: **Population mortality projections to 2020**. Age-standardised death rates (ASDR) (left hand y-axis) and difference in the number of deaths between actual and predicted rates (right hand y-axis) projected annually to 2020 are shown. Black lines indicate 2009–14 base-fitted (black solid line) and 2015–20 base-forecasted (black dotted line) values. Red lines indicate the same using an observation base for the years 2001–2010. The bisque-coloured area denotes the 95% CIs for the model for 2009–14 data, and the pink area, the 95% CIs for the model for 2001–10 data. The grey bars represent the difference between the number of deaths projected using a 2009–14 observation base and the number forecasted using a 2001–10 observation base, where positive values correspond to excess deaths. Error bars signify 95% CIs. $^{*}P < 0.05$, $^{**}P < 0.01$ and $^{***}P < 0.001$.

For peer review only

For peer review only - http://bmjopen.bmj.com/site/about/guidelines.xhtml

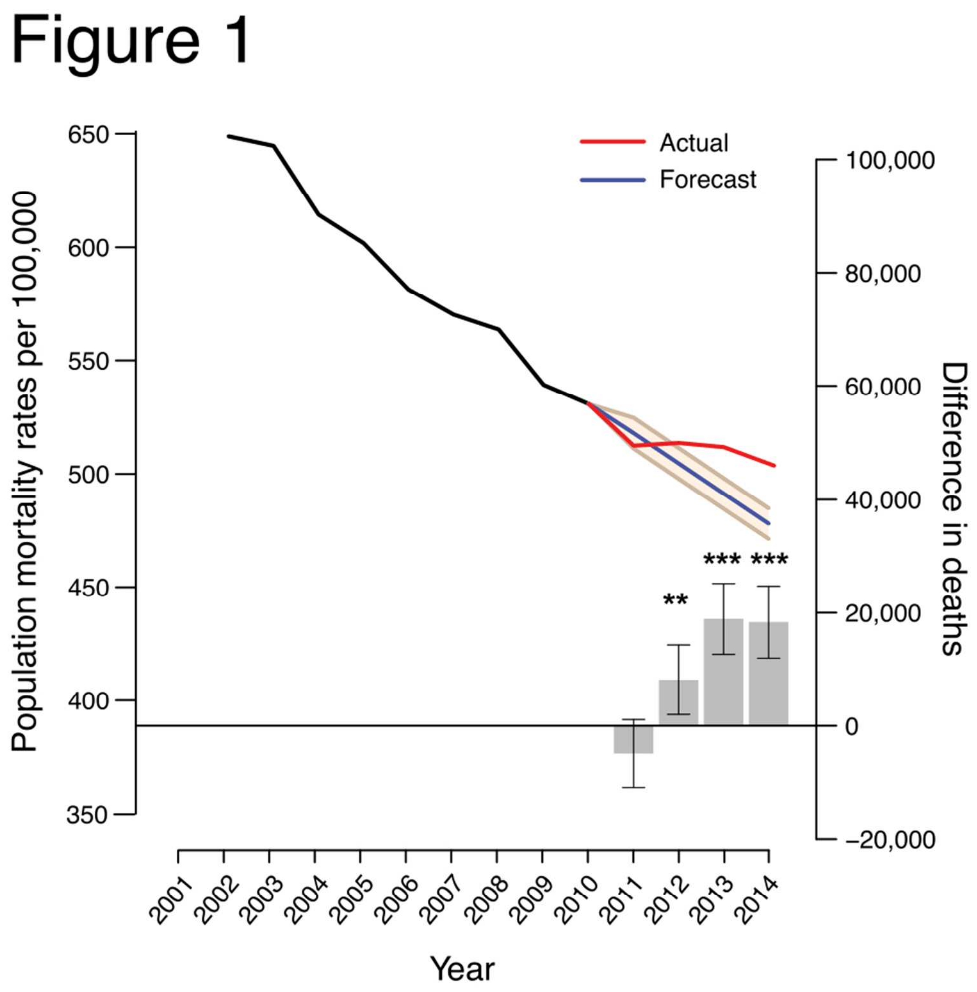

Figure 2

84x84mm (300 x 300 DPI)

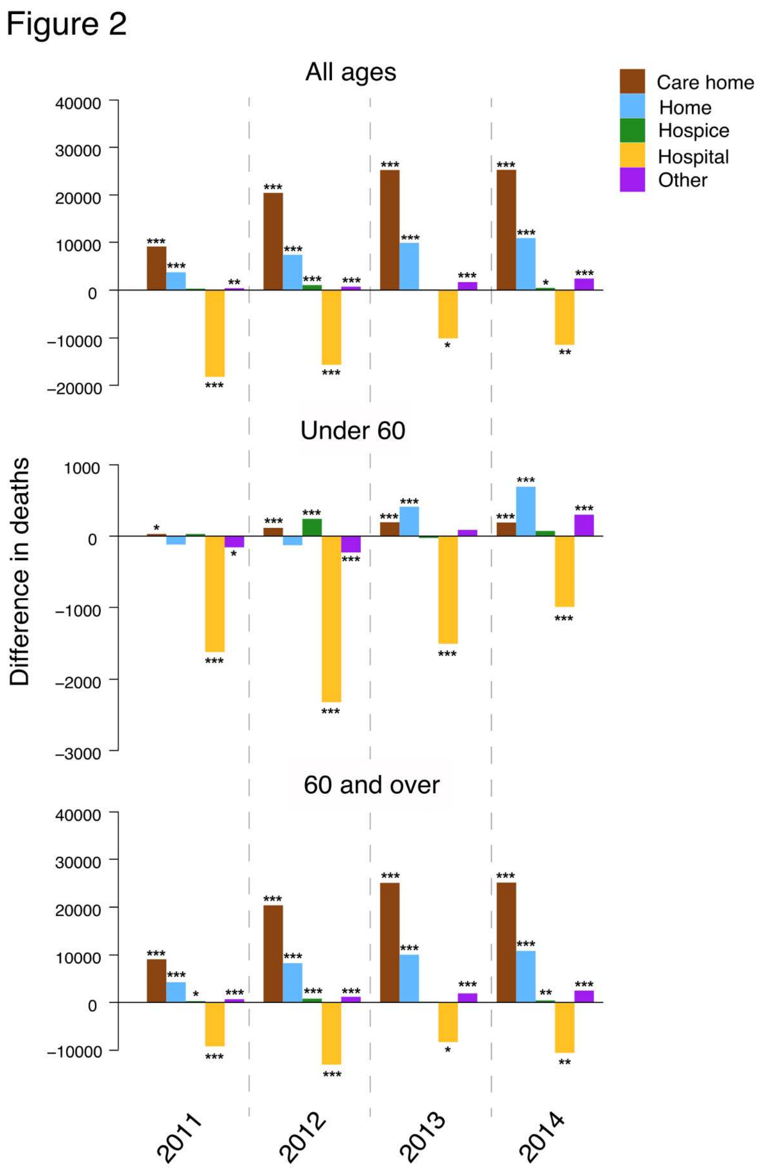

Figure 2

140x208mm (300 x 300 DPI)

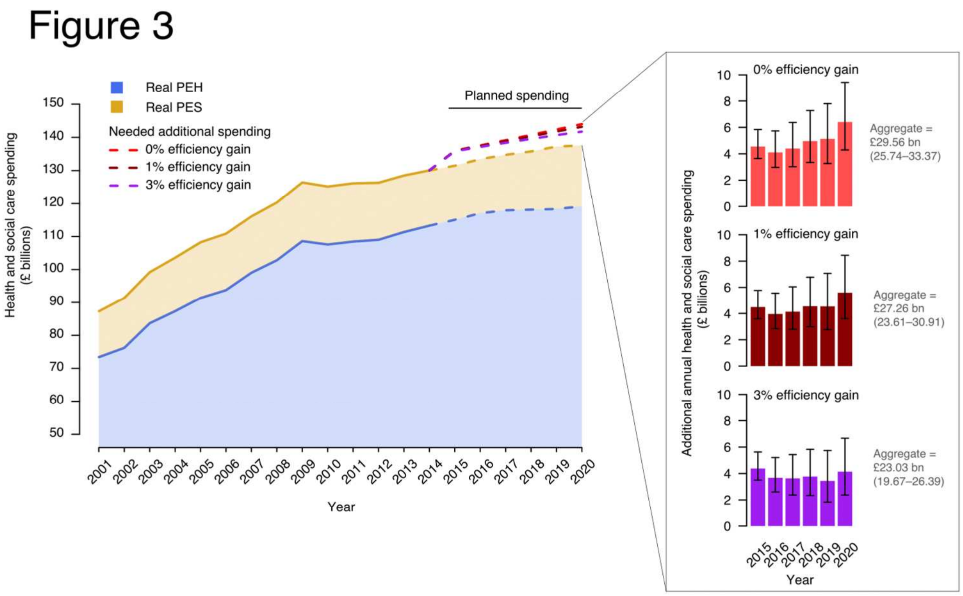

Figure 3

144x89mm (300 x 300 DPI)

Table S1. Absolute and relative differences between observed and predicted age-standardised death rates overall and by sex, 2011-2014.

	No. of observed deaths	Difference in deaths (95% CI)	Rate ratio (95% CI)	p-value
**Both men and women**				
2011	452860	-4875.2 (-10850 to 1099.8)	0.99 (0.98 to 1.00)	0.11
2012	466780	8147.8 (2003.6 to 14292)	1.02 (1.00 to 1.03)	0.009
2013	473550	18896 (12641 to 25152)	1.04 (1.03 to 1.06)	<0.0001
2014	468880	18324 (11953 to 24695)	1.04 (1.03 to 1.06)	<0.0001
**Men**				
2011	219070	-1591.2 (-3917.4 to 734.98)	0.99 (0.98 to 1.00)	0.18
2012	224460	3710.4 (1312 to 6108.8)	1.02 (1.01 to 1.03)	0.0003
2013	229290	10166 (7709.7 to 12622)	1.05 (1.03 to 1.06)	<0.0001
2014	229120	11298 (8777.2 to 13819)	1.05 (1.04 to 1.06)	<0.0001
**Women**				
2011	233790	-2888.6 (-6692.4 to 915.16)	0.99 (0.97 to 1.00)	0.14
2012	242320	5343.6 (1444.8 to 9242.3)	1.02 (1.01 to 1.04)	0.01
2013	244260	9530 (5574.5 to 13486)	1.04 (1.02 to 1.06)	<0.0001
2014	239760	8054.3 (4052.6 to 12056)	1.03 (1.02 to 1.05)	<0.0001

For peer review only

Table S2. Predicted and observed life expectancy (years) overall and by sex, 2010-2014.

	Life expectancy (years)		p-value
	Predicted	Observed	
**2010-2012**			
Male	79.05 (78.98 to 79.12)	79.03	0.71
Female	82.95 (82.86 to 83.04)	82.83	0.08
**2011-2013**			
Male	79.37 (79.27 to 79.47)	79.23	0.009
Female	83.21 (83.08 to 83.33)	82.97	0.0007
**2012-2014**			
Male	79.69 (79.57 to 79.81)	79.37	<0.0001
Female	83.46 (83.31 to 83.62)	83.03	<0.0001

For peer review only

Table S3. Absolute and relative differences between predicted and observed age-standardised PYLL overall and by sex, 2011-2014.

	No. of observed PYLL	Difference in PYLL (95% CI)	Rate ratio (95% CI)	p-value
**Both men and women**				
2011	1110600	5878.8 (214.85 to 11543)	1.01 (1.00 to 1.01)	0.04
2012	1074400	-9359.3 (-15124 to -3594.2)	0.99 (0.99 to 1.00)	0.002
2013	1093200	22568 (16650 to 28485)	1.02 (1.02 to 1.03)	<0.0001
2014	1193000	75496 (69069 to 81923)	1.07 (1.06 to 1.07)	<0.0001
**Men**				
2011	610170	2882.3 (-3093.8 to 8858.5)	1.00 (0.99 to 1.01)	0.35
2012	583320	1794.9 (-4152 to 7741. 8)	1.00 (0.99 to 1.01)	0.57
2013	600650	27893 (21797 to 33989)	1.05 (1.04 to 1.06)	<0.0001
2014	654510	44309 (37538 to 51080)	1.07 (1.06 to 1.08)	<0.0001
**Women**				
2011	500460	3368.2 (487.67 to 6248.7)	1.01 (1.00 to 1.01)	0.02
2012	491090	17219 (14373 to 20064)	1.04 (1.03 to 1.04)	<0.0001
2013	492530	24602 (21688 to 27517)	1.05 (1.05 to 1.06)	<0.0001
2014	538480	32157 (28881 to 35434)	1.06 (1.06 to 1.07)	<0.0001

For peer review only

Table S4. Overall and age group-specific relative differences between observed and predicted death rates, 2011-2014.

	Rate ratio (95% CI)	p-value
**2011**		
All ages (ASR)	0.99 (0.98 to1.00)	0.11
0-34	0.94 (0.92 to 0.96)	<0.0001
35-49	0.96 (0.94 to 0.97)	<0.0001
50-54	0.98 (0.96 to 0.99)	<0.0001
55-59	0.98 (0.97 to 0.99)	0.01
60-64	1.01 (1.01 to 1.02)	0.003
65-69	0.99 (0.98 to 1.00)	0.03
70-74	1.03 (1.02 to 1.05)	<0.0001
75-79	0.99 (0.98 to 1.00)	0.16
80-84	0.96 (0.94 to 0.98)	<0.0001
85 and older	0.99 (0.97 to 1.02)	0.59
**2012**		
All ages (ASR)	1.02 (1.00 to 1.03)	0.01
0-34	0.92 (0.90 to 0.93)	<0.0001
35-49	0.92 (0.91 to 0.93)	<0.0001
50-54	0.96 (0.94 to 0.97)	<0.0001
55-59	0.97 (0.96 to 0.99)	0.0001
60-64	1.04 (1.03 to 1.05)	<0.0001
65-69	1.00 (0.99 to 1.00)	0.32
70-74	1.06 (1.05 to 1.07)	<0.0001
75-79	1.03 (1.02 to 1.04)	<0.0001
80-84	0.99 (0.97 to 1.01)	0.33
85 and older	1.06 (1.03 to 1.08)	<0.0001
**2013**		
All ages (ASR)	1.04 (1.03 to 1.06)	<0.0001
0-34	0.93 (0.91 to 0.94)	<0.0001
35-49	0.94 (0.93 to 0.95)	<0.0001
50-54	0.97 (0.96 to 0.99)	<0.0001
55-59	0.98 (0.96 to 0.99)	<0.0001
60-64	1.08 (1.06 to 1.09)	<0.0001
65-69	1.01 (1.00 to 1.02)	0.02
70-74	1.09 (1.07 to 1.10)	<0.0001
75-79	1.06 (1.05 to 1.08)	<0.0001
80-84	1.01 (0.99 to 1.03)	0.28
85 and older	1.09 (1.06 to 1.11)	<0.0001
**2014**		
All ages (ASR)	1.04 (1.03 to 1.06)	<0.0001
0-34	0.97 (0.95 to 0.99)	0.001
35-49	0.94 (0.93 to 0.95)	<0.0001
50-54	0.98 (0.97 to 1.00)	0.02
55-59	0.97 (0.95 to 0.98)	<0.0001
60-64	1.10 (1.09 to 1.11)	<0.0001
65-69	1.03 (1.02 to 1.04)	<0.0001
70-74	1.11 (1.09 to 1.12)	<0.0001
75-79	1.07 (1.06 to 1.09)	<0.0001
80-84	1.00 (0.98 to 1.02)	0.64
85 and older	1.06 (1.03 to 1.09)	<0.0001

For peer review only

Table S5. Overall and age group-specific relative differences between observed and predicted death rates by place of death, 2011-2014.

	All ages (ASR)		0-59		60+	
	**Rate ratio (95% CI)**	**p-value**	**Rate ratio (95% CI)**	**p-value**	**Rate ratio (95% CI)**	**p-value**
**Care-home**						
2011	1.12 (1.07 to 1.17)	<0.0001	1.03 (1.00 to 1.07)	0.03	1.12 (1.07 to 1.17)	<0.0001
2012	1.26 (1.21 to 1.33)	<0.0001	1.13 (1.10 to 1.17)	<0.0001	1.26 (1.21 to 1.33)	<0.0001
2013	1.33 (1.27 to 1.40)	<0.0001	1.23 (1.19 to 1.27)	<0.0001	1.33 (1.27 to 1.40)	<0.0001
2014	1.33 (1.27 to 1.40)	<0.0001	1.22 (1.18 to 1.26)	<0.0001	1.33 (1.27 to 1.41)	<0.0001
**Home**						
2011	1.04 (1.02 to 1.06)	<0.0001	0.99 (0.98 to 1.01)	0.30	1.05 (1.03 to 1.08)	<0.0001
2012	1.08 (1.06 to 1.10)	<0.0001	0.99 (0.98 to 1.01)	0.26	1.10 (1.08 to 1.13)	<0.0001
2013	1.10 (1.08 to 1.12)	<0.0001	1.03 (1.03 to 1.04)	<0.0003	1.13 (1.11 to 1.15)	<0.0001
2014	1.11 (1.09 to 1.13)	<0.0001	1.04 (1.03 to 1.06)	<0.0001	1.14 (1.11 to 1.16)	<0.0001
**Hospital**						
2011	0.93 (0.90 to 0.96)	<0.0001	0.94 (0.92 to 0.96)	<0.0001	0.93 (0.90 to 0.96)	<0.0001
2012	0.94 (0.91 to 0.97)	0.0001	0.91 (0.89 to 0.93)	<0.0001	0.94 (0.89 to 0.93)	0.0007
2013	0.96 (0.93 to 0.99)	0.01	0.94 (0.92 to 0.96)	<0.0001	0.96 (0.93 to 1.00)	0.03
2014	0.95 (0.92 to 0.99)	0.005	0.96 (0.94 to 0.98)	0.0002	0.95 (0.92 to 0.99)	0.008
**Hospice**						
2011	1.01 (1.00 to 1.03)	0.09	1.01 (0.99 to 1.02)	0.49	1.01 (1.00 to 1.03)	0.04
2012	1.04 (1.00 to 1.03)	<0.0001	1.04 (1.03 to 1.05)	<0.0001	1.04 (1.02 to 1.05)	<0.0001
2013	1.00 (0.99 to 1.02)	0.58	1.00 (0.99 to 1.02)	0.55	1.01 (0.99 to 1.02)	0.29
2014	1.02 (1.00 to 1.03)	0.01	1.01 (1.00 to 1.03)	0.08	1.02 (1.01 to 1.03)	0.006
**Other**						
2011	1.04 (1.02 to 1.07)	0.002	0.96 (0.94 to 0.99)	0.01	1.14 (1.09 to 1.19)	<0.0001
2012	1.08 (1.05 to 1.11)	<0.0001	0.95 (0.92 to 0.98)	0.0003	1.27 (1.21 to 1.33)	<0.0001
2013	1.20 (1.16 to 1.23)	<0.0001	1.02 (0.99 to 1.05)	0.17	1.48 (1.40 to 1.57)	<0.0001
2014	1.30 (1.26 to 1.34)	<0.0001	1.07 (1.04 to 1.10)	<0.0001	1.71 (1.61 to 1.83)	<0.0001

For peer review only

Table S6. Changes in home deaths per 100,000 persons for every £10 change in public expenditure in health (PEH) or social care (PES). Associations were evaluated for up to 2 years of interval between PEH or PES and subsequent home deaths. Number of observations (sex-years) per analysis is 28.

	Lag (year)	PEH per capita (£10)		PES per capita (£10)	
		β (95% CI)	p -value	β (95% CI)	p-value
**Model 1**	0	-0.13 (-0.21 to -0.05)	0.005	-2.63 (-3.27 to -1.99)	<0.0001
	1	-0.07 (-0.15 to 0.02)	0.13	-1.78 (-2.54 to -1.01)	0.0002
	2	0.005 (-0.07 to 0.08)	0.90	-0.71 (-1.58 to 0.16)	0.12
**Model 2**	0	-0.31 (-0.39 to -0.22)	<0.0001	-2.65 (-3.30 to -2.01)	<0.0001
	1	-0.25 (-0.33 to -0.15)	<0.0001	-2.05 (-2.69 to -1.40)	<0.0001
	2	-0.20 (-0.29 to -0.11)	0.0002	-1.22 (-1.93 to -0.52)	0.003
**Model 3**	0	-0.41 (-0.53 to -0.29)	<0.0001	-2.68 (-3.40 to -1.96)	<0.0001
	1	-0.36 (-0.46 to -0.26)	<0.0001	-2.32 (-3.09 to -1.54)	<0.0001
	2	-0.25 (-0.35 to -0.14)	0.0002	-1.17 (-2.56 to -0.88)	0.0008
**Model 4**	0	0.02 (-0.05 to 0.09)	0.62	-2.78 (-3.65 to -1.91)	<0.0001
	1	0.12 (0.03 to 0.21)	0.01	-2.96 (-4.06 to -1.86)	<0.0001
	2	0.21 (0.09 to 0.32)	0.002	-2.79 (-4.11 to -1.47)	0.0005

Model 1: Unadjusted model
Model 2: Adjusted for basic state pension per week
Model 3: Adjusted for unemployment rate and CPI
Model 4: PEH and PES were included in the same model

For peer review only

Table S7. Changes in hospital deaths per 100,000 persons for every £10 change in public expenditure in health (PEH) or social care (PES). Associations were evaluated for up to 2 years of interval between PEH or PES and subsequent hospital deaths. Number of observations (sex-years) per analysis is 28.

	Lag (year)	PEH per capita (£10)		PES per capita (£10)	
		β (95% CI)	p -value	β (95% CI)	p-value
**Model 1**	0	-2.60 (-3.11 to -2.08)	<0.0001	-11.13 (-2.62 to 2.35)	0.12
	1	-2.70 (-3.11 to -2.29)	<0.0001	-19.92 (-31.80 to -8.04)	0.003
	2	-2.66 (-2.30 to -2.35)	<0.0001	-24.02 (-33.30 to -14.74)	<0.0001
**Model 2**	0	-1.36 (-1.79 to -0.94)	<0.0001	-9.11 (-13.57 to -4.65)	0.0005
	1	-1.66 (-1.94 to -1.38)	<0.0001	-12.69 (-15.96 to -9.42)	0.0002
	2	-1.78 (-2.07 to -1.49)	<0.0001	-13.21 (-15.49 to -10.94)	<0.0001
**Model 3**	0	-0.33 (-0.76 to 0.11)	0.16	-1.39 (-4.24 to 1.46)	0.35
	1	-0.07 (-0.12 to -0.03)	0.003	-6.37 (-9.14 to -3.60)	0.01
	2	-0.11 (-0.16 to -0.05)	0.001	-10.14 (-12.81 to -7.48)	0.0006
**Model 4**	0	-3.56 (-3.96 to -3.15)	<0.0001	17.98 (12.95 to 23.02)	<0.0001
	1	-3.69 (-4.11 to -3.27)	<0.0001	15.49 (10.31 to 20.66)	<0.0001
	2	-3.36 (-3.84 to -2.89)	<0.0001	9.73 (4.11 to 15.35)	0.003

Model 1: Unadjusted model
Model 2: Adjusted for basic state pension per week
Model 3: Adjusted for unemployment rate and CPI
Model 4: PEH and PES were included in the same model

For peer review only

Table S8. Changes in home deaths per 100,000 persons associated with changes in public expenditure in social care (PES), adjusted for quantity of resources. Associations were evaluated for up to 2 years of interval between real PES per capita and subsequent home deaths. Number of observations (sex-years) per analysis is 28.

Resource	Lag (year)	Home deaths per 100,000 persons			
		PES per capita (£10)		Resource quantity (thousands)[1]	
		β (95% CI)	p-value	β (95% CI)	p-value
No. of hospital doctors	0	-2.76 (-3.53 to -1.98)	<0.0001	0.03 (-0.07 to 0.12)	0.57
	1	-2.86 (-3.76 to -1.97)	<0.0001	0.20 (-0.08 to 0.31)	0.003
	2	-2.74 (-3.72 to -1.76)	<0.0001	0.35 (0.21 to 0.49)	<0.0001
No. of GPs	0	-2.73 (-3.52 to -1.95)	<0.0001	0.10 (-0.32 to 0.52)	0.0005
	1	-2.76 (-3.68 to -1.84)	<0.0001	0.78 (-0.28 to 1.28)	0.001
	2	-2.51 (-3.47 to -1.55)	<0.0001	1.34 (0.79 to 1.90)	0.002
No. of nurses	0	-2.65 (-3.51 to -1.79)	<0.0001	0.002 (-0.08 to 0.09)	0.96
	1	-0.92 (-1.79 to -0.05)	0.05	-0.13 (-0.21 to -0.04)	0.007
	2	0.45 (-0.40 to 1.30)	0.31	-0.17 (-0.26 to -0.09)	0.0005
No. of scientific, therapeutic and technical staff	0	-2.76 (-3.57 to -1.96)	<0.0001	0.02 (-0.06 to 0.11)	0.60
	1	-2.91 (-3.86 to -1.97)	<0.0001	0.18 (0.07 to 0.28)	0.004
	2	-2.80 (-3.82 to -1.78)	<0.0001	0.31 (0.18 to 0.43)	<0.0001
No. of ambulance staff	0	-2.69 (-3.49 to -1.90)	<0.0001	0.14 (-0.87 to 1.16)	0.79
	1	-2.60 (-3.54 to -1.66)	<0.0001	1.58 (0.36 to 2.80)	0.02
	2	-2.09 (-3.18 to -0.99)	0.001	2.49 (0.99 to 3.99)	0.004
No. of clinical support staff	0	-2.53 (-3.30 to -1.77)	<0.0001	-0.03 (-0.13 to 0.08)	0.63
	1	-1.11 (-1.97 to -0.26)	0.02	-0.15 (-0.27 to -0.04)	0.02
	2	-0.10 (-1.10 to 0.90)	0.85	-0.14 (-0.28 to -0.005)	0.05
No. of infrastructure support staff	0	-2.24 (-3.51 to -0.97)	0.002	-0.05 (-0.19 to 0.09)	0.49
	1	-2.01 (-3.71 to -0.31)	0.03	0.03 (-0.15 to 0.21)	0.76
	2	-2.46 (-4.21 to -0.71)	0.01	0.21 (0.02 to 0.40)	0.04
No. of overnight beds	0	-2.71 (-3.41 to -2.02)	<0.0001	-0.02 (-0.08 to 0.04)	0.522
	1	-2.44 (-3.14 to -1.75)	<0.0001	-0.12 (-0.18 to -0.06)	0.0009
	2	-1.79 (-2.52 to -1.07)	<0.0001	-0.18 (-0.25 to -0.11)	<0.0001
No. of social care staff with accommodation	0	-2.63 (-3.29 to -1.97)	<0.0001	-0.003 (-0.07 to 0.06)	0.94
	1	-2.03 (-2.76 to -1.31)	<0.0001	0.10 (0.03 to 0.18)	0.02
	2	-1.05 (-1.88 to -0.22)	0.02	0.12 (0.02 to 0.21)	0.0002
No. of social care staff without accommodation	0	-2.71 (-3.42 to -1.99)	<0.0001	0.002 (-0.007 to 0.01)	0.61
	1	-2.43 (-3.20 to -1.65)	<0.0001	0.02 (0.006 to 0.03)	0.006
	2	-1.87 (-2.66 to -1.08)	0.0002	0.03 (0.02 to 0.04)	0.0002

[1]Estimates are shown for each corresponding resource in the left-hand column.

For peer review only

Table S9. Changes in care-home deaths per 100,000 persons associated with changes in public expenditure in healthcare (PEH), adjusted for quantity of resources. Associations were evaluated for up to 2 years of interval between real PES per capita and subsequent care-home deaths. Number of observations (sex-years) per analysis is 28.

Resource	Lag (year)	Care-home deaths per 100,000 persons			
		PES per capita (£10)		Resource quantity (thousands)[1]	
		β (95% CI)	p-value	β (95% CI)	p-value
No. of hospital doctors	0	-1.59 (-2.30 to -0.87)	0.0002	2.17 (1.08 to 3.26)	0.003
	1	-1.72 (-2.43 to -1.01)	<0.0001	2.62 (1.51 to 3.73)	0.002
	2	-1.39 (-2.20 to -0.59)	0.003	2.41 (1.11 to 3.71)	0.001
No. of GPs	0	-1.48 (-2.28 to -0.68)	0.001	8.69 (3.41 to 13.97)	0.01
	1	-1.64 (-2.35 to -0.94)	0.0002	10.66 (5.92 to 15.41)	0.0002
	2	-1.37 (-2.05 to -0.69)	0.0008	9.96 (5.35 to 14.57)	0.0004
No. of nurses	0	-0.15 (-0.30 to -0.002)	0.07	-0.35 (-0.54 to -0.17)	0.001
	1	-0.01 (-0.04 to 0.11)	0.83	-0.45 (-0.59 to -0.31)	<0.0001
	2	0.16 (0.04 to 0.28)	0.02	-0.42 (-0.56 to -0.28)	<0.0001
No. of scientific, therapeutic and technical staff	0	-1.53 (-2.42 to -0.64)	0.003	1.87 (0.64 to 3.09)	0.006
	1	-1.58 (-2.45 to -0.71)	0.002	2.12 (0.91 to 3.32)	0.002
	2	-1.30 (-2.15 to -0.45)	0.007	1.96 (0.76 to 3.16)	0.004
No. of ambulance staff	0	-0.37 (-0.78 to 0.04)	0.09	3.17 (-3.39 to 9.72)	0.35
	1	-0.24 (-0.65 to 0.18)	0.28	2.69 (-3.90 to 9.29)	0.43
	2	-0.07 (-0.46 to 0.33)	0.74	2.39 (-3.93 to 8.71)	0.47
No. of clinical support staff	0	-0.15 (-0.33 to 0.02)	0.10	-0.33 (-0.63 to -0.03)	0.04
	1	-0.02 (-0.17 to 0.11)	0.69	-0.55 (-0.78 to -0.31)	0.0002
	2	0.12 (-0.04 to 0.28)	0.16	-0.43 (-0.68 to -0.18)	0.003
No. of infrastructure support staff	0	0.25 (0.004 to 0.50)	0.06	-0.74 (-1.08 to -0.41)	0.0002
	1	0.38 (0.08 to 0.68)	0.02	-0.72 (-1.11 to -0.32)	0.002
	2	0.33 (-0.09 to 0.74)	0.14	-0.37 (-0.89 to 0.15)	0.18
No. of overnight beds	0	-0.84 (-1.12 to -0.52)	<0.0001	-0.76 (-1.09 to -0.42)	0.0002
	1	-0.84 (-1.08 to -0.59)	<0.0001	-0.92 (-1.19 to -0.65)	<0.0001
	2	-0.60 (-0.85 to -0.35)	0.0001	-0.86 (-1.15 to -0.57)	<0.0001
No. of social care staff with accommodation	0	-0.29 (-0.50 to -0.08)	0.01	0.21 (-0.04 to 0.46)	0.11
	1	-0.15 (-0.36 to -0.06)	0.18	0.19 (-0.09 to 0.47)	0.20
	2	-0.02 (-0.18 to -0.23)	0.83	0.13 (-0.14 to 0.41)	0.35
No. of social care staff without accommodation	0	-0.96 (-1.34 to -0.58)	<0.0001	0.13 (0.07 to 0.20)	0.0003
	1	-0.96 (-1.28 to -0.64)	0.0003	0.16 (0.10 to 0.21)	<0.0001
	2	-0.79 (-1.06 to -0.52)	0.0003	0.16 (0.11 to 0.21)	<0.0001

[1]Estimates are shown for each corresponding resource in the left-hand column.

For peer review only

Table S10. Changes in home deaths per 100,000 persons associated with changes in public expenditure in healthcare (PEH), adjusted for quantity of resources. Associations were evaluated for up to 2 years of interval between real PES per capita and subsequent home deaths. Number of observations (sex-years) per analysis is 28.

Resource	Lag (year)	Home deaths per 100,000 persons			
		PES per capita (£10)		Resource quantity (thousands) [1]	
		β (95% CI)	p-value	β (95% CI)	p-value
No. of hospital doctors	0	-0.70 (-1.05 to -0.36)	0.0005	0.89 (0.36 to 1.42)	0.003
	1	-0.75 (-1.06 to -0.44)	0.0001	1.09 (0.61 to 1.58)	0.0002
	2	-0.67 (-0.97 to -0.37)	0.0003	1.11 (0.62 to 1.61)	0.0003
No. of GPs	0	-0.61 (-1.00 to -0.22)	0.005	3.26 (0.69 to 5.83)	0.02
	1	-0.65 (-0.98 to -0.32)	0.0009	4.00 (1.76 to 6.23)	0.002
	2	-0.62 (-0.89 to -0.36)	0.0002	4.37 (2.57 to 6.16)	0.0001
No. of nurses	0	-0.11 (-0.18 to -0.04)	0.006	-0.15 (-0.23 to -0.06)	0.004
	1	-0.03 (-0.10 to 0.02)	0.22	-0.18 (-0.25 to -0.11)	<0.0001
	2	0.04 (-0.01 to 0.10)	0.16	-0.16 (-0.22 to -0.10)	<0.0001
No. of scientific, therapeutic and technical staff	0	-0.59 (-1.02 to -0.15)	0.01	0.64 (0.03 to 1.24)	0.05
	1	-0.64 (-1.03 to -0.25)	0.004	0.81 (0.27 to 1.36)	0.008
	2	-0.59 (-0.93 to -0.25)	0.003	0.86 (0.38 to 1.34)	0.001
No. of ambulance staff	0	-0.14 (-0.34 to 0.05)	0.15	0.25 (-2.79 to 3.29)	0.87
	1	-0.12 (-0.30 to -0.05)	0.19	1.05 (-1.79 to 3.88)	0.48
	2	-0.06 (-0.22 to 0.11)	0.50	1.13 (-1.48 to 3.73)	0.41
No. of clinical support staff	0	-0.11 (-0.19 to -0.03)	0.01	-0.17 (-0.30 to -0.04)	0.02
	1	-0.04 (-0.11 to 0.02)	0.20	-0.23 (-0.33 to -0.12)	0.0003
	2	0.03 (-0.04 to 0.10)	0.48	-0.16 (-0.27 to -0.05)	0.01
No. of infrastructure support staff	0	0.08 (-0.02 to 0.19)	0.14	-0.36 (-0.50 to -0.21)	<0.0001
	1	0.15 (0.02 to 0.27)	0.03	-0.32 (-0.49 to -0.16)	0.0007
	2	0.11 (-0.06 to 0.28)	0.22	-0.15 (-0.36 to 0.07)	0.19
No. of overnight beds	0	-0.40 (-0.55 to -0.25)	<0.0001	-0.32 (-0.48 to -0.16)	0.0007
	1	-0.38 (-0.49 to -0.27)	<0.0001	-0.38 (-0.50 to -0.26)	<0.0001
	2	-0.26 (-0.37 to -0.15)	0.0002	-0.34 (-0.47 to -0.21)	<0.0001
No. of social care staff with accommodation	0	-0.16 (-0.26 to -0.06)	0.004	0.07 (-0.05 to 0.19)	0.25
	1	-0.10 (-0.18 to -0.01)	0.04	0.10 (-0.02 to 0.22)	0.11
	2	-0.02 (-0.10 to 0.06)	0.61	0.09 (-0.02 to 0.20)	0.13
No. of social care staff without accommodation	0	-0.45 (-0.63 to -0.27)	<0.0001	0.06 (0.03 to 0.09)	0.001
	1	-0.40 (-0.56 to -0.25)	<0.0001	0.06 (0.04 to 0.09)	0.0001
	2	-0.31 (-0.45 to -0.18)	0.0001	0.06 (0.04 to 0.08)	<0.0001

[1]Estimates are shown for each corresponding resource in the left-hand column.

For peer review only

Table S11. Changes in care-home and home deaths per 100,000 persons associated with change in no. of nurses. Associations were evaluated for up to 2 years of interval between no. of nurses and subsequent home deaths. Number of observations (sex-years) per analysis is 28.

Place of deaths	Lag (year)	No. of nurses (thousands)	
		β (95% CI)	p-value
Care-home deaths per 100,000 persons	0	-0.38 (-0.57 to -0.19)	0.0007
	1	-0.46 (-0.59 to -0.32)	<0.0001
	2	-0.37 (-0.52 to -0.22)	0.0001
Home deaths per 100,000 persons	0	-0.17 (-0.27 to -0.07)	0.003
	1	-0.18 (-0.25 to -0.12)	<0.0001
	2	-0.15 (-0.21 to -0.08)	0.0002

For peer review only

Table S12. Projected excess deaths prevented in 2015-20 by planned health and social spending under different scenarios of annual productivity increases along with the corresponding additional spending needed to completely close the mortality gap. Numbers in parentheses are 95% confidence intervals.

Scenario	Annual productivity (%)	Excess deaths prevented (%)	Additional spending to prevent 100% of excess deaths (£bn)
**2015**			
Conservative	0	23.58 (19.39 to 27.79)	4.548 (3.647 to 5.84)
Moderate	1	23.82 (19.58 to 28.06)	4.49 (3.6 to 5.76)
Aggressive	3	24.29 (19.96 to 28.62)	4.37 (3.5 to 5.63)
**2016**			
Conservative	0	45.3 (37.24 to 53.39)	4.11 (2.97 to 5.73)
Moderate	1	46.21 (37.99 to 54.48)	3.96 (2.85 to 5.55)
Aggressive	3	48.06 (39.51 to 56.61)	3.68 (2.61 to 5.21)
**2017**			
Conservative	0	51.56 (42.4 to 60.77)	4.4 (3.03 to 6.37)
Moderate	1	53.15 (43.68 to 62.62)	4.13 (2.8 to 6.04)
Aggressive	3	56.38 (46.35 to 66.4)	3.63 (2.37 to 5.43)
**2018**			
Conservative	0	53.56 (44.01 to 63.11)	4.96 (3.35 to 7.28)
Moderate	1	55.72 (45.8 to 65.67)	4.55 (2.99 to 6.77)
Aggressive	3	60.29 (49.54 to 71.05)	3.77 (2.34 to 5.83)
**2019**			
Conservative	0	58.33 (47.94 to 68.71)	5.13 (3.27 to 7.8)
Moderate	1	61.28 (50.38 to 72.24)	4.53 (2.76 to 7.07)
Aggressive	3	67.6 (55.56 to 79.67)	3.44 (1.83 to 5.74)
**2020**			
Conservative	0	54.1 (44.46 to 63.74)	6.41 (4.29 to 9.43)
Moderate	1	57.41 (47.2 to 67.67)	5.6 (3.61 to 8.44)
Aggressive	3	64.58 (53.09 to 76.1)	4.14 (2.37 to 6.67)
Ideal	10.79 (7.8 to 14.46)	100	0

For peer review only

SUPPLEMENTARY FIGURE LEGENDS

Figure S1: Public-sector expenditure on healthcare (PEH) and social care (PES). Data are shown from 2001/02–2014/15. Top plot shows data for PEH whereas bottom plot shows data for PES. Solid lines correspond to total PEH or PES in billions of pounds (left hand *y*-axis). Dotted lines correspond to PEH or PES in pounds per capita (right hand y-axis) calculated from PEH/PES and population size as estimated by the UK's Office for National Statistics (ONS). Blue lines indicate nominal expenditure. Red lines indicate real expenditure adjusted to 2014/15 prices.

Figure S2: Time-trend projections of mortality rates by place of death. Mortality rates (y-axis) per year from 2001 to 2014 are shown for five places of death with 'Other' representing any place of death that doesn't fall into the other four categories (data are unavailable for a breakdown of where these other places might be). The black and blue lines represent actual ASDR for the 2001–10 and 2011–14 periods, respectively. The red line represents predicted ASDR using 2001–10 as an observation base while the 95% CIs are denoted by the beige-colored area.

Figure S3: **Population mortality projections to 2020**. Age-standardised death rates (ASDR) (left hand y-axis) and difference in the number of deaths between actual and predicted rates (right hand y-axis) projected annually to 2020 are shown. Black lines indicate 2009–14 base-fitted (black solid line) and 2015–20 base-forecasted (black dotted line) values. Red lines indicate the same using an observation base for the years 2001–2010. The bisque-coloured area denotes the 95% CIs for the model for 2009–14 data, and the pink area, the 95% CIs for the model for 2001–10 data. The grey bars represent the difference between the number of deaths projected using a 2009–14 observation base and the number forecasted using a 2001–10 observation base, where positive values correspond to excess deaths. Error bars signify 95% CIs. *P < 0.05, **P < 0.01 and ***P < 0.001.

For peer review only

Figure S1

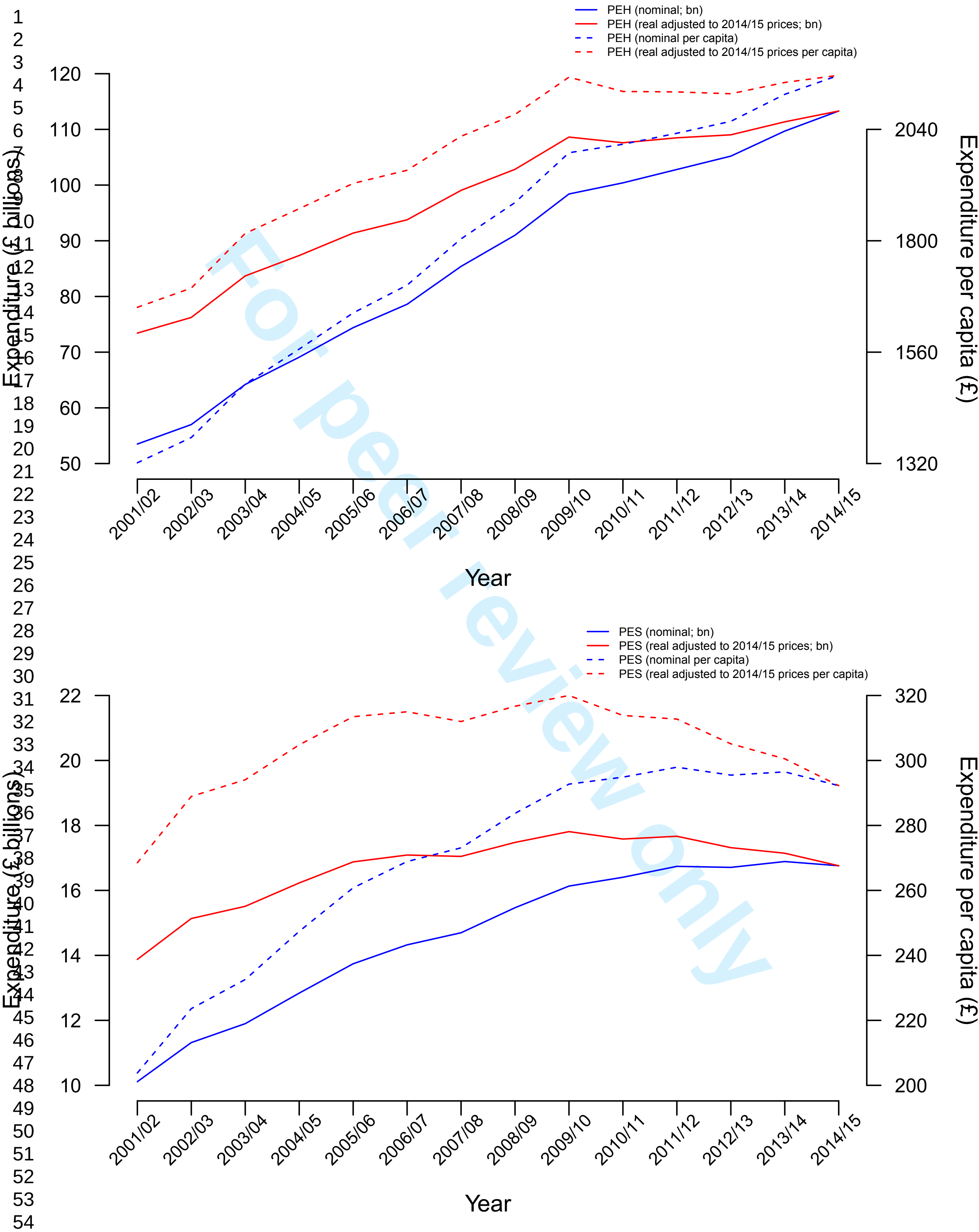

For peer review only

Figure S2

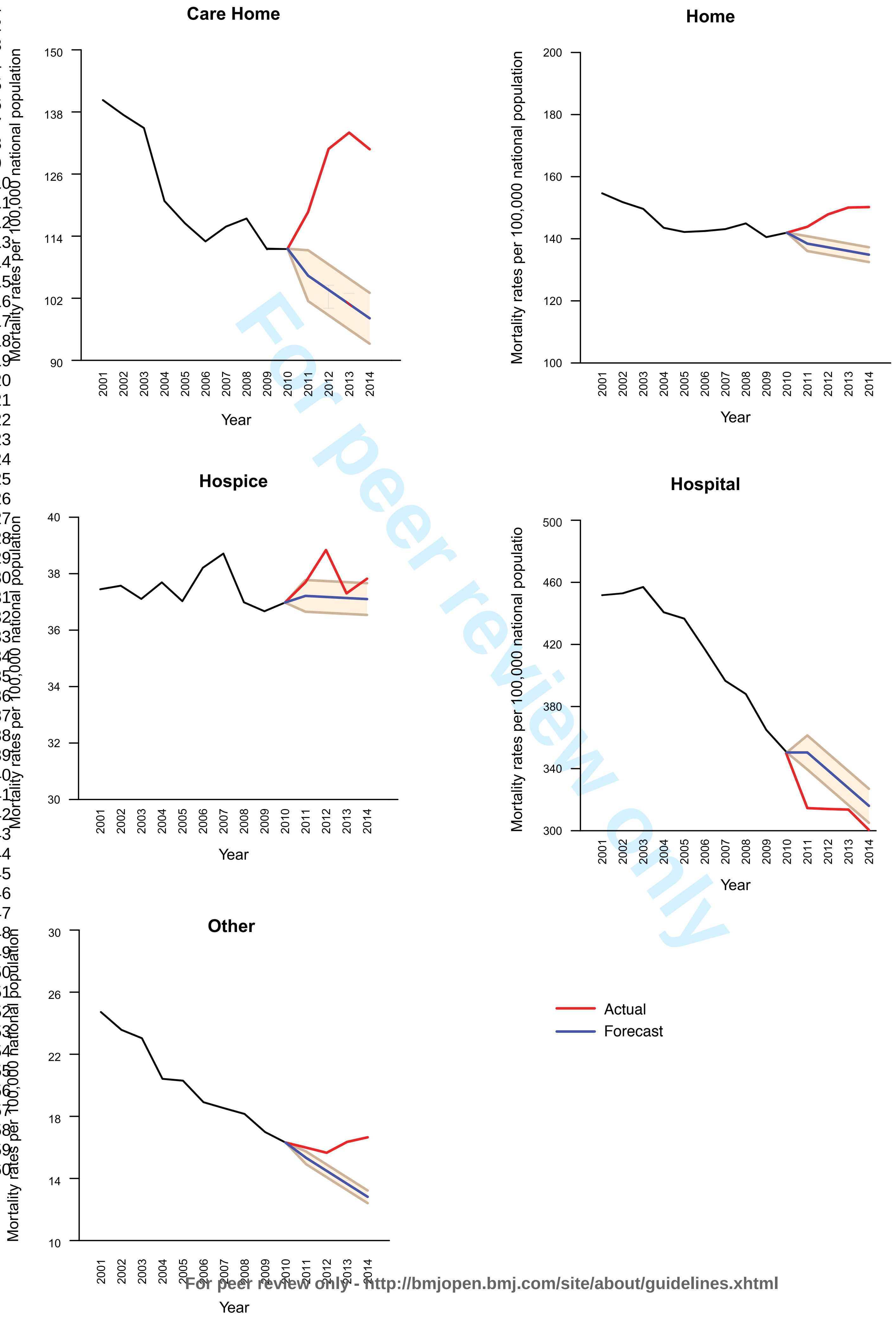

For peer review only

Figure S3

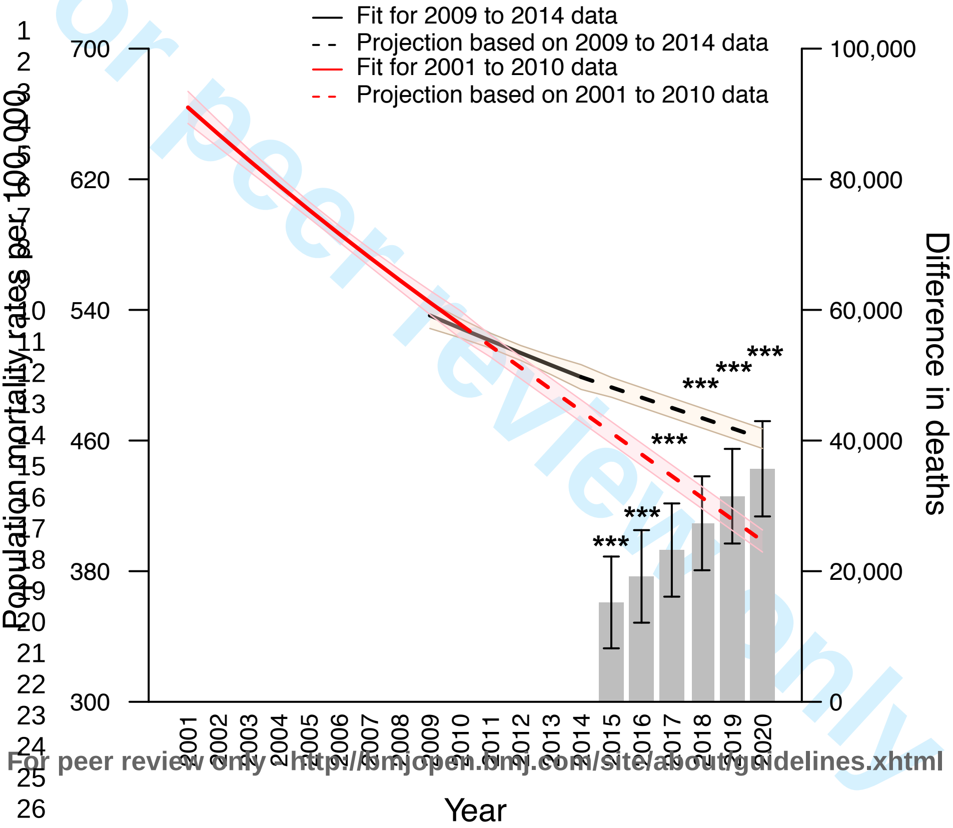

For peer review only - http://bmjopen.bmj.com/site/about/guidelines.xhtml

SUPPLEMENTARY APPENDIX

Data collection and generation

Age-standardized rates

All-cause age-standardized death rates (ASDR) were calculated using the ONS' standardized mortality rate calculation template with the standard European population.[1] The ten age groups used were: 0-34, 35-49, 50-54, 55-59, 60-64, 65-69, 70-74, 75-79, 80-84, and ≥85.

Age-standardized potential years of life lost rates were calculated using the same age groups and by weighting the number of 'amenable' deaths in a given year by the standard European population and the number of additional years that the deceased might have been expected to live in the presence of timely and effective healthcare.[2]

Spending data

To calculate the 2014/15 data point, we used the percentage change in the bridging data between 2013/14 and 2014/15. From this, we calculated real PEH per capita in units of £10. Planned spending from 2015/16 to 2020/21 for healthcare was sourced from the 2015 Spending Review and Autumn Statement.[5] For adult social care spending, no consolidated planned expenditure limit was available. Therefore, we defined 2015/16 to 2020/21 planned real PES based on the continuation of –2.25% change in core PES in 2014/15 supplemented by the potential revenue from the adult social care precept, which is intended to raise funds through 2% per annum increases in local government tax revenues.[6]

Deprivation data

Indices of Multiple Deprivation (IMD), a composite measure using data on topics such as employment, education, and crime, were provided for each local government area by the UK Department for Communities and Local Government for 2010[7]. Population-weighted deprivation status was provided as the average rank of the ranks of the five IMD variables, ranging from 1 to 325, in order of decreasing deprivation.

Resource data

For peer review only

Health and social care staff and bed numbers (resource data) available for 2001 to 2014 were obtained for the following: the number of NHS hospital doctors; general practitioners (GPs); qualified NHS health and social care nurses; qualified NHS scientific, therapeutic and technical staff; NHS ambulance staff; NHS health and social care clinical support staff; NHS infrastructure support staff[8,9]; the number of overnight beds in NHS hospitals[10]; and the numbers of staff providing social care with accommodation and without (see Supplementary Appendix)[11,12].

Employment data in social work activities with accommodation (e.g. care homes) and without accommodation provision (e.g. home care) in England between 2001 and 2014 were obtained from the UK labour market statistics provided by the ONS[11,12]. The 2003 version of Standard Industrial Classification (SIC) codes (SIC 2003) were used to identify businesses conducting social work activities with and without accommodation (SIC 2003 code: 8531 and 8532, respectively). Prior to 2009, data was available from the Annual Business Enquiry, whereas the Business Register Employment Survey provided data from 2009 onwards under the SIC 2007 codes. Proportional mapping using ONS-published proportions between SIC 2003 and SIC 2007 was conducted to estimate the number of employment which corresponds to the aforementioned SIC 2003 codes between 2009 and 2014.[13]

For peer review only

Time-trend prediction analysis

Mortality and potential years of life lost

Given that consistent mortality data were available from 2001 onwards, and that the reductions in real PEH and PES occurred in 2010/11, we used 2001–2010 as an observation base on which to build our time-trend model for predicting mortality rates in 2011–2014.

Depending on whether overdispersion was observed (where the variance exceeds the mean), we resolved to use either a Poisson or quasi-Poisson regression model, the latter of which incorporates a scaling parameter in order to address overdispersion. In every dataset for which we conducted time-series modelling, the variance of the mortality rates exceeded the mean rate.

Therefore, we employed a quasi-Poisson model in all analyses, with the logarithmic link function preventing the prediction of negative rates. The model takes the following form:

$$E(M_t) = \exp(\alpha + \beta t), \qquad (1)$$

where $E(M_t)$ is the estimated mortality rate for year t, α is the baseline mortality rate, and β is the estimated drift parameter. For increasing observation-base trends, the following non-linear prediction model proposed by Dyba and colleagues,[14] also assuming a Poisson or quasi-Poisson distribution, was used:

$$E(M_t) = \alpha(1 + \beta t). \qquad (2)$$

The trend of a mortality rate was determined to be decreasing or increasing according to whether the geometric mean of the ratio of the annual percentage change was negative or positive, respectively.

Rate ratios for the observed and expected mortality rates were calculated. We also estimated the number of “excess” (higher-than-expected) or “prevented” (lower-than-expected) deaths in 2011, 2012, 2013 and 2014 by finding the difference between the observed number of deaths and the number of deaths predicted by the 2001–2010-based time-series models above. 95% confidence intervals were computed using the standard errors of the fitted mortality rate estimates.

We ran separate analyses using the above model for males, females, and both sexes. The age-stratified time-trend analyses examined 10 age groups. The place-specific mortality rates used the same approach but were split into just two age groups, those 60 and over, and those less than 60 in addition to an all-age group analysis.

For the analyses of potential years of life lost, the same process was repeated but using the directly standardized rates of potential years of life lost as the outcome instead of mortality rates.

Life expectancy

A univariate autoregressive integrated moving average (ARIMA) model was fitted to life expectancy data available from 1998 to 2010, and used to predict data points for 2011 and 2012 as per a method by Torri and Vaupel[15]. In order to optimize the p, d, and q parameters, we conducted

For peer review only

a model search and selected the parameterized model that minimized the Akaike Information Criterion (AIC) value, which is defined as:

$$AIC = 2k - 2\ln(L) \tag{3}$$

where k is 3, i.e. the number of parameters in the ARIMA model, and L is the maximized value of the likelihood function for the model. A drift term was included to account for the clear upward trend in life expectancy over the 1998 to 2010 period.

Aggregating results across years

Aggregate central estimates of deaths across time-trend analyses of each projected year were calculated by addition. For 95% confidence intervals, the root-sum-of-squares method was used:

$$d_{agg_{CI}} = d_{agg} \pm \sqrt{\sum_t^n (d_t - d_{t_{CI}})^2} \tag{4}$$

where $d_{agg_{CI}}$ is the aggregate confidence intervals (lower and upper), d_{agg} is the aggregate central estimate, n is the final year in the projected time series, d_t is the central estimate for year t, and $d_{t_{CI}}$ is the confidence interval (lower or upper) for the year t.

Panel-based regression analysis

Model selection

To assess the suitability of different regression approaches, we separately fitted pooled ordinary least squares (OLS), fixed-effects, and random-effects models to the real PEH per capita and population mortality data with sex as the time-invariant entity. The assumption of the random-effects model that the error terms for unobserved or observed time-invariant variables such as sex are uncorrelated with the independent variables means that sex is therefore also able to serve as an independent variable. In fixed-effects models, this is not the case.

We therefore tested whether the consistent but inefficient fixed-effects model was more suitable than the potentially inconsistent but efficient pooled OLS and random-effects approaches by conducting Hausman tests, both of which were highly significant (P<0.001), leading us to reject

For peer review only

the random-effects and pooled OLS approaches and their assumptions in favour of a fixed-effects model.

Regression model

We therefore used the following fixed-effects regression model:

$$H_{it} = \beta_0 + \beta_1 X_{1it} + \ldots + \beta_k X_{kit} + U_{it} \qquad (5)$$

where H_{it} is the response variable for which i is sex and t is time in years; X_{kit} represents the independent variables; β_k is the coefficient for the independent variables; and U_{it} is the error term. For population-based mortality, H was all-cause ASDR. For the regression analyses in which economic and health resource indicators were not controlled, X was just real PEH or real PES per capita in units of ten pounds sterling. To test whether our results were robust to variations in the economy, we added average annual consumer price index (CPI)[16] and unemployment rate[17] as independent variables to the model. For mediation analyses, each health and social care resource was added as an independent variable.[8–12]

Lag analyses

We conducted 1- and 2-year lag analyses. For an *i*th year lag analysis, regression was performed using the mortality rate in year x and the value of the explanatory variables (real PEH or real PES per capita, etc.) in year $x - i$.

Mortality projections to 2020

For the 2020 projection analysis, two projections were performed each with a different observation base: 2001–10 for one and 2009–14 for the other. Data points for 2009 and 2010 were included for the second observation base to give at least six observations from which to make predictions.Instead of comparing actual rates with trend-determined expected rates, the projected rates from[14] using these two different observation bases were compared against each other to calculate rate ratios, and the number of excess deaths annually between 2015 and 2020. Mortality rates for 2015 to 2020 were standardized against the National Population 2012-based projections.[18]

Scenario modelling

For peer review only - http://bmjopen.bmj.com/site/about/guidelines.xhtml

For the 2020 projection analysis, we assumed that the mortality projections were underpinned by flat real PEH and PES per capita and no change in efficiency since 2014/15. We performed scenario modeling to test how much of the mortality gap could be closed by planned spending under varying assumptions of annual efficiency changes.

To do this, we first performed fixed-effects regression as before but instead using all-cause population ASDR as the outcome variable, and combined real PEH or PES per capita as the explanatory variable. From these analyses, an increase of £10 per capita real spending on health and social care is associated with 2.56 (2.10 to 3.01) lives saved per 100,000.

To calculate the number of lives saved, *l*, for year *t*, which could be any year between 2015 to 2020, inclusive, we used the following formula:

$$l_t = (1+p)^{t-2014} \times \frac{r}{100{,}000} \times \frac{(S_t - S_{2014})}{10} \qquad (6)$$

where p is the assumed annual efficiency change; r is the coefficient (or upper or lower bound of the 95% confidence interval); 100,000 is that the coefficient is for all-cause ASDR per 100,000; S_t represents planned health and social care spending combined for year t and S_{2014} denotes the combined health and social care spending outturn for 2014/15. The difference in spending is divided by 10 to acknowledge that the regression coefficient was obtained with units of £10 changes per capita.

The percentage of lives saved was calculated using the number of excess deaths from the mortality projections for year *t*.

The additional spending needed to completely close the gap, i.e. make sure that 100% of lives were saved for year *t*, was computed as follows:

$$S_{additional} = \frac{d_t \times 1{,}000{,}000}{(1+p)^{t-2014} \times r} - S_t + S_{2014} \qquad (7)$$

where d_t is the excess deaths calculated for t.

For peer review only

For each year between 2015 and 2020, we modeled three different scenarios, each assuming different values for *p*:

1) a conservative 0% annual efficiency change;
2) a moderate 1% annual efficiency gain (1% was the average efficiency to the nearest integer for the period 2001 to 2013 [2014 data were unavailable]); and
3) an aggressive 3% annual efficiency gain (3% was the highest efficiency to the nearest integer reached in the 2001 to 2013 period.

These productivities were defined by using historical and targeted efficiency changes in healthcare as a guide.[19]

In addition, we asked what annual efficiency gain up to 2020 would be needed if there was no spending on top of the planned PEH and PES budgets. This was calculated as follows:

$$p = \left(\frac{d_{2020} \times 1{,}000{,}000}{r \times (S_{2020} - S_{2014})}\right)^{1/(2020-2014)} - 1 \quad (8)$$

REFERENCES

1. Office for National Statistics. Mortality Statistics: Metadata. Newport: ONS; 2015.
2. UK Health and Social Care Information Centre. NHS Outcomes Framework Indicators. May 2015. 2015;Available from: http://content.digital.nhs.uk/nhsof
3. HM Treasury. Public Expenditure Statistical Analyses 2015. London: Williams Lea Group; 2015.
4. NHS Digital. Personal Social Services: Expenditure and Unit Costs, England. 2016;Available from: https://www.gov.uk/government/statistics/personal-social-services-expenditure-and-unit-costs-england-2015-to-2016
5. UK Parliament. Impact of the Spending Review on health and social care. 2016;Available from: https://www.publications.parliament.uk/pa/cm201617/cmselect/cmhealth/139/13902.htm?utm_source=139&utm_medium=fullbullet&utm_campaign=Modulereports
6. UK Parliament. The impact of the Spending Review on health and social care finances. 2016;Available from: https://www.publications.parliament.uk/pa/cm201617/cmselect/cmhealth/139/13906.htm
7. UK Department for Communities and Local Government. English Indices of Deprivation. December 2012. 2012;Available from: https://www.gov.uk/government/collections/english-indices-of-deprivation
8. The NHS Information Centre. NHS workforce: summary of staff in the NHS: results from September 2011 Census. 2012;1–31. Available from:

https://catalogue.ic.nhs.uk/publications/workforce/numbers/nhs-staf-2001-2011-medi-dent/nhs-staf-2001-2011-medi-dent-work-rep.pdf

9. Health and Social Care Information Centre. NHS workforce statistics in England, summary of staff in the NHS - 2004-2014. 2015.

10. NHS England. Overnight bed availability and occupancy data. London: 2014.

11. Office for National Statistics. Business Register Employment Survey, 2009-2014. 2014;Available from: https://www.nomisweb.co.uk/

12. Office for National Statistics. Annual Business Enquiry, 2001-2008. 2008;Available from: https://www.nomisweb.co.uk/

13. Office for National Statistics. Correlation between UK SIC 2003 and UK SIC 2007. 2015;Available from: http://webarchive.nationalarchives.gov.uk/20160105160709/http://www.ons.gov.uk/ons/guide-method/classifications/archived-standard-classifications/uk-standard-industrial-classification-1992--sic92-/index.html

14. Dyba T, Hakulinen T, Päivärinta L. A simple non-linear model in incidence prediction. Stat Med 1997;16(20):2297–309.

15. Torri T, Vaupel JW. Forecasting life expectancy in an international context. Int J Forecast 2012;28:519–31.

16. Office for National Statistics. Consumer Price Inflation, release. 2014;Available from: http://webarchive.nationalarchives.gov.uk/20160105160709/http://ons.gov.uk/ons/rel/cpi/consumer-price-indices/december-2014/index.html

17. Office for National Statistics. Unemployment in England, 1992-2015. 2015;https://www.nomisweb.co.uk/reports/lmp/gor/2092957. Available from: https://www.ons.gov.uk/employmentandlabourmarket/peoplenotinwork/unemployment

18. Office for National Statistics. Time series: England population mid-year estimate. 2015;

19. NHS England. NHS Five Year Forward View. Recap briefing for the Health Select Committee on technical modelling and scenarios. 2016.

For peer review only

CHEERS Checklist
Items to include when reporting economic evaluations of health interventions

The **ISPOR CHEERS Task Force Report**, *Consolidated Health Economic Evaluation Reporting Standards (CHEERS)—Explanation and Elaboration: A Report of the ISPOR Health Economic Evaluations Publication Guidelines Good Reporting Practices Task Force*, provides examples and further discussion of the 24-item CHEERS Checklist and the CHEERS Statement. It may be accessed via the *Value in Health* or via the ISPOR Health Economic Evaluation Publication Guidelines – CHEERS: Good Reporting Practices webpage: http://www.ispor.org/TaskForces/EconomicPubGuidelines.asp

Section/item	Item No	Recommendation	Reported on page No/ line No
**Title and abstract**			
Title	1	Identify the study as an economic evaluation or use more specific terms such as "cost-effectiveness analysis", and describe the interventions compared.	
Abstract	2	Provide a structured summary of objectives, perspective, setting, methods (including study design and inputs), results (including base case and uncertainty analyses), and conclusions.	
**Introduction**			
Background and objectives	3	Provide an explicit statement of the broader context for the study. Present the study question and its relevance for health policy or practice decisions.	
**Methods**			
Target population and subgroups	4	Describe characteristics of the base case population and subgroups analysed, including why they were chosen.	
Setting and location	5	State relevant aspects of the system(s) in which the decision(s) need(s) to be made.	
Study perspective	6	Describe the perspective of the study and relate this to the costs being evaluated.	
Comparators	7	Describe the interventions or strategies being compared and state why they were chosen.	
Time horizon	8	State the time horizon(s) over which costs and consequences are being evaluated and say why appropriate.	
Discount rate	9	Report the choice of discount rate(s) used for costs and outcomes and say why appropriate.	
Choice of health outcomes	10	Describe what outcomes were used as the measure(s) of benefit in the evaluation and their relevance for the type of analysis performed.	
Measurement of effectiveness	11a	*Single study-based estimates:* Describe fully the design features of the single effectiveness study and why the single study was a sufficient source of clinical effectiveness data.	

For peer review only

	11b	*Synthesis-based estimates:* Describe fully the methods used for identification of included studies and synthesis of clinical effectiveness data.	
Measurement and valuation of preference based outcomes	12	If applicable, describe the population and methods used to elicit preferences for outcomes.	
Estimating resources and costs	13a	*Single study-based economic evaluation:* Describe approaches used to estimate resource use associated with the alternative interventions. Describe primary or secondary research methods for valuing each resource item in terms of its unit cost. Describe any adjustments made to approximate to opportunity costs.	
	13b	*Model-based economic evaluation:* Describe approaches and data sources used to estimate resource use associated with model health states. Describe primary or secondary research methods for valuing each resource item in terms of its unit cost. Describe any adjustments made to approximate to opportunity costs.	
Currency, price date, and conversion	14	Report the dates of the estimated resource quantities and unit costs. Describe methods for adjusting estimated unit costs to the year of reported costs if necessary. Describe methods for converting costs into a common currency base and the exchange rate.	
Choice of model	15	Describe and give reasons for the specific type of decision-analytical model used. Providing a figure to show model structure is strongly recommended.	
Assumptions	16	Describe all structural or other assumptions underpinning the decision-analytical model.	
Analytical methods	17	Describe all analytical methods supporting the evaluation. This could include methods for dealing with skewed, missing, or censored data; extrapolation methods; methods for pooling data; approaches to validate or make adjustments (such as half cycle corrections) to a model; and methods for handling population heterogeneity and uncertainty.	
**Results**			
Study parameters	18	Report the values, ranges, references, and, if used, probability distributions for all parameters. Report reasons or sources for distributions used to represent uncertainty where appropriate. Providing a table to show the input values is strongly recommended.	
Incremental costs and outcomes	19	For each intervention, report mean values for the main categories of estimated costs and outcomes of interest, as well as mean differences between the comparator groups. If applicable, report incremental cost-effectiveness ratios.	
Characterising uncertainty	20a	*Single study-based economic evaluation:* Describe the effects of sampling uncertainty for the estimated incremental cost and incremental effectiveness parameters, together with the impact	

For peer review only

		of methodological assumptions (such as discount rate, study perspective).	
	20b	*Model-based economic evaluation:* Describe the effects on the results of uncertainty for all input parameters, and uncertainty related to the structure of the model and assumptions.	
Characterising heterogeneity	21	If applicable, report differences in costs, outcomes, or cost-effectiveness that can be explained by variations between subgroups of patients with different baseline characteristics or other observed variability in effects that are not reducible by more information.	
**Discussion**			
Study findings, limitations, generalisability, and current knowledge	22	Summarise key study findings and describe how they support the conclusions reached. Discuss limitations and the generalisability of the findings and how the findings fit with current knowledge.	
**Other**			
Source of funding	23	Describe how the study was funded and the role of the funder in the identification, design, conduct, and reporting of the analysis. Describe other non-monetary sources of support.	
Conflicts of interest	24	Describe any potential for conflict of interest of study contributors in accordance with journal policy. In the absence of a journal policy, we recommend authors comply with International Committee of Medical Journal Editors recommendations.	

For consistency, the CHEERS Statement checklist format is based on the format of the CONSORT statement checklist

The **ISPOR CHEERS Task Force Report** provides examples and further discussion of the 24-item CHEERS Checklist and the CHEERS Statement. It may be accessed via the *Value in Health* link or via the ISPOR Health Economic Evaluation Publication Guidelines – CHEERS: Good Reporting Practices webpage: http://www.ispor.org/TaskForces/EconomicPubGuidelines.asp

The citation for the CHEERS Task Force Report is:

Husereau D, Drummond M, Petrou S, et al. Consolidated health economic evaluation reporting standards (CHEERS)—Explanation and elaboration: A report of the ISPOR health economic evaluations publication guidelines good reporting practices task force. Value Health 2013;16:231-50.

For peer review only
